# Different depression: motivational anhedonia governs antidepressant efficacy in Huntington’s disease

**DOI:** 10.1093/braincomms/fcac278

**Published:** 2022-11-09

**Authors:** Duncan James McLauchlan, Thomas Lancaster, David Craufurd, David E J Linden, Anne E Rosser

**Affiliations:** Division of Psychological Medicine and Clinical Neurosciences, Cardiff University, Cardiff CF24 4HQ, UK; Department of Neurology, Morriston Hospital, Swansea Bay University Health Board, Swansea SA6 6NL, UK; Division of Psychological Medicine and Clinical Neurosciences, Cardiff University, Cardiff CF24 4HQ, UK; Cardiff University Brain Research Imaging Center, Cardiff University, Cardiff CF24 4HQ, UK; Department of Psychology, University of Bath, Bath BA2 7AY, UK; Manchester Center for Genomic Medicine, Division of Evolution and Genomic Sciences, School of Biological Sciences, Faculty of Biology, Medicine and Health, University of Manchester, Manchester Academic Health Science Center, Manchester M13 9PL, UK; St. Mary’s Hospital, Manchester University NHS Foundation Trust, Manchester Academic Health Science Center, Manchester M13 9WL, UK; Division of Psychological Medicine and Clinical Neurosciences, Cardiff University, Cardiff CF24 4HQ, UK; Cardiff University Brain Research Imaging Center, Cardiff University, Cardiff CF24 4HQ, UK; Department of Psychology, University of Bath, Bath BA2 7AY, UK; School for Mental Health and Neuroscience, Fac. Health, Medicine and Life Sciences, Maastricht University, Maastricht, Netherlands; Division of Psychological Medicine and Clinical Neurosciences, Cardiff University, Cardiff CF24 4HQ, UK; Department of Neurology, Morriston Hospital, Swansea Bay University Health Board, Swansea SA6 6NL, UK; School of Biosciences, Cardiff University, Cardiff CF10 3AX, UK

**Keywords:** Huntington’s disease, depression, antidepressant, cognitive mechanism, propensity scoring

## Abstract

Depression is more common in neurodegenerative diseases such as Huntington’s disease than the general population. Antidepressant efficacy is well-established for depression within the general population: a recent meta-analysis showed serotonin norepinephrine reuptake inhibitors, tricyclic antidepressants and mirtazapine outperformed other antidepressants. Despite the severe morbidity, antidepressant choice in Huntington’s disease is based on Class IV evidence. We used complementary approaches to determine treatment choice for depression in Huntington’s disease: propensity score analyses of antidepressant treatment outcome using the ENROLL-HD data set, and a dissection of the cognitive mechanisms underlying depression in Huntington’s disease using a cognitive battery based on the Research Domain Criteria for Depression. Study 1 included ENROLL-HD 5486 gene-positive adult patients started on an antidepressant medication for depression. Our outcome measures were depression (Hospital Anxiety and Depression Scale or Problem Behaviours Assessment ‘Depressed Mood’ item) at first follow-up (primary outcome) and all follow-ups (secondary outcome). The intervention was antidepressant class. We used Svyglm&Twang in R to perform propensity scoring, using known variables (disease progression, medical comorbidity, psychiatric morbidity, sedatives, number of antidepressants, demographics and antidepressant contraindications) to determine the probability of receiving different antidepressants (propensity score) and then included the propensity score in a model of treatment efficacy. Study 2 recruited 51 gene-positive adult patients and 26 controls from the South Wales Huntington’s Disease Management Service. Participants completed a motor assessment, in addition to measures of depression and apathy, followed by tasks measuring consummatory anhedonia, motivational anhedonia, learning from reward and punishment and reaction to negative outcome. We used generalised linear models to determine the association between task performance and depression scores. Study 1 showed selective serotonin reuptake inhibitors outperformed serotonin norepinephrine reuptake inhibitors on the primary outcome (*P* = 0.048), whilst both selective serotonin reuptake inhibitors (*P* = 0.00069) and bupropion (*P* = 0.0045) were superior to serotonin norepinephrine reuptake inhibitors on the secondary outcome. Study 2 demonstrated an association between depression score and effort for reward that was not explained by apathy. No other mechanisms were associated with depression score. We found that selective serotonin reuptake inhibitors and bupropion outperform serotonin norepinephrine reuptake inhibitors at alleviating depression in Huntington’s disease. Moreover, motivational anhedonia appears the most significant mechanism underlying depression in Huntington’s disease. Bupropion is improves motivational anhedonia and has a synergistic effect with selective serotonin reuptake inhibitors. This work provides the first large-scale, objective evidence to determine treatment choice for depression in Huntington’s disease, and provides a model for determining antidepressant efficacy in other neurodegenerative diseases.

## Teaser

McLauchlan *et al.* used propensity scoring of observational data from the ENROLL-HD study and dissection of cognitive mechanisms in a prospectively recruited cohort. They report that antidepressant classes acting on motivational anhedonia are more efficacious at treating depression in Huntington’s disease.

See Hannan (https://doi.org/10.1093/braincomms/fcac294) for a scientific commentary on this article.

## Graphical Abstract

**Graphical abstract:**
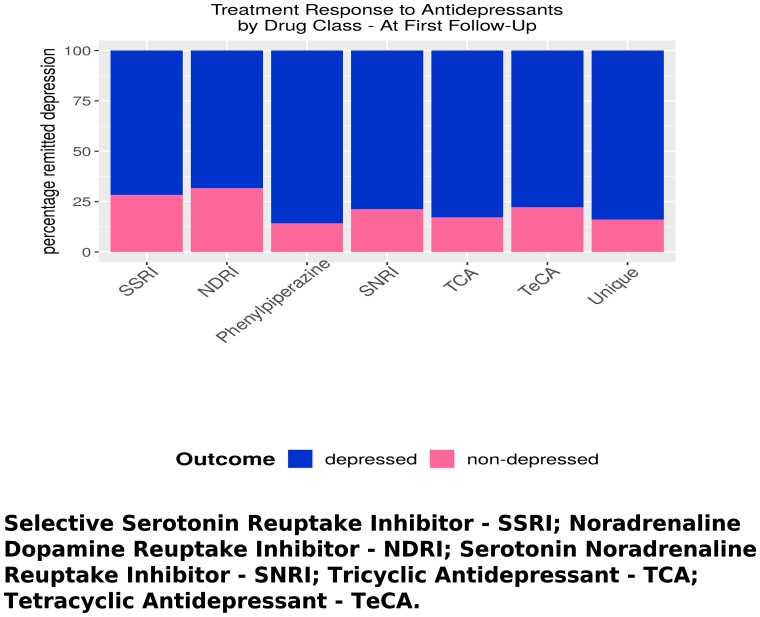

## Introduction

Mood disorder is common in neurodegenerative diseases, with a higher prevalence of depression found in many disorders^1–10^ compared with the general population.^11,12^ Huntington’s disease (a trinucleotide repeat disorder that leads to progressive neurodegeneration of cortico-striatal networks),^13^ causes cognitive impairment and neuropsychiatric disorders that predate the onset of motor symptoms such as chorea.^14–17^

Depression is very common in Huntington’s disease: systematic reviews and large observational studies suggest a lifetime prevalence of 33–69%^6,7,18^ compared with 15% in the general population.^11,12^ Neuropsychiatric symptoms have more impact on quality of life and functional decline in Huntington’s disease than motor symptoms, for both patients and carers.^19–24^ Furthermore, depression in Huntington’s disease can be a direct result of the neurodegeneration, or a reaction to the diagnosis. Higher rates of depression are seen prior to genetic testing in gene carriers compared with gene negative individuals at risk for Huntington’s disease,^17,25^ whilst depression-like behaviour has been found in animal models of Huntington’s disease^26^ that can be rescued with inactivation of the *Huntingtin* mutation.^27^ Such findings suggest that depression is an integral part of the pathology of Huntington’s disease.

Despite the severe morbidity, objective evidence of effective treatment for depression in Huntington’s disease is very limited. Randomized controlled trials of citalopram for executive function and bupropion for apathy both excluded patients with significant depression at entry but still showed a reduction in depression scores in the intervention groups.^28,29^ One open-label study of venlafaxine as a treatment for depression showed a reduction in depression scores^30^ and reports on other antidepressants were limited to case studies.^31–33^

The cognitive–behavioural mechanisms underlying depression in Huntington’s disease are also under-explored. Work using experimental behavioural tasks in MDD has shown that both anhedonia (reduced response to rewarding stimuli) and hypersensitivity to negative stimuli contribute to the development of depression.^34–37^ These findings informed the Research Domain Criteria (RDoC) for Depression^38^: anhedonia may be mediated by deficits in the experience of pleasure (consummatory anhedonia), impaired effort for reward (motivational anhedonia) or impaired learning from rewarded stimuli (anticipatory anhedonia).^35,39–46^ Immediate hypersensitivity to negative stimuli, negative future thinking, low self-esteem and excessive rumination (dwelling on negative outcomes and experiences)^47–50^ also contribute to the depressive phenotype. Because the cognitive–behavioural components of depression have been associated with different neurotransmitter systems,^39,40,51–53^ such a differentiation can also lead to more precise treatment for depressive syndromes.

A recent network meta-analysis demonstrated that the most effective medications for treating MDD were predominantly tricyclic antidepressants (TCAs), serotonin–norepinephrine reuptake inhibitors (SNRIs) and mirtazapine [tetracyclic antidepressants (TeCAs)] classes, with selective serotonin reuptake inhibitors (SSRIs) and bupropion (a noradrenaline–dopamine reuptake inhibitor: NDRI) generally proving less effective.^54^ It is also increasingly recognized that certain antidepressants are more effective at reversing selected cognitive processes underlying MDD compared with others: SSRIs are ineffective at treating (and may even worsen) consummatory anhedonia,^55,56^ whilst drugs that increase noradrenergic tone are associated with increased arousal but may reduce effort for reward.^57–60^

Current treatment options for depression in Huntington’s disease rely on evidence gleaned from MDD; however, the mechanisms of depression in Huntington’s disease may differ: it is known that Huntington’s disease patients are comparatively insensitive to negative stimuli,^61–64^ and the cognitive impairment may limit rumination in Huntington’s disease.^65^ Furthermore depression in other neurodegenerative diseases may not respond to antidepressants in the same way as MDD.^66–68^ Hence the most effective drug classes for depression in Huntington’s disease may also be different to those in MDD.

In the absence of randomized controlled trial (RCT) evidence, propensity score analysis of observational data can provide evidence of the effectiveness of treatments. In observational studies, treatments are selected based on variables such as clinician preference and disease severity. Participants in a propensity score analysis are ascribed a probability of receiving a particular treatment based on known variables (the propensity score), treatments are then compared either by direct matching on propensity score or including the propensity score as an inverse weight in a model. Although this method cannot account for unknown confounders, there is evidence to suggest unknown confounding variables are associated with known confounding variables.^69,70^ Furthermore in contrast to RCTs, a more representative sample of the wider patient population is included, improving generalizability.^71,72^ In this work, we combined a propensity score analysis of the effect of antidepressants on depression scores in a large observational study data set of Huntington’s disease participants (ENROLL-HD^73^) with an experimental study dissecting the mechanisms underlying depression in Huntington’s disease. The aim was to use two complementary approaches to improve our understanding of the neuropsychological mechanisms of depression in Huntington’s disease and inform optimised pharmacological treatment.

## Materials and methods

All study procedures were performed in accordance with the declaration of Helsinki. Formal ethical approval for this work was gained from the NHS Research Ethics Council for Wales (13/WAL/0300) and the NHS Research Ethics Council for Scotland (13/SS/0169).

### Study 1: effect of antidepressant class on depression in Huntington’s disease using propensity score analysis

#### Inclusion criteria

Participants were selected for this analysis from the ENROLL-HD^74^ longitudinal observation study. This is a prospective worldwide cohort study (including participants previously part of the Europe-wide REGISTRY^75^ study), recruiting participants with genetic confirmation of Huntington’s disease, those at risk, gene negative controls and not-at-risk family controls. All participants complete annual assessments of function (Unified Huntington’s disease Rating Scale Total Functional Capacity—UHDRS-TFC^76^), motor examination (Unified Huntington’s disease Rating Scale Total Motor Score—UHDRS-TMS), cognition (Stroop task, symbol digit modality task and verbal fluency^15^), psychiatric symptoms (Problem Behaviours Assessment short form—PBAs,^77^ Hospital Anxiety and Depression Score—HADS,^78^ and the UHDRS—Behavioural Scale^76^) and demographic changes such as comorbidities, medication and drug use.

We included any participant with genetically confirmed Huntington’s disease, started on an antidepressant for low mood (low mood indications: Supplementary Table 1).

#### Intervention: antidepressant class comparison

Antidepressants are typically categorised into selective serotonin reuptake inhibitors (SSRIs), SNRIs, monoamine oxidase inhibitors (MAOIs), TCAs or atypical agents. As sleep disturbance and apathy^14,79–82^ are significant problems in Huntington’s disease, recent clinical guidelines for treatment of Huntington’s disease symptomatology,^83,84^ recommend mirtazapine, trazodone or related antidepressants for the treatment of depression and comorbid insomnia; and a trial of an activating antidepressant such as bupropion is recommended if apathy is present. Our analysis plan therefore included separate classes for (i) tetracyclic antidepressants (TeCA: mirtazapine, mianserin and maprotiline), (ii) phenylpiperazines (trazodone, nefazodone), and (iii) bupropion as a NDRI, in addition to SSRIs, SNRIs, MAOIs and TCAs. Any antidepressant with a unique mechanism of action, accounting for <1% of all prescriptions was included in the ‘Unique’ class for the analysis.

#### Outcome measures

Psychiatric symptoms in ENROLL-HD were measured using the HADS (a self-report measure with good validity for depression in HD^85^) and the PBAs^77^ [a semi-structured interview comprising 12 psychiatric symptom domains commonly seen in Huntington’s disease; each symptom is rated for severity (0–4) and frequency (0–4)], whilst REGISTRY used the UHDRS behaviour score (an earlier iteration of the PBAs semi-structured interview with 12 psychiatric symptom domains seen in Huntington’s disease; each symptom is rated for severity and frequency from 0–4). The ‘Depressed Mood’ item was common to both the PBAs and UHDRS behaviour score. Participants were classified as having depressed mood if they either: (i) scored >7 on the HADS depression score; (ii) had scores of >1 on both the severity and frequency subscores of the PBAs ‘Depressed Mood’ item; or (iii) had severity and frequency subscores greater than 1 for the UHDRS behaviour score ‘Depressed Mood’ item.

We used depressed mood at first follow-up (the next ENROLL or REGISTRY visit following antidepressant start date, provided this occurred at least 10 days after antidepressant prescription and within 1 year) as the primary outcome, and depressed mood at all subsequent follow-ups (including any follow-up visit occurring at least 10 days after antidepressant start date) as the secondary outcome.

#### Statistical analysis: propensity scores

In observational studies, treatments are selected based on inherent participant characteristics such as severity of illness or presence of relevant comorbidities. Propensity scoring measures each participants probability of being assigned a particular treatment based on known variables. This process is known to account for unobserved as well as observed variables, as well as giving a precise effect of each independent variable on treatment allocation.

We used the svyglm and twang packages in R,^86,87^ which employ machine learning to improve model fit to data over 5000 iterations. These packages compare models with different combinations and coefficients for all variables, to determine the best model to describe treatment allocation: that is they create a mathematical model to determine which variables influence treatment selection (choice of antidepressant class). As all participants received an intervention (class of antidepressant), models tested the ‘average treatment effect’ (ATE: which treatment was best for any given member of the study population), rather than the ‘average treatment effect among the treated’ (ATT: used to compare treatment to no intervention in the treated population). Following the propensity scoring model selection, *post hoc* Kolmogorov–Smirnov tests were used to check the effectiveness of the process (Supplementary Tables 27 and 28).

The propensity score variable was then included as an independent variable in a generalized boosted model measuring the effect of antidepressant class on the primary and secondary outcomes. Any variables not completely equalized across treatment groups in the best propensity score model were included in the generalized boosted model as fixed variables in a ‘doubly robust’ process as described by Ridgeway, McCaffrey *et al*.^86^

We selected the variables age, gender, psychiatric morbidity (previous suicide attempt, psychiatric hospitalisation or current high psychiatric comorbidity {any PBAs item score > 4}), total number of antidepressants, number of comorbidities, concurrent benzodiazepine or antipsychotic use, total number of antidepressant prescriptions (either dose escalation or change of drug), dose of antidepressant, composite disease progression score^88^ (a highly sensitive measure of disease progression in Huntington’s disease combining, cognitive, motor and functional scores) and risk factors for SSRI use (cardiac or gastrointestinal disease) in the propensity scoring model. We used ANOVAs to test the effect of antidepressant class on model fit.

### Study 2: cognitive mechanisms of depression in Huntington’s disease

#### Participant recruitment and consent

We recruited 51 Huntington’s disease participants from the South Wales Huntington’s disease service as part of a wider study of neuropsychiatric symptoms in Huntington’s disease^64,89^ (disease stage: pre-symptomatic to Stage IV), and 26 controls were recruited both from family members not at risk of Huntington’s disease, and university staff and students through local advertising. We excluded participants under the age of 18, Huntington’s disease participants without a confirmatory gene test, pregnant women, and any participant with brain injury or brain disorder other than Huntington’s disease. All subjects gave informed consent.

#### General procedures

All subjects completed a medical history, medication history, and formal Huntington’s disease motor examination (total motor score (TMS) from the Unified HD Rating Scale^76^) prior to the questionnaires and neuropsychological tasks. Participants received expenses (maximum £20) for any costs incurred by participation but were specifically informed that the expenses were not related to task performance.

#### Questionnaire assessments of psychopathology

Prior to the neuropsychological task battery all participants completed the Hospital Anxiety and Depression Scale, a robust, well-verified self-report measure of depression in Huntington’s disease ^85^; the Apathy Evaluation Scale (Clinician) to assess apathy^90^; Problem Behaviours Assessment—short form (PBAs): the best validated assessment of psychopathology in Huntington’s disease^14,18,77,79^ (used in the ENROLL-HD study); and an assessment of reward value and impulsivity—the Behavioural Inhibition Scale Behavioural Activation Scale (BISBAS),^91^ from which we used the reward subscale (BISBAS Reward) as a measure of subjective reward value.

#### Neuropsychological assessment procedure

All tasks were programmed in E-Prime 2.0 and performed on a Lenovo Thinkpad laptop. Testing was performed in a quiet environment, free from external distractions. Participants were seated at a comfortable distance from the computer, so they could easily read text and respond appropriately. Breaks were encouraged ad libitum. Tasks were drawn from a larger battery of 14 assessments (including measures of instrumental learning, planning, inhibition, temporal discounting) designed to probe cognitive processes underlying other common psychiatric symptoms in Huntington’s disease (apathy, impulsivity and aggressive behaviour), and were administered in random order.

#### Neuropsychological tasks

This battery encompassed the RDoC domains contributing to depression^92,93^: reward [positive valence systems: measures of reward value (reward responsiveness), effort for reward (motivational anhedonia), and learning from positive outcome)]; and loss (negative valence systems: measures of response to negative outcome, and learning from negative outcome).

#### Reward reaction time task (RRTT)—motivational anhedonia

This task measured willingness to exert effort for reward (known to be impaired in MDD^42,94^) and was based on a protocol by Cools *et al*.^95^ Participants were instructed that the task involved reacting as quickly as possible to a visual stimulus to win a reward (points), and that as they completed each block, the maximum reward on offer would increase. They were further informed that reacting too slowly would result in no reward. Short breaks were given between blocks. The 4 test blocks (each of 30 trials) were preceded by a practice block. Scoring was based on proportional improvement in mean reaction time during the practice block to account for motor disability. The outcome measure was reward sensitivity: change in reaction time with maximum value of reward (corrected for block order).

#### BISBAS reward^74^—reward responsiveness

This well-verified self-report questionnaire measures reward value as one of its four subscales.

#### Probabilistic Selection Learning Task^96^: instrumental learning from reward and punishment

Impaired reward learning and heightened recall of negative outcome are both found in MDD.^97–100^ Participants were shown three different pairs of visual stimuli (Mandarin characters) and asked to select which in each pair is likely to be correct. Participants were told that one stimulus in each pair was correct more often than the other—they must learn which one and select it every time it was displayed. In pair AB, A was correct on 80% of trials; for pair CD, C was correct on 70% of trials and in pair EF, E was correct on 60% of trials. The task included a training phase (which terminated when participants selected the correct symbol in each pair at greater than 60% probability or after 374 trials) and a testing phase of 100 trials when either A or B was paired with one stimulus from C, D, E, and F and participants did not receive feedback. The testing phase showed whether participants learned better from reward (‘choose A’) or from punishment (‘avoid B’). Outcomes were the total score in the testing phase (as a measure of instrumental learning), reward learning score (total of A pairs correct in the testing phase), punishment learning score (total of B pairs correct in the testing phase) and an interaction model to compare punishment and reward learning.

#### Race task—response to negative outcome

This novel task was designed to measure pessimistic future prediction and excessive response to negative feedback, processes known to be abnormal in MDD.^34,50,101–106^ In this task, participants were first asked to perform a timed tapping assessment. They were then shown a race between 2 figures. Immediately following the race participants were asked if they felt they could improve the slower runner’s performance sufficiently to make them win. This involved tapping repeatedly on the keyboard, taking into account their baseline tapping speed. Participants gave a probability of performance improvement from 0 (certain of being unable to make the slower runner win) to 100 (certain of making the slower runner win). Following the rating, participants attempted to speed the slower runner, using rapid keyboard tapping. The slower runner still lost. Participants were then asked again about ability to improve performance if given a second attempt. The outcome variables were predicted performance following negative feedback (0–100) (absolute score and change from baseline rating).

#### Statistical analysis—cognitive mechanism

Missing data was excluded on a pair wise basis. Owing to the time constraints associated with the completion of the wider task battery (of cognitive tasks designed to probe processes leading to impulsive, apathetic and aggressive behaviour), 43 of 51 Huntington’s disease participants completed the race task, whilst motor impairment (5) and software failure (4) further limited RRTT completion to 46. The Probabilistic Selection Learning Task (PSLT) was introduced later in the experiment and was completed by 35 Huntington’s disease participants. All controls completed the full battery. Premorbid IQ tests based on reading ability have been shown to be unreliable in manifest Huntington’s disease,^107–109^ and hence we used Crawford’s demographic method^108^ to calculate premorbid IQ. Serotonergic and dopaminergic medication used by participants was converted to equivalent doses of Fluoxetine and Olanzapine, as described in meta-analytic work on effect sizes.^110,111^

All statistical analyses were completed in R.^87,112–114^ Group comparisons of Huntington’s disease participants and controls used models including case (Huntington’s disease versus control) as a variable. Within the Huntington’s disease group, regression models were constructed to assess the predictive value of neuropsychological task performance on current measures of depression in Huntington’s disease. The assumptions underlying linear regression were formally tested (normality of model residuals, Durbin-Watson test, Goldfeldt-Quandt test) and if the assumptions did not hold, we used a general linear model (GLM). The effect of potential confounding variables (all demographic and psychopathology subscores listed in Table 1) was assessed by adding the potential confounding variable to the regression model and comparing with the simple regression model using a likelihood ratio test. Any variables that improved the model were included as fixed effects in a final multiple regression model. Nested models with, and without group or task performance variables were compared using ANOVAs or likelihood ratio tests depending on the distribution.

**Table 1:** Demographics and neuropsychiatric scores

Enroll-HD cohort 5486 participants			
Age	50.43 (18–92)		
Gender	55.25% female		
Total motor score	33.72 (0–122, 70.70% manifest)		
Disease burden	395.08 (16.5–1020)		
Disease progression score	8.83 (-7.34–23.2)		
Psychiatric comorbidity	97.5%		
Total comorbidities	4 (0–58)		
Number of antidepressants	1 (0–12)		
SSRI risk factor	17.41%		
Sedative use	71.83%		
Antidepressant dose (fluoxetine equivalent—mg)	33.23 (0–150)		
**Cognitive task cohort 77 participants**			
	Case status		
	Huntington’s disease	Controls	Significance
Age	53.27 (33–82)	46.85 (20–75)	
IQ	103.55 (88.75–125.27)	109.73 (89.79–128.51)	*
Gender	26/51 female	17/26 female	
Antipsychotic dose (olanzapine equivalent—mg)	1.92 (0–41.25)	0	***
Antidepressant dose (fluoxetine equivalent—mg)	22.27 (0–146.5)	2.4 (0–22.2)	***
CAG repeat length	42.5 (38–50)	−	
Total motor score	35.49 (0–89, 78.43% manifest)	1.48 (0–6)	***
Disease burden	366.04 (90–575)	0	
Cognitive impairment (phonemic verbal fluency score)	30.41	45.46	***
HADS depression	5.82 (0–17)	1.88 (0–9)	***
AES	38.48 (18–72)	18.85 (18–86)	***
PBA apathy	5.02 (0–16)	0.5 (0–4)	***
PBA perseveration	1.9 (0–12)	0	***
PBA disorientation	2.12 (0–8)	0.12 (0–2)	***
PBA irritability	3.12 (0–12)	0.38 (0–2)	***
PBA aggression	2.08 (0–12)	0.31 (0–4)	***
PBA depressed mood	3.14 (0–12)	1.81 (0–9)	
PBA suicidal ideation	0.37 (0–6)	0.04 (0–1)	
PBA anxiety	2.69 (0–12)	1.69 (0–6)	
PBA obsessions and compulsions	0.8 (0–12)	0.12 (0–3)	
PBA delusions	0.43 (0–9)	0	
PBA hallucinations	0.16 (0–8)	0	

Significance: * < 0.05 ** < 0.01 *** < 0.001.

Means and range (in brackets) are shown.

SSRI, selective serotonin reuptake inhibitor, IQ, full scale intelligence quotient, PBA, Problem Behaviours Assessment (Short Form), BISBAS, Behavioural Inhibition Scale Behavioural Activation Scale; AES, Apathy Evaluation Scale; ‘manifest’, Huntington’s disease participant diagnosed with motor onset.

#### Mixed model analysis—RRTT data

We created Gamma generalized linear mixed models (GLMMs),^115^ with a dependent variable of reaction time and independent variable of maximum reward value. The random effect term was the individual participant. We included TMS to account for motor disability (fixed effect) and block order (as an interaction with maximum reward) to account for the effects of fatigue.^116^ We compared models using Bolker’s method^113,117^: the goodness of fit was assessed using the Akaike information criterion and models were compared for explanatory power using the bbmle package and the weight (explanatory power corrected for complexity, maximum value 1.0) is reported.^113^ To study the effect of case on change in reaction time for different reward, we used data for all participants (cases and controls) and compared models without case status, including case status as a fixed effect, and with case status as an interaction with maximum reward value. We then assessed the effect of HADS depression score on change in reaction time within the Huntington’s disease group (controls were excluded) using the same model comparison process. Potential confounding variables were assessed using the model comparison approach described for the regression models.

#### Data availability

Anonymized data are available on reasonable request.

## Results

### Study 1: effect of antidepressant class on depression in Huntington’s disease using propensity score analysis

#### Prescribing Patterns for Depression in Huntington’s disease

In the ENROLL-HD data set, 5486 (37.71%) participants (Table 2 and Supplementary Table 2) received at least one prescription for an antidepressant for low mood in the course of the study. There were a total of 9968 antidepressant prescriptions. Table 2 shows that the majority of antidepressant prescriptions (61.99%) were for selective serotonin reuptake inhibitors (SSRIs), followed by SNRIs; 15.29% and TeCA; 11.38%. Citalopram, sertraline and venlafaxine were the most frequently prescribed individual medications. The treated cohort had a high frequency of both psychiatric comorbidity (comorbid psychiatric symptoms, previous mental health history or suicide attempt) and concurrent prescription of benzodiazepine or antipsychotic drugs (Table 1). Moclobemide was the only MAOI prescribed for low mood and comprised a small minority of total prescriptions (0.20%); hence it was included in the unique class for the purposes of the analysis.

**Table 2:** Frequency of use by drug class

Drug class	Frequency	Percentage
SSRI	6179	61.99
SNRI	1524	15.29
TeCA	1134	11.38
NDRI	441	4.42
TCA	435	4.36
Phenylpiperazine	175	1.76
Unique	60	0.60
MAOI	20	0.20

Frequency includes total number of prescriptions—including dose escalations.

SSRI, selective serotonin reuptake inhibitor; TeCA, tetracyclic antidepressant; SNRI, serotonin–noradrenaline reuptake inhibitor; TCA, tricyclic antidepressant; NDRI, noradrenaline–dopamine reuptake inhibitor.

#### Efficacy of different antidepressant classes for depression in Huntington’s disease

**Figure 1:**
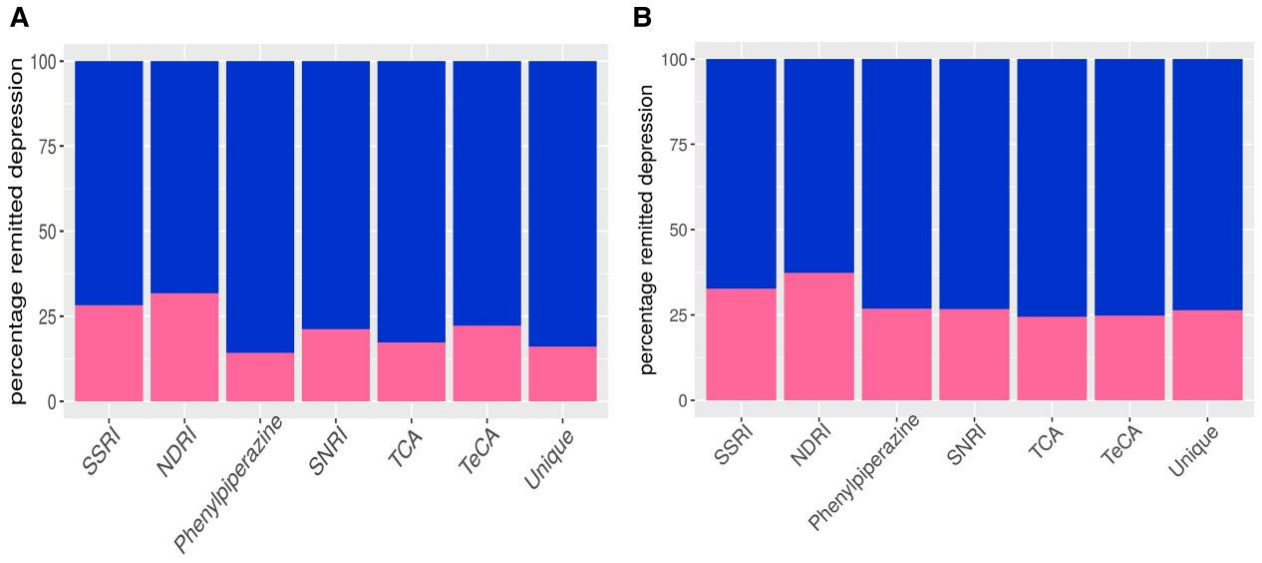
**Treatment response to antidepressants.** Percentage of population with depression at follow-up following treatment with different classes of antidepressant; ANOVAs comparing models with, and without antidepressant (drug class) variable are reported (N = 5486 for both). (**A**) At first follow-up, F = 5.062 df = 6,2440 *P* = 3.32 × 10–5; (**B**) at all follow-ups, F = 4.63, df = 6,12555, *P* = 0.00011. Outcome: dark, depressed; light, non-depressed.

At first follow-up, treatment with SSRIs or an (Fig. 1A and B, Tables 3–6, Supplementary Tables 3–6) NDRI resulted in the highest frequency of remission from depression (28.02% and 32.39% respectively). SNRIs and TeCAs had intermediate efficacy with remission in 21.23% and 22.26% of individuals, whilst remission of depression was lowest in the groups receiving phenypiperazines (14.75%), TCAs; 17.59% or agents with a unique mechanism of action (unique; 15.09%). Across all subsequent follow-ups, remission from depression improved in all antidepressant classes, but SSRIs (remission in 32.70%) and NDRIs (remission in 37.31%) remained the most effective treatments.

**Table 3:** ATE analysis of drug class; outcome: depression at first follow-up

	Estimate	P-value
**SSRI as reference treatment**		
(Intercept)	0.72	<2 × 10^−16^
NDRI	−0.085	0.21
Phenylpiperazine	0.13	0.0066
SNRI	0.051	0.056
TCA	0.077	0.40
TeCA	0.017	0.67
Unique	0.14	0.056
**SSRI as reference treatment, doubly robust estimation**		
(Intercept)	0.61	1.62 × 10^−6^
NDRI	−0.060	0.42
Phenylpiperazine	0.10	0.059
SNRI	0.058	0.048
TCA	0.15	0.14
TeCA	0.0076	0.86
Unique	0.22	0.00028
Age	0.0016	0.33
Sex (Male)	−0.0082	0.85
Disease progression score	−0.0058	0.27
SSRI risk factors	−0.0059	0.33
Comorbidities	0.0042	0.93
Number of antidepressants	0.020	0.00067
Sedative	0.044	0.45
Fluoxetine equivalent dose (mg)	−2.85 × 10^−6^	0.84

ATE, actual treatment effect, SSRI, selective serotonin reuptake inhibitor; NDRI, norepinephrine–dopamine reuptake inhibitor, SNRI, serotonin–norepinephrine reuptake inhibitor; TCA, tricyclic antidepressant; TeCA, tetracyclic antidepressants.

**Table 4:** ATE analysis of drug class, outcome; depression at all follow-ups

	Estimate	P-value
**SSRI as reference treatment**		
(Intercept)	0.68	< 2 × 10^−16^
NDRI	−0.080	0.011
Phenylpiperazine	0.063	0.022
SNRI	0.031	0.013
TCA	0.088	0.027
TeCA	0.081	1.22 × 10^−6^
Unique	0.084	0.029
**SSRI as reference treatment, doubly robust estimation**		
(Intercept)	0.62	< 2 × 10^−16^
NDRI	−0.061	0.092
Phenylpiperazine	−0.0040	0.90
SNRI	0.045	0.00069
TCA	0.060	0.21
TeCA	0.026	0.16
Unique	0.057	0.17
Age	-0.0045	0.64
Sex (Male)	0.00099	0.96
Disease progression score	−0.0060	0.021
SSRI Risk Factors	−0.057	0.049
Comorbidities	0.026	0.27
Number of antidepressants	0.023	< 2 × 10^−16^
Sedative	0.095	0.0017
Fluoxetine equivalent dose (mg)	1.28 × 10^−5^	0.029

ATE, actual treatment effect, SSRI, selective serotonin reuptake inhibitor; NDRI, norepinephrine–dopamine reuptake inhibitor; SNRI, serotonin–Norepinephrine reuptake inhibitor; TCA, tricyclic antidepressant; TARC, tetracyclic and related antidepressants.

**Table 5:** ATE analysis of drug class; outcome: depression at first follow-up

	Estimate	P-value
**SNRI as reference treatment**		
(Intercept)	0.77	<2 × 10^−16^
NDRI	−0.14	0.052
Phenylpiperazine	0.082	0.12
SSRI	−0.051	0.056
TCA	0.025	0.78
TeCA	−0.034	0.44
Unique	0.088	0.24
**SNRI as reference treatment, doubly robust estimation**		
(Intercept)	0.67	4.11 × 10^−7^
NDRI	−0.12	0.13
Phenylpiperazine	0.047	0.42
SSRI	−0.58	0.048
TCA	0.089	0.37
TeCA	−0.050	0.29
Unique	0.16	0.012
Age	0.0016	0.33
Sex (Male)	−0.0082	0.85
Disease progression score	−0.0058	0.27
SSRI risk factors	−0.059	0.33
Comorbidities	0.0042	0.93
Number of antidepressants	0.020	0.00067
Sedative	20.044	0.45
Fluoxetine equivalent dose (mg)	−2.86 × 10^−6^	0.84

ATE, actual treatment effect; SSRI, selective serotonin reuptake inhibitor; NDRI, norepinephrine–dopamine reuptake inhibitor; SNRI, serotonin–norepinephrine reuptake inhibitor; TCA, tricyclic antidepressant; TARC, tetracyclic and related antidepressants.

**Table 6:** ATE analysis of drug class; outcome: depression at all follow-ups

	Estimate	P-value
**SNRI as reference treatment**		
(Intercept)	0.71	<2 × 10^−16^
NDRI	−0.11	0.00082
Phenylpiperazine	0.032	0.27
SSRI	−0.031	0.013
TCA	0.058	0.16
TeCA	0.050	0.0091
Unique	0.054	0.18
**SNRI as reference treatment, doubly robust estimation**		
(Intercept)	0.66	<2 × 10^−16^
NDRI	−0.111	0.0045
Phenylpiperazine	−0.049	0.14
SSRI	−0.045	0.00069
TCA	0.015	0.75
TeCA	−0.019	0.38
Unique	0.012	0.77
Age	−0.00045	0.64
Sex (Male)	0.00099	0.96
Disease progression score	−0.0060	0.021
SSRI risk factors	−0.0057	0.049
Comorbidities	0.026	0.27
Number of antidepressants	0.023	<2 × 10^−16^
Sedative	0.095	0.0017
Fluoxetine equivalent dose (mg)	1.28 × 10^−5^	0.029

ATE, actual treatment effect, SSRI, selective serotonin reuptake inhibitor, NDRI, norepinephrine–dopamine reuptake inhibitor; SNRI, serotonin–norepinephrine reuptake inhibitor; TCA, tricyclic antidepressant; TARC, tetracyclic and related antidepressants.

An initial propensity score analysis using SSRIs as a comparator (Table 3 and 4) showed that treatment with phenylpiperazines, SNRIs, or unique agents (at trend level) was associated with worse outcome at first follow-up compared with SSRIs. The doubly robust estimation model showed that treatment with SNRIs, or Unique agents was associated with inferior remission rate at first follow-up compared with SSRI treatment. A propensity score analysis of depression at all follow-ups (secondary outcome measure), and SSRI treatment as comparator showed superiority in remission from depression with an NDRI compared with an SSRI, whilst all other drug classes proved inferior to SSRIs. The doubly robust model showed the superiority of an NDRI to SSRIs in depression remission across all follow-ups persisted at trend level only, whilst SNRIs remained inferior to SSRIs.

A propensity score analysis for the primary outcome measure (depression at first follow-up) with SNRIs as the comparator treatment group (Table 5 and 6) suggested superiority of SSRI or NDRI treatment over SNRI at remission from depression at first follow-up but only at trend level. Doubly robust estimation confirmed that SSRIs but not NDRIs outperformed SNRIs at first follow-up. Across all follow-ups, remission from depression was higher with SSRIs or an NDRI, compared to SNRIs, whilst TeCAs were inferior to SNRIs. The model with doubly robust estimation, supported the superiority of SSRIs and the NDRI classes over SNRIs, with a larger effect size for NDRIs. ANOVAs confirmed the importance of antidepressant class on both the primary (F = 5.092, *P* = 3.32 × 10^−5^) and secondary outcome (F = 4.63, *P* = 0.00011).

Depression and apathy scores are strongly positively correlated in the ENROLL-HD data set (*P* < 2 × 10^−16^). ANOVAs showed that inclusion of all apathy scores following antidepressant prescription improved both depression outcome models (primary: F = 3.49, *P* = 0.0020; secondary: F = 3.23, *P* = 0.0036). However, this did not influence the results: SSRIs remained superior to Unique agents and SNRIs, on both the primary and secondary depression outcomes. ANOVAs of the effect of antidepressant class on PBAs apathy score only proved significant at first follow-up (F = 3.33, *P* = 0.0029), and showed superiority of SSRIs to unique agents but no other antidepressants (Supplementary Tables 3–6).

This analysis is consistent with improved remission from depressed mood in Huntington’s disease with an NDRI or SSRIs compared with other antidepressants, with a limited effect of antidepressants on apathy.

### Study 2: cognitive mechanisms of depression in Huntington’s disease

#### Demographics and Psychopathology Questionnaires

The Huntington’s disease participants had lower (Table 1) premorbid IQ scores, were taking higher doses of antipsychotic (anti-dopaminergic) medication and antidepressant medication, and had worse scores on formal motor examination. The burden of psychopathology was also higher in the Huntington’s disease group, who had higher scores than controls on the HADS depression score, AES, PBAs apathy, perseveration, disorientation, irritability and aggression subscales.

#### Reward reaction time task: *motivational anhedonia*

**Figure 2:**
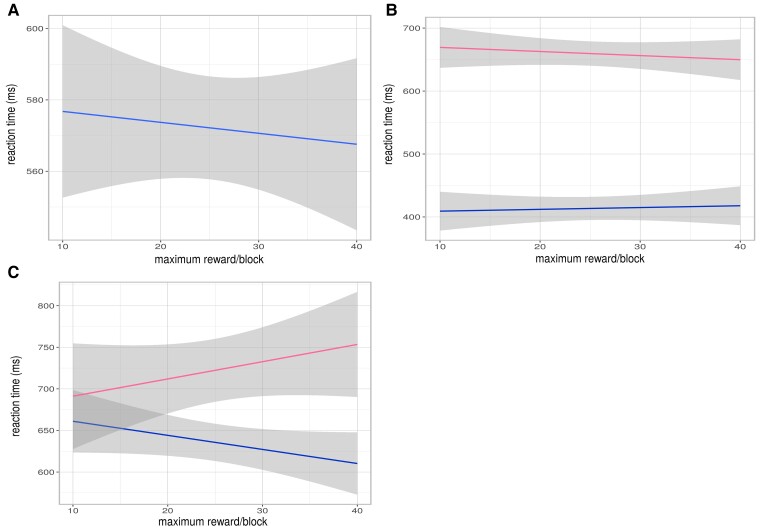
**Effort for reward—group comparisons and association with depression score.** (**A**). Whole group (Huntington’s disease cases and controls) effect of reward value on reaction time. (**B**). Whole group: effect of case status on change in reaction time with reward value (AIC weight 0.62, N = 74). Pink: Huntington’s disease; blue: control. (**C**). Huntington’s disease cases: effect of HADS depression score on change in reaction time with reward value (AIC weight 1.0, N = 46). Pink: HADS high > 7; blue: HADS low < 8.

As we have previously reported,^64^ mean reaction time (Fig. 2A, B and C, and Supplementary Tables 7–10) was faster for higher reward value trials in the whole group (estimate = −0.0027, *P* = 3.63 × 10^−^5), whilst Huntington’s disease cases had slower reaction times than controls (best model included case status: weight = 0.62, estimate = 0.22, *P* = 0.032), even when corrected for TMS, which was also strongly associated with slower reaction times (*P* = 4.98 × 10^−5^) (Fig. 1B). To assess the effect of low mood on reward related reaction time in Huntington’s disease, we compared models including the HADS depression score in the Huntington’s disease group. The best model (weight = 1.0), included the HADS depression score as an interaction with reward value. Higher HADS depression score was associated with slower reaction time overall (estimate = 0.045, *P* = 0.014) and there was a significant interaction with reward value: higher HADS depression scores were associated with slower reaction time as reward value increased (estimate = 0.00059, *P* = 1.38 × 10^−8^)(Fig. 1C). In this model TMS was also associated with reaction time impairment (estimate = 0.0099, *P* = 0.00015). Likelihood ratio tests of potential confounding variables (demographic variables and psychopathology scores from Table 1) did not improve on any of the models. Even accounting for motor disability, Huntington’s disease cases have blunted effort for reward compared with controls, and depression in Huntington’s disease leads to reduced effort at higher reward value.

#### BISBAS Reward Score: *reward responsiveness*

The model comparing (Supplementary Fig. 1A and B, Supplementary Tables 11 and 12) the effect of case status on BISBAS reward score did not show a significant effect of having Huntington’s disease (*P* = 0.28) and was not improved by any potential confounder. BISBAS reward score was not associated with HADS depression score in the Huntington’s disease group before or after inclusion of significant confounders (*P* = 0.57 and 0.54, respectively).

#### PSLT: instrumental learning from reward and *punishment*

Huntington’s disease participants (Supplementary Fig. 1C and D, Supplementary Tables 13–20) took longer to reach criterion (estimate = 0.45, *P* = 0.00079) and had lower total learning score (estimate = -12.54, *P* = 0.0092; sum of punishment and reward learning scores) compared with controls. Huntington’s disease participants did not differ from controls on reward learning (*P* = 0.56) but had lower scores on punishment learning (estimate = −0.29, *P* = 0.00072), an effect that was not maintained when confounders (TMS, olanzapine equivalent dose) were added to the model. Reward learning scores were not associated with HADS depression score in the Huntington’s disease group (*P* = 0.49). The interaction between reward learning and punishment learning, also did not predict HADS depression score. In contrast, higher AES score in the Huntington’s disease group was associated with lower punishment learning score (estimate = −0.0079, *P* = 0.036), although this did not survive inclusion of confounders (TMS, olanzapine equivalent dose) in the model. The interaction between punishment learning and reward learning score (higher reward learning compared to punishment learning) was also associated with higher AES score (estimate = 0.00036, *P* = 0.030), although significance was not maintained after inclusion of confounders (TMS, olanzapine equivalent dose). We found no evidence to support an association between either reward learning or punishment learning and depression in Huntington’s disease, but in keeping with our previous findings,^64^ our results suggest an association between impaired punishment learning and apathy.

#### Race task: *response to negative outcome*

##### Post-task predicted performance (absolute and change from baseline)

The Huntington’s disease group had lower estimates (Supplementary Fig. 2, Supplementary Tables 21–26) compared with controls for performance following negative feedback (estimate = −0.27, *P* = 5.13 × 10^−11^), an effect that was strengthened by the addition of potential confounders (estimate = −0.58, *P* < 2 × 10^−16^). However, in the Huntington’s disease group, post-task estimate was not associated with HADS depression score, before or after inclusion of significant confounders (*P* = 0.89 and 0.77, respectively). When the baseline prediction of performance was accounted for (post-task minus pre-task prediction), no differences were seen between Huntington’s disease cases and controls before or after inclusion of confounders (*P* = 0.22 and 0.40 respectively). Within the Huntington’s disease group, the change in predicted performance from baseline was not associated with HADS depression score before or after inclusion of confounders (*P* = 0.16 and *P* = 0.40, respectively). No association was found between AES score and either measure. These findings suggest that Huntington’s disease cases estimate their performance more negatively than controls overall, but this is not selectively affected by negative feedback, and is not associated with depression in Huntington’s disease.

## Discussion

To our knowledge, this is the first study to demonstrate objective evidence of treatment effectiveness for depression in Huntington’s disease to inform clinical decision-making and is supported by the cognitive mechanism data from Study 2. Study 1 demonstrates that unlike MDD, the most effective treatments for depression in Huntington’s disease are drawn from SSRI and NDRI classes, whilst Study 2 shows that depression in Huntington’s disease is associated with motivational anhedonia but not with alterations in learning from reward, reward responsiveness or hypersensitivity to negative outcomes. Finally, we found a dissociation between apathy and depression in Huntington’s disease; depression, but not apathy, was associated with motivational anhedonia: a core dopaminergic process.^118–120^

Here we have shown that SSRI and NDRI antidepressants were superior to other antidepressant classes in treating depression in Huntington’s disease: our analysis demonstrated they were the only antidepressant classes to show superiority to SNRIs. Furthermore our data suggest possible superiority of Bupropion over SSRIs, with a larger effect size for depression remission compared to SSRIs in the propensity score analysis using SNRIs as the comparator, and trend level superiority at treatment efficacy in our secondary outcome when directly compared with SSRIs. This starkly contrasts with MDD, where a large meta-analysis^54^ demonstrated superior efficacy of TeCAs, TCAs and SNRIs over the majority of SSRI and NDRI agents. Determining why some antidepressants are more effective at treating MDD is difficult owing to the diversity of neurobiological mechanisms linked with the condition.^121,122^ Whilst evidence is limited, no differences in efficacy between antidepressant classes have been seen in reducing inflammatory induced depression,^123–127^ or efficacy at normalizing the hypothalamic–pituitary axis in MDD (reviewed in Nandam *et al.*^128^). Deficits in hippocampal neurogenesis have been found in both MDD and Huntington’s disease.^129,130^ Intriguingly, both in humans^131–133^ and in animals,^134^ SSRIs have shown superiority over other antidepressant classes at improving hippocampal neurogenesis, whilst SSRIs have also improved hippocampal neurogenesis in animal models of Huntington’s disease.^135,136^ SSRIs and NDRIs rescued depression-like phenotypes in animal models,^137,138^ and showed effects on cognition and motor phenotypes of Huntington’s disease animal models^139,140^ (though not in human subjects^28^). Finally, damage to the serotonergic dorsal raphe nucleii has been associated with depression in Huntington’s disease^141^: hence increasing serotonergic tone may be particularly useful in ameliorating depression in Huntington’s disease.

SSRIs and SNRIs also differ in their effects on cognitive processes underlying MDD. The RDoC emphasise the contributions of the Loss and Reward domains to MDD.^38,92^ Whilst limited evidence suggests equivalent effect of antidepressant classes on behavioural changes in the loss domain,^142,143^ reward related behaviours are differentially affected by antidepressant class. Noradrenergic neurons originating in the locus coeruleus encode effort required for an action,^144^ and increasing noradrenergic tone does not affect, or even reduces effort for reward.^57,59,145,146^ The role of noradrenaline and effort for reward is complex: locus coeruleus activity is closely linked with effort required for an action,^144^ and acute reduction in noradrenergic tone reduces effort for reward.^147^ However, acute and chronic administration of noradrenaline reuptake inhibitors reduces effort for reward.^57,58,145,148^ Correspondingly SNRIs are comparatively less effective at increasing effort for reward, than other antidepressants.^149^ In contrast, although SSRIs are associated with emotional blunting (consummatory anhedonia),^55,150–152^ and acutely bupropion and SSRIs have little effect on dopaminergic tone or effort for reward,^58,153–155^ more recent data suggests that chronic use improves motivational anhedonia. Animal studies show that bupropion^58,155–158^ and serotonergic stimulation^159,160^ increase effort for reward, and reverse depressive-like behaviours in animal models of Huntington’s disease^137,138^; whilst increasing dopamine and serotonergic tone concurrently has a synergistic effect^159,161^ on effort for reward. Studies in human controls suggest that effort learning and effort for reward are increased with SSRIs^160,162–164^ and bupropion,^165–167^ whilst both SSRIs and bupropion have shown specific improvements in measures of anergia and motivational anhedonia in MDD.^168–171^

The behavioural study findings support the propensity score analysis in pointing to a key role of the mesolimbic system. We found a major contribution of motivational anhedonia to depression in Huntington’s disease but no deficits in reward responsiveness, reward learning or heightened response to loss. Decision-making regarding effort costs and reward value is mediated by a distributed network including the anterior cingulate cortex, amygdala, ventral striatum, and orbito-frontal cortex.^35,36,94,172–174^ These areas are all affected by the neurodegeneration caused by Huntington’s disease.^175–179^ The anterior cingulate cortex has a major role in tracking effort costs,^172,173,180–182^ whilst increasing tone in the dopaminergic mesolimbic circuitry increases effort for reward.^145,183–186^ Damage to the anterior cingulate cortex is particularly associated with depression in Huntington’s disease, with evidence of selective interneuron loss in the anterior cingulate cortex mediating a ‘mood’ phenotype,^178,179^ and imaging parameters (reduced fractional anisotropy measured by diffusion tensor imaging) suggesting reduced integrity of the white matter underlying the anterior cingulate cortex, insula and cerebellum being linked with depression scores.^187,188^

Prior work in MDD has demonstrated an association with hyper-responsiveness to negative stimuli: on behavioural tasks,^50,189^ feedback-related negativity on EEG in response to task based punishment,^47,48^ and BOLD signal in the amygdala during negative feedback.^49^ However, we did not replicate this association with depression in Huntington’s disease. Deficits in response to negative stimuli are well documented in Huntington’s disease: impairments in learning from losses, and effort to avoid losses have been demonstrated in both pre-symptomatic and symptomatic patients,^61,64,190^ with an association shown between apathy in Huntington’s disease and deficits in this process.^64^ This association was replicated in the PSLT data: we found an association between impaired punishment learning and apathy. This may reflect a wider insensitivity to negative stimuli in Huntington’s disease, as patients also demonstrate deficits in negative emotion recognition,^63,191,192^ and altered decision-making relating to altruistic punishment and losses.^193–195^

The results from our behavioural study (Study 2) suggest a double dissociation between behavioural measures of depression and apathy in Huntington’s disease, showing motivational anhedonia underlies depression but not apathy; whilst apathy (but not depression) is associated with altered instrumental learning from negative stimuli, replicating our findings in prior work.^64^ In keeping with a previous RCT, and our behavioural data, the propensity score analyses found a limited effect of antidepressants on apathy outcomes. Previous reports in Parkinson’s disease,^120,196^ CADASIL^197^ and schizophrenia,^198,199^ have found deficits in effort for reward in association with apathy, but did not report the effect of depression despite higher scores in their patient cohorts. An association between a deficit in effort for reward and apathy has also been reported in Huntington’s disease.^200^ However, the majority of the Huntington’s disease patient group in this study were also on treatment for depression which was not accounted for in the analysis. The finding of a potential double dissociation on behavioural tasks would allow a mechanistic distinction between apathy and depression in Huntington’s disease and therefore has important implications for animal models; facilitating the development of translational tasks to test novel and existing interventions for apathy and depression in Huntington’s disease.

This work does have some limitations. Propensity scoring analyses are not yet established as being equivalent (or superior) to randomised controlled trials, which remain the gold standard in assessing treatment efficacy by eliminating unknown sample bias. This work on depression in Huntington’s disease presents separate, but complementary analyses which point to a central role for motivational anhedonia in the pathophysiology, and suggest superior efficacy of antidepressants primarily acting on motivational anhedonia. Our findings should not influence antidepressant choice at present, but argue for inclusion of NDRI and SSRI arms in a future randomised controlled trial. The race task has not been previously used but similar tasks have been used in MDD populations—with a clear demonstration of increased response to negative feedback seen in individuals with MDD.^47,49,50,106^ Our control group did include familial (gene negative) Huntington’s disease controls but also had control subjects drawn from the general population, which may have reduced our ability to control for psychosocial stresses (of being at risk for Huntington’s disease or caring for a family member with Huntington’s disease); future studies could recruit control subjects exclusively from family members to avoid this possibility. We used a sample of Huntington’s disease participants that included individuals with motor symptoms and those without. It is possible that mechanisms of depression in Huntington’s disease may change with disease stage, but our models did include motor score as a confounding variable to account for this problem. Finally, we used the BISBAS Reward scale as a measure of reward responsiveness (consummatory anhedonia), which is not the most commonly used instrument for this purpose, although it has previously been used to show dysfunction within a network critical to experiential pleasure.^201^

In summary, propensity score analysis of the Enroll-HD data set revealed that, in contrast to MDD, SSRI and NDRI antidepressant classes are superior to other major antidepressant classes at treating depressed mood in Huntington’s disease. This is supported by our prospective behavioural study which demonstrated that motivational anhedonia is a core process of depression in Huntington’s disease. Conversely, increased response to negative feedback does not contribute to depression in Huntington’s disease. Finally apathy and depression in Huntington’s disease are dissociable on a task of effort and reward. The importance for future research and clinical practice is that the effectiveness of medications for depression in neurodegenerative disease is not necessarily the same as in MDD. This knowledge is important both for the refinement of treatment approaches and for improved mechanistic research in animal models, and highlights the need for a randomised controlled trial to confirm our findings.

## Supplementary Material

fcac278_Supplementary_Data

## Abbreviations

AES =: Apathy Evaluation Scale
ATE =: average treatment effect
ATT =: average treatment effect among the treated
BISBAS =: Behavioural Inhibition Scale Behavioural Activation Scale
CADASIL =: Cerebral Autosomal Dominant Arteriopathy with Subcortical Infarcts and Leukoencephalopathy
GLM =: general linear model
GLMM =: generalised linear mixed models
HADS =: Hospital Anxiety and Depression Scale
MAOIs =: monoamine oxidase inhibitors
MDD =: major depressive disorder
NDRI =: noradrenaline–dopamine reuptake inhibitor
PBAs =: Problem Behaviours Assessment (short form)
PSLT =: Probabilistic Selection Learning Task
RCT =: randomised controlled trial
RDoC =: Research Domain Criteria
RRTT =: Reward Reaction Time Task
SNRIs =: serotonin–norepinephrine reuptake inhibitors
TCA =: tricyclic antidepressants
TeCAs =: tetracyclic antidepressants
UHDRS-TFC =: Unified Huntington’s disease Rating Scale Total Functional Capacity
UHDRS-TMS =: Unified Huntington’s disease Rating Scale Total Motor Score

## References

[1] Reijnders JSAM , Ehrt U, Weber WEJ, et al A systematic review of prevalence studies of depression in Parkinson’s disease. Mov Disord Off J Mov Disord Soc. 2008;23(2):183–189. quiz 313. 10.1002/mds.21803 17987654

[2] Rickards H . Depression in neurological disorders: Parkinson’s disease, multiple sclerosis, and stroke. J Neurol Neurosurg Psychiatry. 2005;76(suppl 1):i48–i52. 15718222 10.1136/jnnp.2004.060426 PMC1765679

[3] Worku DK , Yifru YM, Postels DG, Gashe FE. Prevalence of depression in Parkinson’s disease patients in Ethiopia. J Clin Mov Disord. 2014;1(1):10. 26788336 10.1186/s40734-014-0010-3 PMC4711030

[4] Zubenko GS , Zubenko WN, McPherson S, et al A collaborative study of the emergence and clinical features of the Major depressive syndrome of Alzheimer’s disease. Am J Psychiatry. 2003;160(5):857–866. 12727688 10.1176/appi.ajp.160.5.857

[5] Lyketsos CG , Steinberg M, Tschanz JT, et al Mental and behavioral disturbances in dementia: Findings from the cache county study on memory in aging. Am J Psychiatry. 2000;157(5):708–714. 10784462 10.1176/appi.ajp.157.5.708

[6] van Duijn E , Kingma EM, van der Mast RC. Psychopathology in verified huntington’s disease gene carriers. J Neuropsychiatry Clin Neurosci. 2007;19(4):441–448. 18070848 10.1176/jnp.2007.19.4.441

[7] van Duijn E , Craufurd D, Hubers AAM, et al Neuropsychiatric symptoms in a European huntington’s disease cohort (REGISTRY). J Neurol Neurosurg Psychiatry. 2014;85(12):1411–1418. 24828898 10.1136/jnnp-2013-307343

[8] Zhang L-Y , Cao B, Zou Y-T, et al Depression and anxiety in multiple system atrophy. Acta Neurol Scand. 2018;137(1):33–37. 28748633 10.1111/ane.12804

[9] Chakrabarty T , Sepehry AA, Jacova C, Hsiung G-YR. The prevalence of depressive symptoms in frontotemporal dementia: A meta-analysis. Dement Geriatr Cogn Disord. 2015;39(5–6):257–271. 25662033 10.1159/000369882

[10] Wicks P , Abrahams S, Masi D, et al Prevalence of depression in a 12-month consecutive sample of patients with ALS. Eur J Neurol. 2007;14(9):993–1001. 17718691 10.1111/j.1468-1331.2007.01843.x

[11] Smith DJ , Nicholl BI, Cullen B, et al Prevalence and characteristics of probable Major depression and bipolar disorder within UK biobank: Cross-sectional study of 172,751 participants. PLoS One. 2013;8(11):e75362. 24282498 10.1371/journal.pone.0075362 PMC3839907

[12] Wilhelm K , Mitchell P, Slade T, et al Prevalence and correlates of DSM-IV major depression in an Australian national survey. J Affect Disord. 2003;75(2):155–162. 12798255 10.1016/s0165-0327(02)00040-x

[13] MacDonald ME , Ambrose CM, Duyao MP, et al A novel gene containing a trinucleotide repeat that is expanded and unstable on huntington’s disease chromosomes. Cell. 1993;72(6):971–983. 8458085 10.1016/0092-8674(93)90585-e

[14] Tabrizi SJ , Scahill RI, Owen G, et al Predictors of phenotypic progression and disease onset in premanifest and early-stage huntington’s disease in the TRACK-HD study: Analysis of 36-month observational data. Lancet Neurol. 2013;12(7):637–649. 23664844 10.1016/S1474-4422(13)70088-7

[15] Stout JC , Paulsen JS, Queller S, et al Neurocognitive signs in prodromal huntington disease. Neuropsychology. 2011;25(1):1–14. 20919768 10.1037/a0020937 PMC3017660

[16] Duff K , Paulsen JS, Beglinger LJ, et al Psychiatric symptoms in huntington’s disease before diagnosis: The predict-HD study. Biol Psychiatry. 2007;62(12):1341–1346. 17481592 10.1016/j.biopsych.2006.11.034

[17] Julien CL , Thompson JC, Wild S, et al Psychiatric disorders in preclinical huntington’s disease. J Neurol Neurosurg Psychiatry. 2007;78(9):939–943. 17178819 10.1136/jnnp.2006.103309 PMC2117854

[18] Craufurd D , Thompson JC, Snowden JS. Behavioral changes in huntington disease. Neuropsychiatry Neuropsychol Behav Neurol. 2001;14(4):219–226. 11725215

[19] Ready RE , Mathews M, Leserman A, Paulsen JS. Patient and caregiver quality of life in huntington’s disease. Mov Disord Off J Mov Disord Soc. 2008;23(5):721–726. 10.1002/mds.21920 PMC3789516 18175350

[20] Ho AK , Gilbert AS, Mason SL, et al Health-related quality of life in huntington’s disease: Which factors matter most? Mov Disord Off J Mov Disord Soc. 2009;24(4):574–578. 10.1002/mds.22412 19097181

[21] Hamilton JM , Salmon DP, Corey-Bloom J, et al Behavioural abnormalities contribute to functional decline in huntington’s disease. J Neurol Neurosurg Psychiatry. 2003;74(1):120–122. 12486282 10.1136/jnnp.74.1.120 PMC1738208

[22] Wheelock VL , Tempkin T, Marder K, et al Predictors of nursing home placement in huntington disease. Neurology. 2003;60(6):998–1001. 12654967 10.1212/01.wnl.0000052992.58107.67

[23] Banaszkiewicz K , Sitek EJ, Rudzińska M, et al Huntington’s disease from the patient, caregiver and physician’s perspectives: Three sides of the same coin? J Neural Transm. 2012;119(11):1361–1365. 22398875 10.1007/s00702-012-0787-x PMC3477481

[24] Read J , Jones R, Owen G, et al Quality of life in huntington’s disease: A comparative study investigating the impact for those with pre-manifest and early manifest disease, and their partners. J Huntingt Dis. 2013;2(2):159–175. 10.3233/JHD-130051 25063513

[25] Killoran A , Biglan KM, Jankovic J, et al Characterization of the huntington intermediate CAG repeat expansion phenotype in PHAROS. Neurology. 2013;80(22):2022–2027. 23624566 10.1212/WNL.0b013e318294b304 PMC3716408

[26] Pang TYC , Du X, Zajac MS, et al Altered serotonin receptor expression is associated with depression-related behavior in the R6/1 transgenic mouse model of huntington’s disease. Hum Mol Genet. 2009;18(4):753–766. 19008301 10.1093/hmg/ddn385

[27] Pouladi MA , Graham RK, Karasinska JM, et al Prevention of depressive behaviour in the YAC128 mouse model of huntington disease by mutation at residue 586 of huntingtin. Brain J Neurol. 2009;132(Pt 4):919–932. 10.1093/brain/awp006 19224899

[28] Beglinger LJ , Adams WH, Langbehn D, et al Results of the citalopram to enhance cognition in huntington disease trial. Mov Disord Off J Mov Disord Soc. 2014;29(0 3):401–405. 10.1002/mds.25750 PMC3960314 24375941

[29] Gelderblom H , Wüstenberg T, McLean T, et al Bupropion for the treatment of apathy in huntington’s disease: A multicenter, randomised, double-blind, placebo-controlled, prospective crossover trial. PLoS One. 2017;12(3):e0173872. 28323838 10.1371/journal.pone.0173872 PMC5360242

[30] Holl AK , Wilkinson L, Painold A, et al Combating depression in huntington’s disease: Effective antidepressive treatment with venlafaxine XR. Int Clin Psychopharmacol. 2010;25(1):46–50. 19996754 10.1097/YIC.0b013e3283348018

[31] Ford MF . Treatment of depression in huntington’s disease with monoamine oxidase inhibitors. Br J Psychiatry. 1986;149(5):654–656. 2949793 10.1192/bjp.149.5.654

[32] Bonelli RM . Mirtazapine in suicidal huntington’s disease. Ann Pharmacother. 2003;37(3):452. 10.1345/aph.1C352 12639181

[33] Patel SV , Tariot PN, Asnis J. L-Deprenyl augmentation of fluoxetine in a patient with huntington’s disease. Ann Clin Psychiatry Off J Am Acad Clin Psychiatr. 1996;8(1):23–26. 10.3109/10401239609149087 8743645

[34] Beck AT . The evolution of the cognitive model of depression and its neurobiological correlates. Am J Psychiatry. 2008;165(8):969–977. 18628348 10.1176/appi.ajp.2008.08050721

[35] Der-Avakian A , Markou A. The neurobiology of anhedonia and other reward-related deficits. Trends Neurosci. 2012;35(1):68–77. 22177980 10.1016/j.tins.2011.11.005 PMC3253139

[36] Höflich A , Michenthaler P, Kasper S, Lanzenberger R. Circuit mechanisms of reward, anhedonia, and depression. Int J Neuropsychopharmacol. 2019;22(2):105–118. 30239748 10.1093/ijnp/pyy081 PMC6368373

[37] Koster EHW , De Lissnyder E, Derakshan N, De Raedt R. Understanding depressive rumination from a cognitive science perspective: The impaired disengagement hypothesis. Clin Psychol Rev. 2011;31(1):138–145. 20817334 10.1016/j.cpr.2010.08.005

[38] Insel T , Cuthbert B, Garvey M, et al Research domain criteria (RDoC): Toward a new classification framework for research on mental disorders. Am J Psychiatry. 2010;167(7):748–751. 20595427 10.1176/appi.ajp.2010.09091379

[39] Pizzagalli DA , Jahn AL, O’Shea JP. Toward an objective characterization of an anhedonic phenotype: A signal-detection approach. Biol Psychiatry. 2005;57(4):319–327. 15705346 10.1016/j.biopsych.2004.11.026 PMC2447922

[40] Pizzagalli DA , Iosifescu D, Hallett LA, et al Reduced hedonic capacity in major depressive disorder: Evidence from a probabilistic reward task. J Psychiatr Res. 2008;43(1):76–87. 18433774 10.1016/j.jpsychires.2008.03.001 PMC2637997

[41] Pizzagalli DA , Holmes AJ, Dillon DG, et al Reduced caudate and nucleus accumbens response to rewards in unmedicated individuals with major depressive disorder. Am J Psychiatry. 2009;166(6):702–710. 19411368 10.1176/appi.ajp.2008.08081201 PMC2735451

[42] Treadway MT , Bossaller NA, Shelton RC, Zald DH. Effort-based decision-making in major depressive disorder: A translational model of motivational anhedonia. J Abnorm Psychol. 2012;121(3):553–558. 22775583 10.1037/a0028813 PMC3730492

[43] Geugies H , Mocking RJT, Figueroa CA, et al Impaired reward-related learning signals in remitted unmedicated patients with recurrent depression. Brain. 2019;142(8):2510–2522. 31280309 10.1093/brain/awz167 PMC6734943

[44] Snaith RP , Hamilton M, Morley S, et al A scale for the assessment of hedonic tone the snaith-Hamilton pleasure scale. Br J Psychiatry J Ment Sci. 1995;167(1):99–103. 10.1192/bjp.167.1.99 7551619

[45] Hershenberg R , Satterthwaite TD, Daldal A, et al Diminished effort on a progressive ratio task in both unipolar and bipolar depression. J Affect Disord. 2016;196:97–100. 26919058 10.1016/j.jad.2016.02.003 PMC4808384

[46] Sherdell L , Waugh CE, Gotlib IH. Anticipatory pleasure predicts motivation for reward in major depression. J Abnorm Psychol. 2012;121(1):51–60. 21842963 10.1037/a0024945 PMC3335300

[47] Santesso DL , Steele KT, Bogdan R, et al Enhanced negative feedback responses in remitted depression. Neuroreport. 2008;19(10):1045–1048. 18580576 10.1097/WNR.0b013e3283036e73 PMC3034237

[48] Webb CA , Auerbach RP, Bondy E, et al Abnormal neural responses to feedback in depressed adolescents. J Abnorm Psychol. 2017;126(1):19–31. 27935729 10.1037/abn0000228 PMC5215965

[49] Tavares JV T , Clark L, Furey ML, et al Neural basis of abnormal response to negative feedback in unmedicated mood disorders. NeuroImage. 2008;42(3):1118–1126. 18586109 10.1016/j.neuroimage.2008.05.049 PMC2745889

[50] Elliott R , Sahakian BJ, Herrod JJ, et al Abnormal response to negative feedback in unipolar depression: Evidence for a diagnosis specific impairment. J Neurol Neurosurg Psychiatry. 1997;63(1):74–82. 9221971 10.1136/jnnp.63.1.74 PMC2169625

[51] Hayward G , Goodwin GM, Cowen PJ, Harmer CJ. Low-dose tryptophan depletion in recovered depressed patients induces changes in cognitive processing without depressive symptoms. Biol Psychiatry. 2005;57(5):517–524. 15737667 10.1016/j.biopsych.2004.11.016

[52] Roiser JP , Levy J, Fromm SJ, et al The effect of acute tryptophan depletion on the neural correlates of emotional processing in healthy volunteers. Neuropsychopharmacology. 2008;33(8):1992–2006. 17882232 10.1038/sj.npp.1301581 PMC2645340

[53] Eshel N , Roiser JP. Reward and punishment processing in depression. Biol Psychiatry. 2010;68(2):118–124. 20303067 10.1016/j.biopsych.2010.01.027

[54] Cipriani A , Furukawa TA, Salanti G, et al Comparative efficacy and acceptability of 21 antidepressant drugs for the acute treatment of adults with major depressive disorder: A systematic review and network meta-analysis. The Lancet. 2018;391(10128):1357–1366. 10.1016/S0140-6736(17)32802-7 PMC5889788 29477251

[55] Price J , Cole V, Goodwin GM. Emotional side-effects of selective serotonin reuptake inhibitors: Qualitative study. Br J Psychiatry. 2009;195(3):211–217. 19721109 10.1192/bjp.bp.108.051110

[56] Goodwin GM , Price J, De Bodinat C, Laredo J. Emotional blunting with antidepressant treatments: A survey among depressed patients. J Affect Disord. 2017;221:31–35. 28628765 10.1016/j.jad.2017.05.048

[57] Achterberg EJM , van Kerkhof LWM, Servadio M, et al Contrasting roles of dopamine and noradrenaline in the motivational properties of social play behavior in rats. Neuropsychopharmacology. 2016;41(3):858–868. 26174597 10.1038/npp.2015.212 PMC4707831

[58] Yohn SE , Collins SL, Contreras-Mora HM, et al Not all antidepressants are created equal: Differential effects of monoamine uptake inhibitors on effort-related choice behavior. Neuropsychopharmacol Off Publ Am Coll Neuropsychopharmacol. 2016;41(3):686–694. 10.1038/npp.2015.188 PMC4707815 26105139

[59] España RA , Schmeichel BE, Berridge CW. Norepinephrine at the nexus of arousal, motivation and relapse. Brain Res. 2016;1641(Pt B):207–216. 26773688 10.1016/j.brainres.2016.01.002 PMC4879075

[60] Yohn SE , Errante EE, Rosenbloom-Snow A, et al Blockade of uptake for dopamine, but not norepinephrine or 5-HT, increases selection of high effort instrumental activity: Implications for treatment of effort-related motivational symptoms in psychopathology. Neuropharmacology. 2016;109:270–280. 27329556 10.1016/j.neuropharm.2016.06.018

[61] Palminteri S , Justo D, Jauffret C, et al Critical roles for anterior insula and dorsal striatum in punishment-based avoidance learning. Neuron. 2012;76(5):998–1009. 23217747 10.1016/j.neuron.2012.10.017

[62] Sprengelmeyer R , Young AW, Calder AJ, et al Loss of disgust. Perception of faces and emotions in huntington’s disease. Brain J Neurol. 1996;119(Pt 5):1647–1665. 10.1093/brain/119.5.1647 8931587

[63] Johnson SA , Stout JC, Solomon AC, et al Beyond disgust: Impaired recognition of negative emotions prior to diagnosis in huntington’s disease. Brain J Neurol. 2007;130(Pt 7):1732–1744. 10.1093/brain/awm107 17584778

[64] McLauchlan D , Lancaster T, Craufurd D, et al Insensitivity to loss predicts apathy in huntington’s disease. Mov Disord. 2019. 10.1002/mds.27787 31361357

[65] Rickards H. Personal Communication. 2018.

[66] Dudas R , Malouf R, McCleery J, Dening T. Antidepressants for treating depression in dementia [internet]. Cochrane Database Syst Rev. 2018(8). [cited 2021 Oct 6] Available from: https://www.cochranelibrary.com/cdsr/doi/10.1002/14651858.CD003944.pub2/information 10.1002/14651858.CD003944.pub2 PMC6513376 30168578

[67] Barone P , Poewe W, Albrecht S, et al Pramipexole for the treatment of depressive symptoms in patients with Parkinson’s disease: A randomised, double-blind, placebo-controlled trial. Lancet Neurol. 2010;9(6):573–580. 20452823 10.1016/S1474-4422(10)70106-X

[68] Barone P , Scarzella L, Marconi R, et al Pramipexole versus sertraline in the treatment of depression in Parkinson’s disease: A national multicenter parallel-group randomized study. J Neurol. 2006;253(5):601–607. 16607468 10.1007/s00415-006-0067-5

[69] Joffe MM , Rosenbaum PR. Invited commentary: Propensity scores. Am J Epidemiol. 1999;150(4):327–333. 10453808 10.1093/oxfordjournals.aje.a010011

[70] Austin PC , Mamdani MM, Stukel TA, et al The use of the propensity score for estimating treatment effects: Administrative versus clinical data. Stat Med. 2005;24(10):1563–1578. 15706581 10.1002/sim.2053

[71] Schmidt AF , Groenwold RHH, van Delden JJM, et al Justification of exclusion criteria was underreported in a review of cardiovascular trials. J Clin Epidemiol. 2014;67(6):635–644. 24613498 10.1016/j.jclinepi.2013.12.005

[72] Black N . Why we need observational studies to evaluate the effectiveness of health care. BMJ. 1996;312(7040):1215–1218. 8634569 10.1136/bmj.312.7040.1215 PMC2350940

[73] Landwehrmeyer GB , Fitzer-Attas CJ, Giuliano JD, et al Data analytics from enroll-HD, a global clinical research platform for huntington’s disease. Mov Disord Clin Pract [date unknown];4(2):212–224. 10.1002/mdc3.12388 PMC6174428 30363395

[74] Sathe S , Ware J, Levey J, et al Enroll-HD: An integrated clinical research platform and worldwide observational study for huntington’s disease. Front Neurol. 2021;12:667420. 34484094 10.3389/fneur.2021.667420 PMC8416308

[75] Orth M , Handley OJ, Schwenke C, et al Observing huntington’s disease: The European huntington’s disease network’s REGISTRY. PLoS Curr. 2010;2:RRN1184. 10.1371/currents.RRN1184 PMC2947793 20890398

[76] Huntington Study Group . Unified huntington’s disease rating scale: Reliability and consistency. Mov Disord Off J Mov Disord Soc. 1996;11(2):136–142. 10.1002/mds.870110204 8684382

[77] Callaghan J , Stopford C, Arran N, et al Reliability and factor structure of the short problem behaviors assessment for huntington’s disease (PBA-s) in the TRACK-HD and REGISTRY studies. J Neuropsychiatry Clin Neurosci. 2015;27(1):59–64. 25716488 10.1176/appi.neuropsych.13070169

[78] Zigmond AS , Snaith RP. The hospital anxiety and depression scale. Acta Psychiatr Scand. 1983;67(6):361–370. 6880820 10.1111/j.1600-0447.1983.tb09716.x

[79] Thompson JC , Harris J, Sollom AC, et al Longitudinal evaluation of neuropsychiatric symptoms in huntington’s disease. J Neuropsychiatry Clin Neurosci. 2012;24(1):53–60. 22450614 10.1176/appi.neuropsych.11030057

[80] Hansotia P , Wall R, Berendes J. Sleep disturbances and severity of huntington’s disease. Neurology. 1985;35(11):1672. 2932657 10.1212/wnl.35.11.1672

[81] Herzog–Krzywoszanska R , Krzywoszanski L. Sleep disorders in huntington’s disease [internet]. Front Psychiatry. 2019;10. [cited 2020 Oct 20] Available from: https://www.ncbi.nlm.nih.gov/pmc/articles/PMC6474183/10.3389/fpsyt.2019.00221 PMC6474183 31031659

[82] Kalliolia E , Silajdžić E, Nambron R, et al Plasma melatonin is reduced in huntington’s disease. Mov Disord Off J Mov Disord Soc. 2014;29(12):1511–1515. 10.1002/mds.26003 25164424

[83] Bachoud-Lévi A-C , Ferreira J, Massart R, et al International guidelines for the treatment of huntington’s disease [internet]. Front Neurol. 2019;10. [cited 2019 Oct 22] Available from: https://www.frontiersin.org/articles/10.3389/fneur.2019.00710/full 10.3389/fneur.2019.00710 PMC6618900 31333565

[84] Anderson KE , van Duijn E, Craufurd D, et al Clinical management of neuropsychiatric symptoms of huntington disease: Expert-based consensus guidelines on agitation, anxiety, apathy, psychosis and sleep disorders. J Huntingt Dis [date unknown];7 (4):355–366. 10.3233/JHD-180293 PMC6294590 30040737

[85] Souza JD , Jones LA, Rickards H. Validation of self-report depression rating scales in huntington’s disease. Mov Disord [date unknown];25(1):91–96. 10.1002/mds.22837 19908314

[86] Ridgeway G , McCaffrey D, Morral A, et al twang: Toolkit for Weighting and Analysis of Nonequivalent Groups [Internet]. 2017.[cited 2019 Dec 23] Available from: https://CRAN.R-project.org/package=twang

[87] R Core Team. R: A Language and Environment for Statistical Computing [Internet]. Vienna, Austria: R Foundation for Statistical Computing; 2015.Available from: https://www.R-project.org/

[88] Schobel SA , Palermo G, Auinger P, et al Motor, cognitive, and functional declines contribute to a single progressive factor in early HD. Neurology. 2017;89(24):2495–2502. 29142089 10.1212/WNL.0000000000004743 PMC5729794

[89] McLauchlan D . Objective assessment of the neuropsychiatric symptoms in Huntington’s Disease [Internet]. 2018; [cited 2019 Jun 30] Available from: http://orca.cf.ac.uk/122117/

[90] Marin RS , Biedrzycki RC, Firinciogullari S. Reliability and validity of the apathy evaluation scale. Psychiatry Res. 1991;38(2):143–162. 1754629 10.1016/0165-1781(91)90040-v

[91] Carver CS , White TL. Behavioral inhibition, behavioral activation, and affective responses to impending reward and punishment: The BIS/BAS scales. J Pers Soc Psychol. 1994;67(2):319–333.

[92] Woody ML , Gibb BE. Integrating NIMH research domain criteria (RDoC) into depression research. Curr Opin Psychol. 2015;4:6–12. 25642446 10.1016/j.copsyc.2015.01.004 PMC4306327

[93] Phillips ML , Chase HW, Sheline YI, et al Identifying predictors, moderators, and mediators of antidepressant response in Major depressive disorder: Neuroimaging approaches. Am J Psychiatry. 2015;172(2):124–138. 25640931 10.1176/appi.ajp.2014.14010076 PMC4464814

[94] Cooper JA , Arulpragasam AR, Treadway MT. Anhedonia in depression: Biological mechanisms and computational models. Curr Opin Behav Sci. 2018;22:128–135. 29503842 10.1016/j.cobeha.2018.01.024 PMC5828520

[95] Cools R , Blackwell A, Clark L, et al Tryptophan depletion disrupts the motivational guidance of goal-directed behavior as a function of trait impulsivity. Neuropsychopharmacology. 2005;30(7):1362–1373. 15770237 10.1038/sj.npp.1300704

[96] Frank MJ , Seeberger LC, O’reilly RC. By carrot or by stick: Cognitive reinforcement learning in parkinsonism. Science. 2004;306(5703):1940–1943. 15528409 10.1126/science.1102941

[97] Pessiglione M , Vinckier F, Bouret S, et al Why not try harder? Computational approach to motivation deficits in neuro-psychiatric diseases. Brain. 2018;141(3):629–650. 29194534 10.1093/brain/awx278

[98] Mauras T , Masson M, Fossati P, Pessiglione M. Incentive sensitivity as a behavioral marker of clinical remission from Major depressive episode. J Clin Psychiatry. 2016;77(6):e697–703. 27337420 10.4088/JCP.15m09995

[99] Kr L DP , Jl L, Dm B. Depression risk predicts blunted neural responses to gains and enhanced responses to losses in healthy children [internet]. J Am Acad Child Adolesc Psychiatry. 2016;55(4). [cited 2020 Dec 2] Available from: https://pubmed.ncbi.nlm.nih.gov/27015724/10.1016/j.jaac.2016.01.007 PMC4808567 27015724

[100] Gotlib IH , Hamilton JP, Cooney RE, et al Neural processing of reward and loss in girls at risk for Major depression. Arch Gen Psychiatry. 2010;67(4):380. 20368513 10.1001/archgenpsychiatry.2010.13 PMC2852176

[101] Clark DA , Beck AT. Cognitive theory and therapy of anxiety and depression: Convergence with neurobiological findings. Trends Cogn Sci. 2010;14(9):418–424. 20655801 10.1016/j.tics.2010.06.007

[102] Ji JL , Holmes EA, MacLeod C, Murphy FC. Spontaneous cognition in dysphoria: Reduced positive bias in imagining the future [internet]. Psychol Res. 2019;83(4):817–831. 30097711 10.1007/s00426-018-1071-y PMC6529377

[103] MacLeod AK , Salaminiou E. Reduced positive future-thinking in depression: Cognitive and affective factors. Cogn Emot. 2001;15(1):99–107.

[104] MacLeod AK , Tata P, Tyrer P, et al Hopelessness and positive and negative future thinking in parasuicide. Br J Clin Psychol. 2005;44(4):495–504. 16368029 10.1348/014466505X35704

[105] Murphy FC , Michael A, Robbins TW, Sahakian BJ. Neuropsychological impairment in patients with major depressive disorder: The effects of feedback on task performance. Psychol Med. 2003;33(3):455–467. 12701666 10.1017/s0033291702007018

[106] von Gunten A , Herrmann FR, Elliott R, Duc R. Abnormal sensitivity to negative feedback in late-life depression. Psychiatry Clin Neurosci. 2011;65(4):333–340. 21569177 10.1111/j.1440-1819.2011.02215.x

[107] O’Rourke JJF , Adams WH, Duff K, et al Estimating premorbid functioning in huntington’s disease: The relationship between disease progression and the wide range achievement test Reading subtest. Arch Clin Neuropsychol Off J Natl Acad Neuropsychol. 2011;26(1):59–66. 10.1093/arclin/acq088 PMC3021970 21147861

[108] Crawford JR , Millar J, Milne AB. Estimating premorbid IQ from demographic variables: A comparison of a regression equation vs. Clinical judgement. Br J Clin Psychol. 2001;40(Pt 1):97–105. 11317952 10.1348/014466501163517

[109] Crawford JR , Parker DM, Besson JA. Estimation of premorbid intelligence in organic conditions. Br J Psychiatry J Ment Sci. 1988;153:178–181. 10.1192/bjp.153.2.178 2978378

[110] Leucht S , Samara M, Heres S, et al Dose equivalents for second-generation antipsychotics: The minimum effective dose method. Schizophr Bull. 2014;40(2):314–326. 24493852 10.1093/schbul/sbu001 PMC3932104

[111] Hayasaka Y , Purgato M, Magni LR, et al Dose equivalents of antidepressants: Evidence-based recommendations from randomized controlled trials. J Affect Disord. 2015;180:179–184. 25911132 10.1016/j.jad.2015.03.021

[112] Venables WN , Ripley BD. Modern applied statistics with S [internet]. 4th ed: Springer-Verlag; 2002. [cited 2018 Jun 6] Available from://www.springer.com/gb/book/9780387954578

[113] Bolker B, Team RDC. bbmle: Tools for General Maximum Likelihood Estimation [Internet]. 2017. Available from: https://CRAN.R-project.org/package=bbmle

[114] Magnusson A , Skaug H, Nielsen A, et al glmmTMB: Generalized Linear Mixed Models using Template Model Builder [Internet]. 2017. Available from: https://CRAN.R-project.org/package=glmmTMB

[115] Lo S , Andrews S. To transform or not to transform: Using generalized linear mixed models to analyse reaction time data [internet]. Front Psychol. 2015;6. [cited 2019 Feb 7] Available from: https://www.ncbi.nlm.nih.gov/pmc/articles/PMC4528092/10.3389/fpsyg.2015.01171 PMC4528092 26300841

[116] Woods DL , Wyma JM, Yund EW, et al Factors influencing the latency of simple reaction time [internet]. Front Hum Neurosci. 2015;9. [cited 2019 Feb 7] Available from: https://www.ncbi.nlm.nih.gov/pmc/articles/PMC4374455/10.3389/fnhum.2015.00131 PMC4374455 25859198

[117] Bolker BM , Brooks ME, Clark CJ, et al Generalized linear mixed models: A practical guide for ecology and evolution. Trends Ecol Evol. 2009;24(3):127–135. 19185386 10.1016/j.tree.2008.10.008

[118] Belujon P , Grace AA. Dopamine system dysregulation in Major depressive disorders. Int J Neuropsychopharmacol. 2017;20(12):1036–1046. 29106542 10.1093/ijnp/pyx056 PMC5716179

[119] Michely J , Viswanathan S, Hauser TU, et al The role of dopamine in dynamic effort-reward integration. Neuropsychopharmacology. 2020;45(9):1448–1453. 32268344 10.1038/s41386-020-0669-0 PMC7360543

[120] McGuigan S , Zhou S-H, Brosnan MB, et al Dopamine restores cognitive motivation in Parkinson’s disease. Brain. 2019;142(3):719–732. 30689734 10.1093/brain/awy341

[121] Boku S , Nakagawa S, Toda H, Hishimoto A. Neural basis of major depressive disorder: Beyond monoamine hypothesis. Psychiatry Clin Neurosci. 2018;72(1):3–12. 28926161 10.1111/pcn.12604

[122] Dean J , Keshavan M. The neurobiology of depression: An integrated view. Asian J. Psychiatry. 2017;27:101–111. 10.1016/j.ajp.2017.01.025 28558878

[123] Yoshida K , Higuchi H, Takahashi H, Shimizu T. Favorable effect of milnacipran on depression induced by interferon-alpha. J Neuropsychiatry Clin Neurosci. 2003;15(2):242–243. 12724470 10.1176/jnp.15.2.242

[124] Fornaro M , Rocchi G, Escelsior A, et al Might different cytokine trends in depressed patients receiving duloxetine indicate differential biological backgrounds. J Affect Disord. 2013;145(3):300–307. 22981313 10.1016/j.jad.2012.08.007

[125] Sarkar S , Schaefer M. Antidepressant pretreatment for the prevention of interferon alfa–associated depression: A systematic review and meta-analysis. Psychosomatics. 2014;55(3):221–234. 24012293 10.1016/j.psym.2013.06.015

[126] Ohgi Y , Futamura T, Kikuchi T, Hashimoto K. Effects of antidepressants on alternations in serum cytokines and depressive-like behavior in mice after lipopolysaccharide administration. Pharmacol Biochem Behav. 2013;103(4):853–859. 23262300 10.1016/j.pbb.2012.12.003

[127] Tynan RJ , Weidenhofer J, Hinwood M, et al A comparative examination of the anti-inflammatory effects of SSRI and SNRI antidepressants on LPS stimulated microglia. Brain Behav Immun. 2012;26(3):469–479. 22251606 10.1016/j.bbi.2011.12.011

[128] Nandam LS , Brazel M, Zhou M, Jhaveri DJ. Cortisol and Major depressive disorder—Translating findings from humans to animal models and back [internet]. Front Psychiatry. 2020;10. [cited 2020 Dec 4] Available from: https://www.frontiersin.org/articles/10.3389/fpsyt.2019.00974/full#supplementary-material 10.3389/fpsyt.2019.00974 PMC6987444 32038323

[129] Ransome MI , Renoir T, Hannan AJ. Hippocampal neurogenesis, cognitive deficits and affective disorder in huntington’s disease. Neural Plast. 2012;2012:e874387. 10.1155/2012/874387 PMC3394391 22830053

[130] Drew MR , Hen R. Adult hippocampal neurogenesis as target for the treatment of depression. CNS Neurol Disord Drug Targets. 2007;6(3):205–218. 17511617 10.2174/187152707780619353

[131] Zhou C , Zhong J, Zou B, et al Meta-analyses of comparative efficacy of antidepressant medications on peripheral BDNF concentration in patients with depression. PloS One. 2017;12(2):e0172270. 10.1371/journal.pone.0172270 PMC5328267 28241064

[132] Boldrini M , Hen R, Underwood MD, et al Hippocampal angiogenesis and progenitor cell proliferation are increased with antidepressant use in major depression. Biol Psychiatry. 2012;72(7):562–571. 22652019 10.1016/j.biopsych.2012.04.024 PMC3438317

[133] Boldrini M , Santiago AN, Hen R, et al Hippocampal granule neuron number and dentate gyrus volume in antidepressant-treated and untreated major depression. Neuropsychopharmacol Off Publ Am Coll Neuropsychopharmacol. 2013;38(6):1068–1077. 10.1038/npp.2013.5 PMC3629406 23303074

[134] Lino de Oliveira C , Bolzan JA, Surget A, Belzung C. Do antidepressants promote neurogenesis in adult hippocampus? A systematic review and meta-analysis on naive rodents. Pharmacol Ther. 2020;210:107515. 32109488 10.1016/j.pharmthera.2020.107515

[135] Peng Q , Masuda N, Jiang M, et al The antidepressant sertraline improves the phenotype, promotes neurogenesis and increases BDNF levels in the R6/2 huntington’s disease mouse model. Exp Neurol. 2008;210(1):154–163. 18096160 10.1016/j.expneurol.2007.10.015 PMC2278120

[136] Duan W , Peng Q, Masuda N, et al Sertraline slows disease progression and increases neurogenesis in N171-82Q mouse model of huntington’s disease. Neurobiol Dis. 2008;30(3):312–322. 18403212 10.1016/j.nbd.2008.01.015 PMC3683653

[137] Renoir T , Argyropoulos A, Hannan AJ. Antidepressant-Like effect of the norepinephrine-dopamine reuptake inhibitor bupropion in a mouse model of huntington’s disease with dopaminergic dysfunction. J Huntingt Dis. 2012;1(2):261–266. 10.3233/JHD-120039 25063334

[138] Renoir T , Pang TY, Zajac MS, et al Treatment of depressive-like behaviour in huntington’s disease mice by chronic sertraline and exercise. Br J Pharmacol. 2012;165(5):1375–1389. 21718306 10.1111/j.1476-5381.2011.01567.x PMC3372723

[139] Grote HE , Bull ND, Howard ML, et al Cognitive disorders and neurogenesis deficits in huntington’s disease mice are rescued by fluoxetine. Eur J Neurosci. 2005;22(8):2081–2088. 16262645 10.1111/j.1460-9568.2005.04365.x

[140] Duan W , Guo Z, Jiang H, et al Paroxetine retards disease onset and progression in huntingtin mutant mice. Ann Neurol. 2004;55(4):590–594. 15048901 10.1002/ana.20075

[141] Krogias C , Strassburger K, Eyding J, et al Depression in patients with huntington disease correlates with alterations of the brain stem raphe depicted by transcranial sonography. J Psychiatry Neurosci JPN. 2011;36(3):187–194. 21138658 10.1503/jpn.100067 PMC3080514

[142] van Wingen GA , Tendolkar I, Urner M, et al Short-term antidepressant administration reduces default mode and task-positive network connectivity in healthy individuals during rest. NeuroImage. 2014;88:47–53. 24269575 10.1016/j.neuroimage.2013.11.022

[143] Lisinski A , Hieronymus F, Näslund J, et al Item-based analysis of the effects of duloxetine in depression: A patient-level post hoc study. Neuropsychopharmacology. 2020;45(3):553–560. 31521062 10.1038/s41386-019-0523-4 PMC6969189

[144] Varazzani C , San-Galli A, Gilardeau S, Bouret S. Noradrenaline and dopamine neurons in the reward/effort trade-off: A direct electrophysiological comparison in behaving monkeys. J Neurosci. 2015;35(20):7866–7877. 25995472 10.1523/JNEUROSCI.0454-15.2015 PMC6795183

[145] Hosking JG , Floresco SB, Winstanley CA. Dopamine antagonism decreases willingness to expend physical, but not cognitive, effort: A comparison of two rodent cost/benefit decision-making tasks. Neuropsychopharmacol Off Publ Am Coll Neuropsychopharmacol. 2015;40(4):1005–1015. 10.1038/npp.2014.285 PMC4330516 25328051

[146] Jahn CI , Gilardeau S, Varazzani C, et al Dual contributions of noradrenaline to behavioural flexibility and motivation. Psychopharmacology (Berl). 2018;235(9):2687–2702. 29998349 10.1007/s00213-018-4963-z PMC6182595

[147] Borderies N , Bornert P, Gilardeau S, Bouret S. Pharmacological evidence for the implication of noradrenaline in effort. PLoS Biol. 2020;18(10):e3000793. 10.1371/journal.pbio.3000793 PMC7580990 33044952

[148] Subhan F , Deslandes PN, Pache DM, Sewell RD. Do antidepressants affect motivation in conditioned place preference? Eur J Pharmacol. 2000;408(3):257–263. 11090642 10.1016/s0014-2999(00)00771-8

[149] Martinotti G , Sepede G, Gambi F, et al Agomelatine versus venlafaxine XR in the treatment of anhedonia in major depressive disorder: A pilot study. J Clin Psychopharmacol. 2012;32(4):487–491. 22722509 10.1097/JCP.0b013e31825d6c25

[150] Stoy M , Schlagenhauf F, Sterzer P, et al Hyporeactivity of ventral striatum towards incentive stimuli in unmedicated depressed patients normalizes after treatment with escitalopram. J Psychopharmacol (Oxf). 2012;26(5):677–688. 10.1177/0269881111416686 21926423

[151] McCabe C , Mishor Z, Cowen PJ, Harmer CJ. Diminished neural processing of aversive and rewarding stimuli during selective serotonin reuptake inhibitor treatment. Biol Psychiatry. 2010;67(5):439–445. 20034615 10.1016/j.biopsych.2009.11.001 PMC2828549

[152] Padala PR , Padala KP, Monga V, et al Reversal of SSRI-associated apathy syndrome by discontinuation of therapy. Ann Pharmacother. 2012;46(3):e8. 22353235 10.1345/aph.1Q656

[153] Learned-Coughlin SM , Bergström M, Savitcheva I, et al In vivo activity of bupropion at the human dopamine transporter as measured by positron emission tomography. Biol Psychiatry. 2003;54(8):800–805. 14550679 10.1016/s0006-3223(02)01834-6

[154] Egerton A , Shotbolt JP, Stokes PRA, et al Acute effect of the anti-addiction drug bupropion on extracellular dopamine concentrations in the human striatum: An [11C]raclopride PET study. NeuroImage. 2010;50(1):260–266. 19969097 10.1016/j.neuroimage.2009.11.077 PMC4135078

[155] Yohn SE , Lopez-Cruz L, Hutson PH, et al Effects of lisdexamfetamine and s-citalopram, alone and in combination, on effort-related choice behavior in the rat. Psychopharmacology (Berl). 2016;233(6):949–960. 26694811 10.1007/s00213-015-4176-7

[156] Randall PA , Lee CA, Podurgiel SJ, et al Bupropion increases selection of high effort activity in rats tested on a progressive ratio/chow feeding choice procedure: Implications for treatment of effort-related motivational symptoms [internet]. Int J Neuropsychopharmacol. 2015;18(2). [cited 2019 Jun 18] Available from: https://academic.oup.com/ijnp/article/18/2/pyu017/690072. 10.1093/ijnp/pyu017 PMC4368885 25575584

[157] Randall PA , Lee CA, Nunes EJ, et al The VMAT-2 inhibitor tetrabenazine affects effort-related decision making in a progressive ratio/chow feeding choice task: Reversal with antidepressant drugs. PLoS One. 2014;9(6):e99320. 24937131 10.1371/journal.pone.0099320 PMC4061002

[158] Nunes EJ , Randall PA, Hart EE, et al Effort-Related motivational effects of the VMAT-2 inhibitor tetrabenazine: Implications for animal models of the motivational symptoms of depression. J Neurosci. 2013;33(49):19120–19130. 24305809 10.1523/JNEUROSCI.2730-13.2013 PMC3850037

[159] Fischer AG , Ullsperger M. An update on the role of serotonin and its interplay with dopamine for reward [internet]. Front Hum Neurosci. 2017;11. [cited 2019 Oct 7] Available from: https://www.ncbi.nlm.nih.gov/pmc/articles/PMC5641585/10.3389/fnhum.2017.00484 PMC5641585 29075184

[160] Correia PA , Lottem E, Banerjee D, et al Transient inhibition and long-term facilitation of locomotion by phasic optogenetic activation of serotonin neurons. eLife. 2017;6:e20975. 28193320 10.7554/eLife.20975 PMC5308893

[161] Bailey MR , Goldman O, Bello EP, et al An interaction between serotonin receptor signaling and dopamine enhances goal-directed vigor and persistence in mice. J Neurosci. 2018;38(9):2149–2162. 29367407 10.1523/JNEUROSCI.2088-17.2018 PMC5830508

[162] Meyniel F , Goodwin GM, Deakin JW, et al A specific role for serotonin in overcoming effort cost. eLife. 2016;5:e17282. 27824554 10.7554/eLife.17282 PMC5100997

[163] Scholl J , Kolling N, Nelissen N, et al Beyond negative valence: 2-week administration of a serotonergic antidepressant enhances both reward and effort learning signals. PLoS Biol. 2017;15(2):e2000756. 10.1371/journal.pbio.2000756 PMC5331946 28207733

[164] Guitart-Masip M , Economides M, Huys QJM, et al Differential, but not opponent, effects of L -DOPA and citalopram on action learning with reward and punishment. Psychopharmacology (Berl). 2014;231(5):955–966. 24232442 10.1007/s00213-013-3313-4 PMC3923110

[165] Walsh AEL , Huneke NTM, Brown R, et al A dissociation of the acute effects of bupropion on positive emotional processing and reward processing in healthy volunteers. Front Psychiatry. 2018;9:482. 30386259 10.3389/fpsyt.2018.00482 PMC6198095

[166] Walsh A , Browning M, Drevets W C, et al Dissociable temporal effects of bupropion on behavioural measures of emotional and reward processing in depression. Philos Trans R Soc Lond B Biol Sci. 2018:373. 10.1098/rstb.2017.0030 PMC5790828 29352029

[167] Dean Z , Horndasch S, Giannopoulos P, McCabe C. Enhanced neural response to anticipation, effort and consummation of reward and aversion during bupropion treatment. Psychol Med. 2016;46(11):2263–2274. 27188979 10.1017/S003329171600088X

[168] Tomarken AJ , Dichter GS, Freid C, et al Assessing the effects of bupropion SR on mood dimensions of depression. J Affect Disord. 2004;78(3):235–241. 15013248 10.1016/S0165-0327(02)00306-3

[169] Gaynes BN , Farley JF, Dusetzina SB, et al Does the presence of accompanying symptom clusters differentiate the comparative effectiveness of second-line medication strategies for treating depression? Depress. Anxiety. 2011;28(11):989–998. 21898717 10.1002/da.20898 PMC3215789

[170] Jamerson BD , Krishnan KRR, Roberts J, et al Effect of bupropion SR on specific symptom clusters of depression: Analysis of the 31-item Hamilton rating scale for depression. Psychopharmacol Bull. 2003;37(2):67–78. 14566216

[171] Cooper JA , Tucker VL, Papakostas GI. Resolution of sleepiness and fatigue: A comparison of bupropion and selective serotonin reuptake inhibitors in subjects with major depressive disorder achieving remission at doses approved in the European union. J Psychopharmacol Oxf Engl. 2014;28(2):118–124. 10.1177/0269881113514878 24352716

[172] Chong TT-J , Apps M, Giehl K, et al Neurocomputational mechanisms underlying subjective valuation of effort costs. PLoS Biol. 2017;15(2):e1002598. 10.1371/journal.pbio.1002598 PMC5325181 28234892

[173] Hauser TU , Eldar E, Dolan RJ. Separate mesocortical and mesolimbic pathways encode effort and reward learning signals. Proc Natl Acad Sci. 2017;114(35):E7395–E7404. 28808037 10.1073/pnas.1705643114 PMC5584432

[174] Klein-Flügge MC , Kennerley SW, Saraiva AC, et al Behavioral modeling of human choices reveals dissociable effects of physical effort and temporal delay on reward devaluation. PLoS Comput Biol. 2015;11(3):e1004116. 10.1371/journal.pcbi.1004116 PMC4376637 25816114

[175] Vonsattel JPG , Difiglia M. Huntington disease. J Neuropathol Exp Neurol. 1998;57(5):369–384. 9596408 10.1097/00005072-199805000-00001

[176] Vonsattel JP , Myers RH, Stevens TJ, et al Neuropathological classification of huntington’s disease. J Neuropathol Exp Neurol. 1985;44(6):559–577. 2932539 10.1097/00005072-198511000-00003

[177] Rüb U , Seidel K, Heinsen H, et al Huntington’s disease (HD): The neuropathology of a multisystem neurodegenerative disorder of the human brain. Brain Pathol Zurich Switz. 2016;26(6):726–740. 10.1111/bpa.12426 PMC8029421 27529157

[178] Thu DCV , Oorschot DE, Tippett LJ, et al Cell loss in the motor and cingulate cortex correlates with symptomatology in huntington’s disease. Brain J Neurol. 2010;133(Pt 4):1094–1110. 10.1093/brain/awq047 20375136

[179] Nana AL , Kim EH, Thu DCV, et al Widespread heterogeneous neuronal loss across the cerebral cortex in huntington’s disease. J Huntingt Dis. 2014;3(1):45–64. 10.3233/JHD-140092 25062764

[180] Holroyd CB , Yeung N. Motivation of extended behaviors by anterior cingulate cortex. Trends Cogn Sci. 2012;16(2):122–128. 22226543 10.1016/j.tics.2011.12.008

[181] Botvinick MM , Huffstetler S, McGuire JT. Effort discounting in human nucleus accumbens. Cogn Affect Behav Neurosci. 2009;9(1):16–27. 19246324 10.3758/CABN.9.1.16 PMC2744387

[182] Massar SAA , Libedinsky C, Weiyan C, et al Separate and overlapping brain areas encode subjective value during delay and effort discounting. NeuroImage. 2015;120:104–113. 26163803 10.1016/j.neuroimage.2015.06.080

[183] Floresco SB , Tse MTL, Ghods-Sharifi S. Dopaminergic and glutamatergic regulation of effort- and delay-based decision making. Neuropsychopharmacology. 2008;33(8):1966–1979. 17805307 10.1038/sj.npp.1301565

[184] Treadway MT , Buckholtz JW, Cowan RL, et al Dopaminergic mechanisms of individual differences in human effort-based decision-making. J Neurosci Off J Soc Neurosci. 2012;32(18):6170–6176. 10.1523/JNEUROSCI.6459-11.2012 PMC3391699 22553023

[185] Salamone JD , Correa M, Farrar A, Mingote SM. Effort-related functions of nucleus accumbens dopamine and associated forebrain circuits. Psychopharmacology (Berl). 2007;191(3):461–482. 17225164 10.1007/s00213-006-0668-9

[186] Salamone JD , Yohn SE, López-Cruz L, et al Activational and effort-related aspects of motivation: Neural mechanisms and implications for psychopathology. Brain. 2016;139(5):1325–1347. 27189581 10.1093/brain/aww050 PMC5839596

[187] Sprengelmeyer R , Müller H-P, Süssmuth SD, et al K02 the neuroanatomy of depression: Evidence from huntington’s disease. J Neurol Neurosurg Psychiatry. 2012;83(Suppl 1):A41–A42.

[188] Sprengelmeyer R , Orth M, Müller H-P, et al The neuroanatomy of subthreshold depressive symptoms in huntington’s disease: A combined diffusion tensor imaging (DTI) and voxel-based morphometry (VBM) study. Psychol Med. 2014;44(9):1867–1878. 24093462 10.1017/S003329171300247X

[189] Chang EP , Ecker UKH, Page AC. Impaired memory updating associated with impaired recall of negative words in dysphoric rumination—Evidence for a removal deficit. Behav Res Ther. 2017;93:22–28. 28347841 10.1016/j.brat.2017.03.008

[190] Enzi B , Edel M-A, Lissek S, et al Altered ventral striatal activation during reward and punishment processing in premanifest huntington’s disease: A functional magnetic resonance study. Exp Neurol. 2012;235(1):256–264. 22366326 10.1016/j.expneurol.2012.02.003

[191] Henley SMD , Novak MJU, Frost C, et al Emotion recognition in huntington’s disease: A systematic review. Neurosci Biobehav Rev. 2012;36(1):237–253. 21699916 10.1016/j.neubiorev.2011.06.002

[192] Ille R , Schäfer A, Scharmüller W, et al Emotion recognition and experience in huntington disease: A voxel-based morphometry study. J Psychiatry Neurosci JPN. 2011;36(6):383–390. 21406159 10.1503/jpn.100143 PMC3201992

[193] Heim B , Peball M, Saft C, et al Tit for tat: Costly punishment in manifest huntington’s disease [internet]. Neurodegener Dis. 2021. [cited 2022 Feb 15] Available from: https://www.karger.com/Article/FullText/520303 10.1159/000520303 34706364

[194] Heim B , Peball M, Saft C, et al Time will tell: Decision making in premanifest and manifest huntington’s disease. Brain Behav. 2020;10(11):e01843. 32978893 10.1002/brb3.1843 PMC7667290

[195] Brüne M , von Hein SM, Claassen C, et al Altered third-party punishment in huntington’s disease: A study using neuroeconomic games. Brain Behav. 2021;11(1):e01908. 33070471 10.1002/brb3.1908 PMC7821630

[196] Le Heron C , Plant O, Manohar S, et al Distinct effects of apathy and dopamine on effort-based decision-making in Parkinson’s disease. Brain J Neurol. 2018;141(5):1455–1469. 10.1093/brain/awy110 PMC5917786 29672668

[197] Le Heron C , Manohar S, Plant O, et al Dysfunctional effort-based decision-making underlies apathy in genetic cerebral small vessel disease. Brain. 2018;141(11):3193–3210. 30346491 10.1093/brain/awy257 PMC6202575

[198] Fervaha G , Graff-Guerrero A, Zakzanis KK, et al Incentive motivation deficits in schizophrenia reflect effort computation impairments during cost-benefit decision-making. J Psychiatr Res. 2013;47(11):1590–1596. 23992770 10.1016/j.jpsychires.2013.08.003

[199] Gold JM , Strauss GP, Waltz JA, et al Negative symptoms of schizophrenia are associated with abnormal effort-cost computations. Biol Psychiatry. 2013;74(2):130–136. 23394903 10.1016/j.biopsych.2012.12.022 PMC3703817

[200] Heath CJ , O’Callaghan C, Mason SL, et al A touchscreen motivation assessment evaluated in huntington’s disease patients and R6/1 model mice [internet]. Front Neurol. 2019;10. [cited 2019 Oct 25] Available from: https://www.frontiersin.org/articles/10.3389/fneur.2019.00858/full. 10.3389/fneur.2019.00858 PMC6696591 31447770

[201] Sharma A , Wolf DH, Ciric R, et al Common dimensional reward deficits across mood and psychotic disorders: A connectome-wide association study. Am J Psychiatry. 2017;174(7):657–666. 28135847 10.1176/appi.ajp.2016.16070774 PMC5495611

