# Correction to: Different depression: motivational anhedonia governs antidepressant efficacy in Huntington's disease

**DOI:** 10.1093/braincomms/fcad188

**Published:** 2023-06-22

This is a correction to: Duncan James McLauchlan and others, Different depression: motivational anhedonia governs antidepressant efficacy in Huntington's disease, *Brain Communications*, Volume 4, Issue 6, 2022, fcac278, https://doi.org/10.1093/braincomms/fcac278

In the originally published version of this manuscript, an erroneous supplementary file was published. The correct supplementary file has now been supplied to the article.

