# Supplementary material for: Different depression: motivational anhedonia governs antidepressant efficacy in Huntington’s disease
Source: Brain Commun. 2022 Nov 9;4(6):fcac278. doi: 10.1093/braincomms/fcac278 (PMC9683390; doi:10.1093/braincomms/fcac278)
Supplement: fcac278_Supplementary_Data [file fcac278_supplementary_data.pdf]

Table 1: Low Mood Indications for Antidepressant Treatment

”Adjustment disorder with depressed mood”
”Adjustment disorder with mixed anxiety and depressed mood”
”Affective disorder”
”Agitated depression”
”Depressed mood”
”Depression”
”Depressive symptom”
”Dysthymic disorder”
”Grief reaction”
”Major depression”
”Mood altered”
”Mood disorder due to a general medical condition”
”Negative thoughts”
”Postpartum depression”
”Suicidal ideation”
”Tearfulness”

Table 2: Drug Class and Frequency of Antidepressant Use in Huntington's Disease

Medication	Frequency	Percentage	Drug Class
Citalopram	1997.00	20.03	SSRI
Sertraline	1714.00	17.20	SSRI
Venlafaxine	1103.00	11.07	SNRI
Escitalopram	1071.00	10.74	SSRI
Mirtazapine	1033.00	10.36	TeCA
Paroxetine	672.00	6.74	SSRI
Fluoxetine	657.00	6.59	SSRI
Bupropion	441.00	4.42	NDRI
Duloxetine	317.00	3.18	SNRI
Trazodone	174.00	1.75	Phenylpiperazine
Amitriptyline	155.00	1.55	TCA
Mianserin	100.00	1.00	TeCA
Clomipramine	79.00	0.79	TCA
Desvenlafaxine	75.00	0.75	SNRI
Fluvoxamine	56.00	0.56	SSRI
Doxepin	51.00	0.51	TCA
Nortriptyline	45.00	0.45	TCA
Opipramol	45.00	0.45	TCA
Agomelatine	34.00	0.34	Unique
Tianeptine	21.00	0.21	Unique
Moclobemide	20.00	0.20	MAOI
Trimipramine	20.00	0.20	TCA
Dosulepin	15.00	0.15	TCA
Reboxetine	13.00	0.13	SNRI
Lofepramine	12.00	0.12	TCA
Vilazodone	12.00	0.12	SSRI
Imipramine	10.00	0.10	TCA
Milnacipran	10.00	0.10	SNRI
Levomilnacipran	6.00	0.06	SNRI
Oxitriptan	3.00	0.03	Unique
Ademetionine	2.00	0.02	Unique
Desipramine	2.00	0.02	TCA
Amitriptylinoxide	1.00	0.01	TCA
Maprotiline	1.00	0.01	TeCA
Nefazodone	1.00	0.01	Phenylpiperazine

Drug name missing in <0.05% of records
Frequency includes total number of prescriptions - including dose escalations
Abbreviations: SSRI - Selective Serotonin Reuptake Inhibitor,SNRI - Serotonin Noradrenaline Reuptake Inhibitor, TeCA - Tetracyclic Antidepressant, TCA - Tricyclic Antidepressant, MAOI - Monoamine Oxidase Inhibitor, NDRI - Noradrenaline Dopamine Reuptake inhibitor
MAOI* - For the purposes of the analysis, Moclobemide was included in the Unique class

Table 3
ATE Analysis of Drug Class, SSRI As Reference Treatment, Apathy Control Model

Outcome: Depression at First Follow-Up

	Estimate	P Value
(Intercept)	0.72	$< 2\text{x}10^{-16}$
NDRI	-0.080	0.22
Phenylpiperazine	0.14	0.0034
SNRI	0.051	0.054
TCA	0.081	0.39
TeCA	0.032	0.37
Unique	0.22	$1.64\text{x}10^{-6}$

ATE Analysis of Drug Class with Doubly Robust Estimation, SSRI as Reference Treatment, Apathy Control Model

Outcome: Depression at First Follow-Up

	Estimate	P Value
(Intercept)	0.6068	$9.84\text{x}10^{-5}$
NDRI	0.13	0.11
Phenylpiperazine	0.063	0.34
SNRI	0.093	0.0052
TCA	0.14	0.12
TeCA	0.072	0.063
Unique	0.16	0.00059
Age	0.0002	0.93
Sex(Male)	-0.030	0.54
Disease Progression Score	0.0025	0.72
PBAs Apathy Score	0.020	$5.10\text{x}10^{-6}$
SSRI Risk Factors	-0.022	0.73
Comorbidities	-0.026	0.59
Number of Antidepressants	0.0061	0.19
Sedative	0.033	0.65
Fluoxetine Equivalent Dose (mg)	$1.022\text{x}10^{-6}$	0.86

Abbreviations: ATE - Actual Treatment Effect,
SSRI - Selective Serotonin Reuptake Inhibitor,
NDRI - Norepinephrine–Dopamine Reuptake Inhibitor,
SNRI - Serotonin-Norepinephrine Reuptake Inhibitor,
TCA - Tricyclic Antidepressant, TeCA - Tetracyclic Antidepressants,
PBAs - Problem Behaviours Assessment (short form)

Table 4:
ATE Analysis of Drug Class, SSRI As Reference Treatment,
Apathy Control Model

Outcome: Depression at All Follow-Ups

	Estimate	P Value
(Intercept)	0.6835	$<2x10^{-16}$
NDRI	-0.080	0.011
Phenylpiperazine	0.063	0.022
SNRI	0.031	0.013
TCA	0.094	0.024
TeCA	0.077	$7.32x10^{-7}$
Unique	0.20	$1.66x10^{-5}$

ATE Analysis of Drug Class with Doubly Robust Estimation,
SSRI As Reference Treatment, Apathy Control Model

Outcome: Depression at All Follow-Ups

	Estimate	P Value
(Intercept)	0.57	$2.6x10^{-12}$
NDRI	0.029	0.50
Phenylpiperazine	0.014	0.70
SNRI	0.052	0.00098
TCA	0.071	0.19
TeCA	0.037	0.057
Unique	0.15	0.0052
Age	$1.078x10^{-4}$	0.92
Sex (Male)	-0.0047	0.84
Disease Progression Score	-0.0030	0.33
PBAs Apathy Score	0.026	$<2x10^{-16}$
SSRI Risk Factors	-0.021	0.44
Comorbidities	0.043	0.074
Number of Antidepressants	0.0064	0.0040
Sedative	0.046	0.20
Fluoxetine Equivalent Dose(mg)	0.0000	0.53

Abbreviations: ATE - Actual Treatment Effect,
SSRI - Selective Serotonin Reuptake Inhibitor,
NDRI - Norepinephrine–Dopamine Reuptake Inhibitor,
SNRI - Serotonin-Norepinephrine Reuptake Inhibitor,
TCA - Tricyclic Antidepressant, TeCA - Tetracyclic Antidepressants,
PBAs - Problem Behaviours Assessment (short form)

Table 5
ATE Analysis of Drug Class, SSRI as Reference Treatment,
Depression Control Model

Outcome: Apathy at First Follow-Up

	Estimate	P Value
(Intercept)	4.19	$< 2x10^{-16}$
NDRI	-0.061	0.92
Phenylpiperazine	-0.33	0.57
SNRI	0.79	0.0022
TCA	-0.25	0.81
TeCA	1.049	0.0038
Unique	1.34	0.36

ATE Analysis of Drug Class with Doubly Robust Estimation,
SSRI as Reference Treatment, Depression Control Model

Outcome: Apathy at First Follow-Up

	Estimate	P Value
(Intercept)	1.51	0.22
NDRI	0.23	0.81
Phenylpiperazine	-0.69	0.31
SNRI	0.39	0.23
TCA	-0.94	0.24
TeCA	0.72	0.15
Unique	2.96	$4.87x10^{-5}$
Age	0.0057	0.77
Sex(Male)	1.48	0.0024
Disease Progression Score	-0.21	$3.85x10^{-6}$
PBAs Depressed Mood Score	0.47	$< 2x10^{-16}$
SSRI Risk Factors	0.024	0.96
Comorbidities	-0.24	0.62
Number of Antidepressants	0.33	$2.96x10^{-9}$
Sedative	1.46	0.0077
Fluoxetine Equivalent Dose(mg)	0.00025	0.0035

Abbreviations: ATE - Actual Treatment Effect,
SSRI - Selective Serotonin Reuptake Inhibitor,
NDRI - Norepinephrine–Dopamine Reuptake Inhibitor,
SNRI - Serotonin-Norepinephrine Reuptake Inhibitor,
TCA - Tricyclic Antidepressant, TeCA - Tetracyclic Antidepressants,
PBAs - Problem Behaviours Assessment (short form)

Table 6
ATE Analysis of Drug Class, SSRI as Reference Treatment,
Depression Control Model

Outcome: Apathy at All Follow-Ups

	Estimate	P Value
(Intercept)	4.31	$< 2x10^{-16}$
NDRI	-1.19	$1.18x10^{-6}$
Phenylpiperazine	0.34	0.27
SNRI	0.092	0.41
TCA	0.44	0.32
TeCA	0.59	0.00021
Unique	-0.51	0.57

ATE Analysis of Drug Class with Doubly Robust Estimation, SSRI as Reference Treatment,
Depression Control Model

Outcome: Apathy at All Follow-Ups

	Estimate	P Value
(Intercept)	2.87	$7.62x10^{-7}$
NDRI	-0.35	0.37
Phenylpiperazine	-0.33	0.31
SNRI	0.056	0.71
TCA	0.072	0.89
TeCA	-0.16	0.44
Unique	2.18	0.16
Age	0.0020	0.82
Sex(Male)	0.63	0.0034
Disease Progression Score	-0.23	$< 2x10^{-16}$
PBAs Depressed Mood Score	0.48	$< 2x10^{-16}$
SSRI Risk Factors	-0.046	0.86
Comorbidities	-0.031	0.90
Number of Antidepressants	0.17	$2.53x10^{-12}$
Sedative	1.26	$3.42x10^{-7}$
Fluoxetine Equivalent Dose(mg)	0.0001	0.11

Abbreviations: ATE - Actual Treatment Effect,
SSRI - Selective Serotonin Reuptake Inhibitor,
NDRI - Norepinephrine–Dopamine Reuptake Inhibitor,
SNRI - Serotonin-Norepinephrine Reuptake Inhibitor,
TCA - Tricyclic Antidepressant, TeCA - Tetracyclic Antidepressants,
PBAs - Problem Behaviours Assessment (short form)

Table 7: Effect of Reward Value and Case Status on Reaction Time in Whole Group

	Dependent Variable: Reaction Time	
	Estimate	P Value
(Intercept)	5.69	$< 2x10^{-16}$
Block Order	0.086	0.0017
Maximum Reward	0.0080	0.00013
Case HD	0.22	0.032
TMS	0.0097	$4.98x10^{-5}$
Block Order * Maximum Reward	−0.0027	$3.63x10^{-5}$
Observations	6,884	
Log Likelihood	−46,392	
Akaike Inf. Crit.	92,799	
Bayesian Inf. Crit.	92,854	

* denotes interaction
HD - Huntington's disease, TMS - total motor score

Table 8: Effect of Reward Value and Depression on Reaction Time in Cases

	Dependent Variable: Reaction Time	
	Estimate	P Value
(Intercept)	5.61	$< 2x10^{-16}$
Maximum Reward	0.01	0.013
Block Order	0.29	$2.25x10^{-8}$
HADS Depression Score	0.045	0.014
TMS	0.0099	0.00015
Maximum Reward * HADS Depression Score * Block Order	0.00059	$1.38x10^{-8}$
Observations	3,873	
Log Likelihood	−27,197	
Akaike Inf. Crit.	54,417	
Bayesian Inf. Crit.	54,486	

* denotes interaction
HD - Huntington's disease, TMS - total motor score,
HADS - Hospital Anxiety & Depression Scale

Table 9: Effect of Reward Value and Apathy on Reaction Time in Cases

	Dependent Variable: Reaction Time	
	Estimate	P Value
(Intercept)	5.59	$< 2x10^{-16}$
Maximum Reward	0.026	0.059
Block Order	0.076	0.67
AES Score	0.24	0.42
TMS	0.0079	0.00043
Maximum Reward * AES Score * Block Order	0.0014	0.66
Observations	3,873	
Log Likelihood	−27,208	
Akaike Inf. Crit.	54,438	
Bayesian Inf. Crit.	54,507	

* denotes interaction

HD - Huntington's disease, TMS - total motor score, AES - Apathy Evaluation Scale

Table 10: Effect of Reward Value on Reaction Time in Whole Group

	Dependent variable: Reaction Time	
	Estimate	P Value
(Intercept)	5.76	$<2x10^{-16}$
Block Order	0.086	0.0016
Maximum Reward	0.0080	0.00013
TMS	0.013	$1.63x10^{-11}$
Block Order * Maximum Reward	−0.0027	$3.51x10^{-5}$
Observations	6,884	
Log Likelihood	−46,394	
Akaike Inf. Crit.	92,801	
Bayesian Inf. Crit.	92,849	

Note: *p<0.1; **p<0.05; ***p<0.01

Table 11: Group Effect- BISBAS Reward Score

	Dependent variable: BISBAS Reward Score	
	Estimate	P Value
(Intercept)	2.86	0.0000000
Case HD	−0.064	0.28
Observations	71	
Log Likelihood	−186	
Akaike Inf. Crit.	375	

HD - Huntington's disease, BISBAS - Behavioural Inhibition Scale & Behavioural Activation Scale

Table 12: BISBAS Reward Score and HADS Depression Score

	*Dependent variable:*HADS Depression Score	
	Estimate	P Value
(Intercept)	3.94	0.25
BISBAS Reward Score	0.11	0.57
Observations	45	
R^2	0.0074	
Adjusted R^2	−0.016	
F Statistic	0.32 (df = 1; 43)	
(Intercept)	0.55	0.82
BISBAS Reward Score	0.087	0.54
PBA Disorientation	0.57	0.0032
PBA Irritability	0.48	0.00030
PBA Anxiety	0.46	0.00066
Observations	45	
R^2	0.55	
Adjusted R^2	0.50	
F Statistic	12.034*** (df = 4; 40)	

PBA - Problem Behaviours Assessment, HADS - Hospital Anxiety & Depression Scale
BISBAS - Behavioural Inhibition Scale & Behavioural Activation Scale

Table 13: Group Effect- PSLT Learning

	Dependent variable: PSLT Total Learning Score	
	Estimate	P Value
(Intercept)	67.42	<2x10^{-16}
Case HD	-12.54	0.0092
Observations	55	
Log Likelihood	−234.36	
Akaike Inf. Crit.	472.71	

PSLT - Probabilistic Selection Learning Task, HD - Huntington's disease

Table 14: Group Effect- PSLT Criterion

	Dependent variable: PSLT Trials to Criterion	
(Intercept)	5.31	<2x10^{-16}
Case HD	0.45	0.00079
Observations	55	
Log Likelihood	−341.55	
Akaike Inf. Crit.	687.11	

PSLT - Probabilistic Selection Learning Task, HD - Huntington's disease

Table 15: Group Effect - PSLT Reward Learning

	Dependent variable: PSLT Reward Learning Score	
	Estimate	P Value
(Intercept)	4.12	<2x10^{-16}
Case HD	−0.090	0.56
Observations	55	
Log Likelihood	−267	
Akaike Inf. Crit.	539	

PSLT - Probabilistic Selection Learning Task, HD - Huntington's disease
NB: No variables improved the model

Table 16: Group Effect- PSLT Punishment Learning

	Dependent variable: PSLT Punishment Learning Score	
(Intercept)	4.29	<2x10^{-16}
Case HD	−0.29	0.00072
Observations	55	
Log Likelihood	−242	
Akaike Inf. Crit.	489	
(Intercept)	4.34	<2x10^{-16}
Case HD	−0.17	0.13
Sex (Male)	−0.16	0.061
TMS	−0.0057	0.018
Fluoxetine Equivalent Dose (mg)	0.0038	0.060
Observations	54	
Log Likelihood	−231	
Akaike Inf. Crit.	475	

PSLT - Probabilistic Selection Learning Task, HD - Huntington's disease
TMS - Total Motor Score

Table 17: PSLT Reward Learning and HADS Depression Score

	Dependent variable: HADS Depression Score	
	Estimate	P Value
(Intercept)	1.93	2.83x10^{-6}
PSLT Reward Learning Score	−0.0047	0.49
Observations	29	
Log Likelihood	−79	
Akaike Inf. Crit.	161	

NB: No variables improved the model
HADS - Hospital Anxiety & Depression Scale, PSLT - probabilistic selection learning task

Table 18: PSLT Learning, Interaction Model and HADS Depression Score

	Dependent variable: HADS Depression Score	
	Estimate	P Value
(Intercept)	3.57	0.00023
Reward Learning Score	−0.033	0.055
Punishment Learning Score	−0.035	0.070
Reward Learning Score * Punishment Learning Score	0.00058	0.083
Observations	29	
Log Likelihood	−77	
Akaike Inf. Crit.	162	
(Intercept)	2.86	0.015
Reward Learning Score	−0.025	0.16
Punishment Learning Score	−0.025	0.23
TMS	0.0060	0.36
Reward Learning Score * Punishment Learning Score	0.00044	0.21
Observations	29	
Log Likelihood	−77	
Akaike Inf. Crit.	163	

HADS - Hospital Anxiety & Depression Scale, PSLT - probabilistic selection learning task

Table 19: PSLT Punishment Learning and AES Score

	Dependent variable: AES Score	
	Estimate	P Value
(Intercept)	4.0048	$<2x10^{-16}$
Punishment Learning Score	−0.0079	0.036
Observations	29	
Log Likelihood	−118	
Akaike Inf. Crit.	240	
(Intercept)	3.17	$<2x10^{-16}$
Punishment Learning Score	−0.0011	0.70
Olanzapine Equivalent Dose (mg)	0.079	0.00013
TMS	0.011	0.000013
Observations	29	
Log Likelihood	−107	
Akaike Inf. Crit.	222	

AES - Apathy Evaluation Scale, PSLT - probabilistic selection learning task
TMS - Total Motor Score

Table 20: PSLT Learning, Interaction Model and AES Score

	Dependent variable: AES Score	
	Estimate	P Value
(Intercept)	5.16	p = 0.0000000
Reward Learning Score	−0.022	0.0080
Punishment Learning Score	−0.027	0.0052
Reward Learning Score * Punishment Learning Score	0.00036	0.030
Observations	29	
Log Likelihood	−115	
Akaike Inf. Crit.	239	
(Intercept)	3.83	
Reward Learning Score	−0.011	0.10
Punishment Learning Score	−0.012	0.13
Olanzapine Equivalent Dose (mg)	0.076	0.00015
TMS	0.0090	0.00030
Reward Learning Score * Punishment Learning Score	0.00020	0.14
Observations	29	
Log Likelihood	−106	
Akaike Inf. Crit.	223	

AES - Apathy Evaluation Scale, PSLT - probabilistic selection learning task
TMS - Total Motor Score

Table 21: Group Effect- Post Task Estimate

	Dependent variable: Post-Task Estimate	
	Estimate	P Value
(Intercept)	3.76	$<2x10^{-16}$
Case HD	-0.27	$5.13x10^{-11}$
Observations	64	
Log Likelihood	$-1{,}256$	
Akaike Inf. Crit.	2,516	
(Intercept)	5.34	$<2x10^{-16}$
Case HD	-0.58	$<2x10^{-16}$
Age	0.011	$1.61x10^{-13}$
Gender (Male)	0.30	$1.33x10^{-10}$
IQ	-0.022	$<2x10^{-16}$
Olanzapine Equivalent Dose	-0.062	$5.15x10^{-7}$
PBA Apathy	0.024	0.0017
PBA Anxiety	0.041	$1.79x10^{-6}$
PBA Irritability	-0.028	0.00059
PBA Disorientation	0.11	$2.91x10^{-10}$
Observations	61	
Log Likelihood	$-1{,}040$	
Akaike Inf. Crit.	2,100	

HD - Huntington's disease, PBA - Problem Behaviours Assessment

Table 22: Post-Task Estimate and HADS Depression Score

	*Dependent variable:*HADS Depression Score	
	Estimate	P Value
(Intercept)	1.65	$<2x10^{-16}$
Post-Task Estimate	0.00055	0.89
Observations	38	
Log Likelihood	-103	
Akaike Inf. Crit.	209	
(Intercept)	1.08	$7.27x10^{-12}$
Post-Task Estimate	-0.00075	0.77
PBA Irritability	0.070	0.0017
PBA Anxiety	0.12	$7.98x10^{-5}$
Observations	38	
Log Likelihood	-91	
Akaike Inf. Crit.	190	

PBA - Problem Behaviours Assessment, HADS - Hospital Anxiety & Depression Scale

Table 23: Group Effect - Estimate Change from Baseline

	Dependent variable: Estimate Change from Baseline	
	Estimate	P Value
(Intercept)	24.27	0.00022
Case HD	−9.64	0.22
Observations	69	
R^2	0.022	
Adjusted R^2	0.0073	
Residual Std. Error	31.68(df = 67)	
F Statistic	1.50 (df = 1; 67)	
(Intercept)	−31.71	0.42
Case HD	8.28	0.40
Age	−0.57	0.032
IQ	0.77	0.024
TMS	−0.14	0.56
Olanzapine Equivalent Dose	−1.46	0.40
PBA Disorientation	−3.29	0.20
Observations	65	
R^2	0.27	
Adjusted R^2	0.20	
F Statistic	3.66^{***} (df = 6; 58)	

Table 24: Estimate Change from Baseline and HADS Depression Score

	Dependent variable: HADS Depression Score	
	Estimate	P Value
(Intercept)	5.98	5.4×10^{-12}
Estimate - Change from Baseline	−0.024	0.16
Observations	43	
R^2	0.048	
Adjusted R^2	0.025	
F Statistic	2.08 (1; 41)	
(Intercept)	3.03	5.22×10^{-5}
Estimate - Change from Baseline	−0.011	0.40
PBA Irritability	0.48	0.00057
PBA Anxiety	0.49	0.00034
Observations	43	
R^2	0.51	
Adjusted R^2	0.48	
F Statistic	13.75^{***} (df = 3; 39)	

PBA - Problem Behaviours Assessment, HADS - Hospital Anxiety & Depression Scale

Table 25: Post-Task Estimate and AES Score

	Dependent variable: AES	
	Estimate	P Value
(Intercept)	3.46	$<2\text{x}10^{-16}$
Post-Task Estimate	0.0028	0.17
Observations	44	
Log Likelihood	-304.43	
Akaike Inf.Crit	310.43	
(Intercept)	2.83	$<2\text{x}10^{-16}$
Post-Task Estimate	0.0025	0.10
Age	0.0042	0.36
TMS	0.011	0.0001
Observations	44	
Log Likelihood	-282.71	
Akaike Inf.Crit	292.71	

AES - Apathy Evaluation Scale, TMS - Total Motor Score

Table 26: Estimate Change from Baseline and AES Score

	Dependent variable: AES	
	Estimate	P Value
(Intercept)	3.46	$<2\text{x}10^{-16}$
Estimate - Change from Baseline	-0.0039	0.30
Observations	49	
Log Likelihood	-346.084	
Akaike Inf.Crit	352.08	
(Intercept)	3.18	$<2\text{x}10^{-16}$
Estimate - Change from Baseline	-0.00276	0.082
Sex (Male)	0.23	0.027
TMS	0.0085	0.00021
Observations	49	
Log Likelihood	-329.49	
Akaike Inf.Crit	339.49	

AES - Apathy Evaluation Scale, TMS - Total Motor Score

Table 27: Propensity Scoring Models - SSRI Comparator

	treatment1	treatment2	var	mean1	mean2	pop.sd	ks	ks.pval	stop.method
1	SSRI	NDRI	age	50.16	44.45	12.48	0.23	0.00	unw
2	SSRI	Phenylpiperazine	age	50.16	48.65	12.48	0.09	0.62	unw
3	SSRI	SNRI	age	50.16	50.27	12.48	0.02	0.98	unw
4	SSRI	TCA	age	50.16	54.26	12.48	0.18	0.00	unw
5	SSRI	TeCA	age	50.16	50.98	12.48	0.05	0.43	unw
6	SSRI	Unique	age	50.16	46.40	12.48	0.19	0.02	unw
7	NDRI	Phenylpiperazine	age	44.45	48.65	12.48	0.23	0.01	unw
8	NDRI	SNRI	age	44.45	50.27	12.48	0.24	0.00	unw
9	NDRI	TCA	age	44.45	54.26	12.48	0.41	0.00	unw
10	NDRI	TeCA	age	44.45	50.98	12.48	0.26	0.00	unw
11	NDRI	Unique	age	44.45	46.40	12.48	0.11	0.51	unw
12	Phenylpiperazine	SNRI	age	48.65	50.27	12.48	0.09	0.67	unw
13	Phenylpiperazine	TCA	age	48.65	54.26	12.48	0.24	0.00	unw
14	Phenylpiperazine	TeCA	age	48.65	50.98	12.48	0.11	0.41	unw
15	Phenylpiperazine	Unique	age	48.65	46.40	12.48	0.17	0.24	unw
16	SNRI	TCA	age	50.27	54.26	12.48	0.19	0.00	unw
17	SNRI	TeCA	age	50.27	50.98	12.48	0.05	0.63	unw
18	SNRI	Unique	age	50.27	46.40	12.48	0.20	0.02	unw
19	TCA	TeCA	age	54.26	50.98	12.48	0.20	0.00	unw
20	TCA	Unique	age	54.26	46.40	12.48	0.36	0.00	unw
21	TeCA	Unique	age	50.98	46.40	12.48	0.19	0.03	unw
22	SSRI	NDRI	age	50.19	44.64	12.48	0.30	0.00	es.max
23	SSRI	Phenylpiperazine	age	50.19	50.14	12.48	0.08	0.81	es.max
24	SSRI	SNRI	age	50.19	50.93	12.48	0.05	0.28	es.max
25	SSRI	TCA	age	50.19	52.99	12.48	0.21	0.22	es.max
26	SSRI	TeCA	age	50.19	50.39	12.48	0.04	0.91	es.max
27	SSRI	Unique	age	50.19	45.41	12.48	0.21	0.18	es.max
28	NDRI	Phenylpiperazine	age	44.64	50.14	12.48	0.34	0.00	es.max
29	NDRI	SNRI	age	44.64	50.93	12.48	0.34	0.00	es.max
30	NDRI	TCA	age	44.64	52.99	12.48	0.46	0.00	es.max
31	NDRI	TeCA	age	44.64	50.39	12.48	0.32	0.00	es.max
32	NDRI	Unique	age	44.64	45.41	12.48	0.14	0.77	es.max
33	Phenylpiperazine	SNRI	age	50.14	50.93	12.48	0.09	0.73	es.max
34	Phenylpiperazine	TCA	age	50.14	52.99	12.48	0.20	0.45	es.max
35	Phenylpiperazine	TeCA	age	50.14	50.39	12.48	0.08	0.87	es.max
36	Phenylpiperazine	Unique	age	50.14	45.41	12.48	0.25	0.16	es.max
37	SNRI	TCA	age	50.93	52.99	12.48	0.17	0.46	es.max
38	SNRI	TeCA	age	50.93	50.39	12.48	0.07	0.47	es.max
39	SNRI	Unique	age	50.93	45.41	12.48	0.24	0.10	es.max
40	TCA	TeCA	age	52.99	50.39	12.48	0.22	0.22	es.max
41	TCA	Unique	age	52.99	45.41	12.48	0.37	0.04	es.max
42	TeCA	Unique	age	50.39	45.41	12.48	0.22	0.20	es.max

	treatment1	treatment2	var	mean1	mean2	pop.sd	ks	ks.pval	stop.method
1	SSRI	NDRI	sex:m	0.39	0.31	0.49	0.08	0.00	unw
2	SSRI	Phenylpiperazine	sex:m	0.39	0.39	0.49	0.00	0.88	unw
3	SSRI	SNRI	sex:m	0.39	0.35	0.49	0.04	0.00	unw
4	SSRI	TCA	sex:m	0.39	0.43	0.49	0.04	0.00	unw
5	SSRI	TeCA	sex:m	0.39	0.46	0.49	0.07	0.00	unw
6	SSRI	Unique	sex:m	0.39	0.49	0.49	0.10	0.00	unw
7	NDRI	Phenylpiperazine	sex:m	0.31	0.39	0.49	0.08	0.00	unw
8	NDRI	SNRI	sex:m	0.31	0.35	0.49	0.05	0.00	unw
9	NDRI	TCA	sex:m	0.31	0.43	0.49	0.12	0.00	unw
10	NDRI	TeCA	sex:m	0.31	0.46	0.49	0.15	0.00	unw
11	NDRI	Unique	sex:m	0.31	0.49	0.49	0.18	0.00	unw
12	Phenylpiperazine	SNRI	sex:m	0.39	0.35	0.49	0.04	0.22	unw
13	Phenylpiperazine	TCA	sex:m	0.39	0.43	0.49	0.04	0.00	unw
14	Phenylpiperazine	TeCA	sex:m	0.39	0.46	0.49	0.07	0.00	unw
15	Phenylpiperazine	Unique	sex:m	0.39	0.49	0.49	0.10	0.01	unw
16	SNRI	TCA	sex:m	0.35	0.43	0.49	0.08	0.00	unw
17	SNRI	TeCA	sex:m	0.35	0.46	0.49	0.10	0.00	unw
18	SNRI	Unique	sex:m	0.35	0.49	0.49	0.14	0.00	unw
19	TCA	TeCA	sex:m	0.43	0.46	0.49	0.02	0.20	unw
20	TCA	Unique	sex:m	0.43	0.49	0.49	0.06	0.00	unw
21	TeCA	Unique	sex:m	0.46	0.49	0.49	0.04	0.00	unw
22	SSRI	NDRI	sex:m	0.38	0.37	0.49	0.02	0.00	es.max
23	SSRI	Phenylpiperazine	sex:m	0.38	0.41	0.49	0.02	0.51	es.max
24	SSRI	SNRI	sex:m	0.38	0.34	0.49	0.04	0.00	es.max
25	SSRI	TCA	sex:m	0.38	0.34	0.49	0.04	0.54	es.max
26	SSRI	TeCA	sex:m	0.38	0.45	0.49	0.07	0.00	es.max
27	SSRI	Unique	sex:m	0.38	0.43	0.49	0.05	0.05	es.max
28	NDRI	Phenylpiperazine	sex:m	0.37	0.41	0.49	0.04	0.00	es.max
29	NDRI	SNRI	sex:m	0.37	0.34	0.49	0.03	0.00	es.max
30	NDRI	TCA	sex:m	0.37	0.34	0.49	0.03	0.02	es.max
31	NDRI	TeCA	sex:m	0.37	0.45	0.49	0.08	0.00	es.max
32	NDRI	Unique	sex:m	0.37	0.43	0.49	0.07	0.00	es.max
33	Phenylpiperazine	SNRI	sex:m	0.41	0.34	0.49	0.07	0.01	es.max
34	Phenylpiperazine	TCA	sex:m	0.41	0.34	0.49	0.07	0.31	es.max
35	Phenylpiperazine	TeCA	sex:m	0.41	0.45	0.49	0.04	0.00	es.max
36	Phenylpiperazine	Unique	sex:m	0.41	0.43	0.49	0.03	0.10	es.max
37	SNRI	TCA	sex:m	0.34	0.34	0.49	0.00	0.45	es.max
38	SNRI	TeCA	sex:m	0.34	0.45	0.49	0.11	0.00	es.max
39	SNRI	Unique	sex:m	0.34	0.43	0.49	0.09	0.00	es.max
40	TCA	TeCA	sex:m	0.34	0.45	0.49	0.11	0.00	es.max
41	TCA	Unique	sex:m	0.34	0.43	0.49	0.09	0.06	es.max
42	TeCA	Unique	sex:m	0.45	0.43	0.49	0.01	0.91	es.max

	treatment1	treatment2	var	mean1	mean2	pop.sd	ks	ks.pval	stop.method
1	SSRI	NDRI	psych_comorbid	0.97	0.95	0.16	0.02	1.00	unw
2	SSRI	Phenylpiperazine	psych_comorbid	0.97	0.96	0.16	0.01	1.00	unw
3	SSRI	SNRI	psych_comorbid	0.97	0.98	0.16	0.01	1.00	unw
4	SSRI	TCA	psych_comorbid	0.97	0.98	0.16	0.01	1.00	unw
5	SSRI	TeCA	psych_comorbid	0.97	0.98	0.16	0.01	0.91	unw
6	SSRI	Unique	psych_comorbid	0.97	0.96	0.16	0.01	1.00	unw
7	NDRI	Phenylpiperazine	psych_comorbid	0.95	0.96	0.16	0.01	1.00	unw
8	NDRI	SNRI	psych_comorbid	0.95	0.98	0.16	0.02	0.93	unw
9	NDRI	TCA	psych_comorbid	0.95	0.98	0.16	0.02	0.98	unw
10	NDRI	TeCA	psych_comorbid	0.95	0.98	0.16	0.03	0.75	unw
11	NDRI	Unique	psych_comorbid	0.95	0.96	0.16	0.00	1.00	unw
12	Phenylpiperazine	SNRI	psych_comorbid	0.96	0.98	0.16	0.01	1.00	unw
13	Phenylpiperazine	TCA	psych_comorbid	0.96	0.98	0.16	0.01	1.00	unw
14	Phenylpiperazine	TeCA	psych_comorbid	0.96	0.98	0.16	0.02	1.00	unw
15	Phenylpiperazine	Unique	psych_comorbid	0.96	0.96	0.16	0.01	1.00	unw
16	SNRI	TCA	psych_comorbid	0.98	0.98	0.16	0.00	1.00	unw
17	SNRI	TeCA	psych_comorbid	0.98	0.98	0.16	0.01	1.00	unw
18	SNRI	Unique	psych_comorbid	0.98	0.96	0.16	0.02	1.00	unw
19	TCA	TeCA	psych_comorbid	0.98	0.98	0.16	0.01	1.00	unw
20	TCA	Unique	psych_comorbid	0.98	0.96	0.16	0.02	1.00	unw
21	TeCA	Unique	psych_comorbid	0.98	0.96	0.16	0.03	1.00	unw
22	SSRI	NDRI	psych_comorbid	0.97	0.97	0.16	0.00	1.00	es.max
23	SSRI	Phenylpiperazine	psych_comorbid	0.97	0.96	0.16	0.01	1.00	es.max
24	SSRI	SNRI	psych_comorbid	0.97	0.98	0.16	0.01	1.00	es.max
25	SSRI	TCA	psych_comorbid	0.97	0.97	0.16	0.00	1.00	es.max
26	SSRI	TeCA	psych_comorbid	0.97	0.98	0.16	0.01	1.00	es.max
27	SSRI	Unique	psych_comorbid	0.97	0.94	0.16	0.04	1.00	es.max
28	NDRI	Phenylpiperazine	psych_comorbid	0.97	0.96	0.16	0.01	1.00	es.max
29	NDRI	SNRI	psych_comorbid	0.97	0.98	0.16	0.01	1.00	es.max
30	NDRI	TCA	psych_comorbid	0.97	0.97	0.16	0.00	1.00	es.max
31	NDRI	TeCA	psych_comorbid	0.97	0.98	0.16	0.01	1.00	es.max
32	NDRI	Unique	psych_comorbid	0.97	0.94	0.16	0.04	1.00	es.max
33	Phenylpiperazine	SNRI	psych_comorbid	0.96	0.98	0.16	0.01	1.00	es.max
34	Phenylpiperazine	TCA	psych_comorbid	0.96	0.97	0.16	0.01	1.00	es.max
35	Phenylpiperazine	TeCA	psych_comorbid	0.96	0.98	0.16	0.02	1.00	es.max
36	Phenylpiperazine	Unique	psych_comorbid	0.96	0.94	0.16	0.03	1.00	es.max
37	SNRI	TCA	psych_comorbid	0.98	0.97	0.16	0.01	1.00	es.max
38	SNRI	TeCA	psych_comorbid	0.98	0.98	0.16	0.01	1.00	es.max
39	SNRI	Unique	psych_comorbid	0.98	0.94	0.16	0.04	0.99	es.max
40	TCA	TeCA	psych_comorbid	0.97	0.98	0.16	0.01	1.00	es.max
41	TCA	Unique	psych_comorbid	0.97	0.94	0.16	0.04	1.00	es.max
42	TeCA	Unique	psych_comorbid	0.98	0.94	0.16	0.05	0.97	es.max

	treatment1	treatment2	var	mean1	mean2	pop.sd	ks	ks.pval	stop.method
1	SSRI	NDRI	ssri_risk	0.17	0.26	0.39	0.09	0.00	unw
2	SSRI	Phenylpiperazine	ssri_risk	0.17	0.23	0.39	0.05	0.17	unw
3	SSRI	SNRI	ssri_risk	0.17	0.22	0.39	0.04	1.00	unw
4	SSRI	TCA	ssri_risk	0.17	0.20	0.39	0.02	0.55	unw
5	SSRI	TeCA	ssri_risk	0.17	0.18	0.39	0.00	1.00	unw
6	SSRI	Unique	ssri_risk	0.17	0.17	0.39	0.00	1.00	unw
7	NDRI	Phenylpiperazine	ssri_risk	0.26	0.23	0.39	0.03	0.85	unw
8	NDRI	SNRI	ssri_risk	0.26	0.22	0.39	0.04	0.08	unw
9	NDRI	TCA	ssri_risk	0.26	0.20	0.39	0.06	0.01	unw
10	NDRI	TeCA	ssri_risk	0.26	0.18	0.39	0.08	0.00	unw
11	NDRI	Unique	ssri_risk	0.26	0.17	0.39	0.09	0.03	unw
12	Phenylpiperazine	SNRI	ssri_risk	0.23	0.22	0.39	0.01	1.00	unw
13	Phenylpiperazine	TCA	ssri_risk	0.23	0.20	0.39	0.03	0.93	unw
14	Phenylpiperazine	TeCA	ssri_risk	0.23	0.18	0.39	0.05	0.25	unw
15	Phenylpiperazine	Unique	ssri_risk	0.23	0.17	0.39	0.05	0.61	unw
16	SNRI	TCA	ssri_risk	0.22	0.20	0.39	0.02	0.77	unw
17	SNRI	TeCA	ssri_risk	0.22	0.18	0.39	0.04	1.00	unw
18	SNRI	Unique	ssri_risk	0.22	0.17	0.39	0.04	0.51	unw
19	TCA	TeCA	ssri_risk	0.20	0.18	0.39	0.02	0.79	unw
20	TCA	Unique	ssri_risk	0.20	0.17	0.39	0.02	1.00	unw
21	TeCA	Unique	ssri_risk	0.18	0.17	0.39	0.00	1.00	unw
22	SSRI	NDRI	ssri_risk	0.17	0.31	0.39	0.14	0.00	es.max
23	SSRI	Phenylpiperazine	ssri_risk	0.17	0.23	0.39	0.06	0.16	es.max
24	SSRI	SNRI	ssri_risk	0.17	0.22	0.39	0.05	1.00	es.max
25	SSRI	TCA	ssri_risk	0.17	0.13	0.39	0.04	0.92	es.max
26	SSRI	TeCA	ssri_risk	0.17	0.19	0.39	0.02	0.43	es.max
27	SSRI	Unique	ssri_risk	0.17	0.12	0.39	0.05	0.81	es.max
28	NDRI	Phenylpiperazine	ssri_risk	0.31	0.23	0.39	0.08	0.11	es.max
29	NDRI	SNRI	ssri_risk	0.31	0.22	0.39	0.09	0.01	es.max
30	NDRI	TCA	ssri_risk	0.31	0.13	0.39	0.18	0.00	es.max
31	NDRI	TeCA	ssri_risk	0.31	0.19	0.39	0.12	0.00	es.max
32	NDRI	Unique	ssri_risk	0.31	0.12	0.39	0.18	0.00	es.max
33	Phenylpiperazine	SNRI	ssri_risk	0.23	0.22	0.39	0.00	1.00	es.max
34	Phenylpiperazine	TCA	ssri_risk	0.23	0.13	0.39	0.10	0.18	es.max
35	Phenylpiperazine	TeCA	ssri_risk	0.23	0.19	0.39	0.03	0.86	es.max
36	Phenylpiperazine	Unique	ssri_risk	0.23	0.12	0.39	0.10	0.12	es.max
37	SNRI	TCA	ssri_risk	0.22	0.13	0.39	0.09	0.09	es.max
38	SNRI	TeCA	ssri_risk	0.22	0.19	0.39	0.03	0.29	es.max
39	SNRI	Unique	ssri_risk	0.22	0.12	0.39	0.10	0.05	es.max
40	TCA	TeCA	ssri_risk	0.13	0.19	0.39	0.06	0.51	es.max
41	TCA	Unique	ssri_risk	0.13	0.12	0.39	0.01	1.00	es.max
42	TeCA	Unique	ssri_risk	0.19	0.12	0.39	0.07	0.37	es.max

	treatment1	treatment2	var	mean1	mean2	pop.sd	ks	ks.pval	stop.method
1	SSRI	NDRI	high_comorbid	0.43	0.53	0.50	0.10	0.00	unw
2	SSRI	Phenylpiperazine	high_comorbid	0.43	0.43	0.50	0.00	1.00	unw
3	SSRI	SNRI	high_comorbid	0.43	0.45	0.50	0.02	1.00	unw
4	SSRI	TCA	high_comorbid	0.43	0.50	0.50	0.07	0.00	unw
5	SSRI	TeCA	high_comorbid	0.43	0.43	0.50	0.00	1.00	unw
6	SSRI	Unique	high_comorbid	0.43	0.38	0.50	0.05	0.23	unw
7	NDRI	Phenylpiperazine	high_comorbid	0.53	0.43	0.50	0.10	0.00	unw
8	NDRI	SNRI	high_comorbid	0.53	0.45	0.50	0.08	0.00	unw
9	NDRI	TCA	high_comorbid	0.53	0.50	0.50	0.03	0.49	unw
10	NDRI	TeCA	high_comorbid	0.53	0.43	0.50	0.10	0.00	unw
11	NDRI	Unique	high_comorbid	0.53	0.38	0.50	0.15	0.00	unw
12	Phenylpiperazine	SNRI	high_comorbid	0.43	0.45	0.50	0.02	0.98	unw
13	Phenylpiperazine	TCA	high_comorbid	0.43	0.50	0.50	0.07	0.06	unw
14	Phenylpiperazine	TeCA	high_comorbid	0.43	0.43	0.50	0.00	1.00	unw
15	Phenylpiperazine	Unique	high_comorbid	0.43	0.38	0.50	0.05	0.60	unw
16	SNRI	TCA	high_comorbid	0.45	0.50	0.50	0.05	0.01	unw
17	SNRI	TeCA	high_comorbid	0.45	0.43	0.50	0.02	1.00	unw
18	SNRI	Unique	high_comorbid	0.45	0.38	0.50	0.07	0.04	unw
19	TCA	TeCA	high_comorbid	0.50	0.43	0.50	0.07	0.00	unw
20	TCA	Unique	high_comorbid	0.50	0.38	0.50	0.12	0.00	unw
21	TeCA	Unique	high_comorbid	0.43	0.38	0.50	0.05	0.33	unw
22	SSRI	NDRI	high_comorbid	0.43	0.49	0.50	0.06	0.06	es.max
23	SSRI	Phenylpiperazine	high_comorbid	0.43	0.42	0.50	0.01	1.00	es.max
24	SSRI	SNRI	high_comorbid	0.43	0.46	0.50	0.03	1.00	es.max
25	SSRI	TCA	high_comorbid	0.43	0.32	0.50	0.11	0.03	es.max
26	SSRI	TeCA	high_comorbid	0.43	0.44	0.50	0.01	1.00	es.max
27	SSRI	Unique	high_comorbid	0.43	0.46	0.50	0.03	1.00	es.max
28	NDRI	Phenylpiperazine	high_comorbid	0.49	0.42	0.50	0.07	0.16	es.max
29	NDRI	SNRI	high_comorbid	0.49	0.46	0.50	0.03	0.70	es.max
30	NDRI	TCA	high_comorbid	0.49	0.32	0.50	0.17	0.00	es.max
31	NDRI	TeCA	high_comorbid	0.49	0.44	0.50	0.05	0.22	es.max
32	NDRI	Unique	high_comorbid	0.49	0.46	0.50	0.03	0.99	es.max
33	Phenylpiperazine	SNRI	high_comorbid	0.42	0.46	0.50	0.04	0.58	es.max
34	Phenylpiperazine	TCA	high_comorbid	0.42	0.32	0.50	0.09	0.18	es.max
35	Phenylpiperazine	TeCA	high_comorbid	0.42	0.44	0.50	0.02	1.00	es.max
36	Phenylpiperazine	Unique	high_comorbid	0.42	0.46	0.50	0.04	0.98	es.max
37	SNRI	TCA	high_comorbid	0.46	0.32	0.50	0.13	0.00	es.max
38	SNRI	TeCA	high_comorbid	0.46	0.44	0.50	0.02	0.77	es.max
39	SNRI	Unique	high_comorbid	0.46	0.46	0.50	0.00	1.00	es.max
40	TCA	TeCA	high_comorbid	0.32	0.44	0.50	0.11	0.03	es.max
41	TCA	Unique	high_comorbid	0.32	0.46	0.50	0.13	0.06	es.max
42	TeCA	Unique	high_comorbid	0.44	0.46	0.50	0.02	1.00	es.max

	treatment1	treatment2	var	mean1	mean2	pop.sd	ks	ks.pval	stop.method
1	SSRI	NDRI	composite_t1	9.66	12.95	5.54	0.30	0.00	unw
2	SSRI	Phenylpiperazine	composite_t1	9.66	11.10	5.54	0.24	0.00	unw
3	SSRI	SNRI	composite_t1	9.66	10.09	5.54	0.07	0.05	unw
4	SSRI	TCA	composite_t1	9.66	6.31	5.54	0.30	0.00	unw
5	SSRI	TeCA	composite_t1	9.66	8.17	5.54	0.12	0.00	unw
6	SSRI	Unique	composite_t1	9.66	11.07	5.54	0.20	0.02	unw
7	NDRI	Phenylpiperazine	composite_t1	12.95	11.10	5.54	0.26	0.00	unw
8	NDRI	SNRI	composite_t1	12.95	10.09	5.54	0.29	0.00	unw
9	NDRI	TCA	composite_t1	12.95	6.31	5.54	0.55	-0.00	unw
10	NDRI	TeCA	composite_t1	12.95	8.17	5.54	0.40	0.00	unw
11	NDRI	Unique	composite_t1	12.95	11.07	5.54	0.25	0.01	unw
12	Phenylpiperazine	SNRI	composite_t1	11.10	10.09	5.54	0.23	0.00	unw
13	Phenylpiperazine	TCA	composite_t1	11.10	6.31	5.54	0.53	0.00	unw
14	Phenylpiperazine	TeCA	composite_t1	11.10	8.17	5.54	0.34	0.00	unw
15	Phenylpiperazine	Unique	composite_t1	11.10	11.07	5.54	0.23	0.07	unw
16	SNRI	TCA	composite_t1	10.09	6.31	5.54	0.31	0.00	unw
17	SNRI	TeCA	composite_t1	10.09	8.17	5.54	0.17	0.00	unw
18	SNRI	Unique	composite_t1	10.09	11.07	5.54	0.21	0.02	unw
19	TCA	TeCA	composite_t1	6.31	8.17	5.54	0.21	0.00	unw
20	TCA	Unique	composite_t1	6.31	11.07	5.54	0.36	0.00	unw
21	TeCA	Unique	composite_t1	8.17	11.07	5.54	0.30	0.00	unw
22	SSRI	NDRI	composite_t1	9.79	12.19	5.54	0.25	0.00	es.max
23	SSRI	Phenylpiperazine	composite_t1	9.79	11.03	5.54	0.24	0.00	es.max
24	SSRI	SNRI	composite_t1	9.79	10.20	5.54	0.07	0.11	es.max
25	SSRI	TCA	composite_t1	9.79	8.45	5.54	0.22	0.32	es.max
26	SSRI	TeCA	composite_t1	9.79	8.31	5.54	0.14	0.02	es.max
27	SSRI	Unique	composite_t1	9.79	11.90	5.54	0.25	0.08	es.max
28	NDRI	Phenylpiperazine	composite_t1	12.19	11.03	5.54	0.24	0.03	es.max
29	NDRI	SNRI	composite_t1	12.19	10.20	5.54	0.25	0.00	es.max
30	NDRI	TCA	composite_t1	12.19	8.45	5.54	0.43	0.01	es.max
31	NDRI	TeCA	composite_t1	12.19	8.31	5.54	0.38	0.00	es.max
32	NDRI	Unique	composite_t1	12.19	11.90	5.54	0.19	0.45	es.max
33	Phenylpiperazine	SNRI	composite_t1	11.03	10.20	5.54	0.22	0.01	es.max
34	Phenylpiperazine	TCA	composite_t1	11.03	8.45	5.54	0.41	0.01	es.max
35	Phenylpiperazine	TeCA	composite_t1	11.03	8.31	5.54	0.34	0.00	es.max
36	Phenylpiperazine	Unique	composite_t1	11.03	11.90	5.54	0.28	0.10	es.max
37	SNRI	TCA	composite_t1	10.20	8.45	5.54	0.24	0.23	es.max
38	SNRI	TeCA	composite_t1	10.20	8.31	5.54	0.18	0.00	es.max
39	SNRI	Unique	composite_t1	10.20	11.90	5.54	0.25	0.09	es.max
40	TCA	TeCA	composite_t1	8.45	8.31	5.54	0.18	0.60	es.max
41	TCA	Unique	composite_t1	8.45	11.90	5.54	0.33	0.16	es.max
42	TeCA	Unique	composite_t1	8.31	11.90	5.54	0.35	0.01	es.max

	treatment1	treatment2	var	mean1	mean2	pop.sd	ks	ks.pval	stop.method
1	SSRI	NDRI	sedative	0.70	0.66	0.45	0.04	0.01	unw
2	SSRI	Phenylpiperazine	sedative	0.70	0.82	0.45	0.12	0.00	unw
3	SSRI	SNRI	sedative	0.70	0.74	0.45	0.05	1.00	unw
4	SSRI	TCA	sedative	0.70	0.82	0.45	0.12	-0.00	unw
5	SSRI	TeCA	sedative	0.70	0.77	0.45	0.07	1.00	unw
6	SSRI	Unique	sedative	0.70	0.72	0.45	0.02	0.97	unw
7	NDRI	Phenylpiperazine	sedative	0.66	0.82	0.45	0.16	0.00	unw
8	NDRI	SNRI	sedative	0.66	0.74	0.45	0.09	0.00	unw
9	NDRI	TCA	sedative	0.66	0.82	0.45	0.17	-0.00	unw
10	NDRI	TeCA	sedative	0.66	0.77	0.45	0.12	0.00	unw
11	NDRI	Unique	sedative	0.66	0.72	0.45	0.07	0.12	unw
12	Phenylpiperazine	SNRI	sedative	0.82	0.74	0.45	0.07	0.01	unw
13	Phenylpiperazine	TCA	sedative	0.82	0.82	0.45	0.00	1.00	unw
14	Phenylpiperazine	TeCA	sedative	0.82	0.77	0.45	0.05	0.27	unw
15	Phenylpiperazine	Unique	sedative	0.82	0.72	0.45	0.10	0.03	unw
16	SNRI	TCA	sedative	0.74	0.82	0.45	0.08	0.00	unw
17	SNRI	TeCA	sedative	0.74	0.77	0.45	0.03	1.00	unw
18	SNRI	Unique	sedative	0.74	0.72	0.45	0.02	0.99	unw
19	TCA	TeCA	sedative	0.82	0.77	0.45	0.05	0.01	unw
20	TCA	Unique	sedative	0.82	0.72	0.45	0.10	0.00	unw
21	TeCA	Unique	sedative	0.77	0.72	0.45	0.05	0.35	unw
22	SSRI	NDRI	sedative	0.70	0.65	0.45	0.06	0.08	es.max
23	SSRI	Phenylpiperazine	sedative	0.70	0.81	0.45	0.11	0.00	es.max
24	SSRI	SNRI	sedative	0.70	0.73	0.45	0.03	1.00	es.max
25	SSRI	TCA	sedative	0.70	0.73	0.45	0.02	1.00	es.max
26	SSRI	TeCA	sedative	0.70	0.78	0.45	0.07	0.00	es.max
27	SSRI	Unique	sedative	0.70	0.75	0.45	0.05	0.71	es.max
28	NDRI	Phenylpiperazine	sedative	0.65	0.81	0.45	0.17	0.00	es.max
29	NDRI	SNRI	sedative	0.65	0.73	0.45	0.09	0.00	es.max
30	NDRI	TCA	sedative	0.65	0.73	0.45	0.08	0.30	es.max
31	NDRI	TeCA	sedative	0.65	0.78	0.45	0.13	0.00	es.max
32	NDRI	Unique	sedative	0.65	0.75	0.45	0.11	0.07	es.max
33	Phenylpiperazine	SNRI	sedative	0.81	0.73	0.45	0.08	0.01	es.max
34	Phenylpiperazine	TCA	sedative	0.81	0.73	0.45	0.09	0.25	es.max
35	Phenylpiperazine	TeCA	sedative	0.81	0.78	0.45	0.04	0.62	es.max
36	Phenylpiperazine	Unique	sedative	0.81	0.75	0.45	0.06	0.67	es.max
37	SNRI	TCA	sedative	0.73	0.73	0.45	0.00	1.00	es.max
38	SNRI	TeCA	sedative	0.73	0.78	0.45	0.04	0.03	es.max
39	SNRI	Unique	sedative	0.73	0.75	0.45	0.02	1.00	es.max
40	TCA	TeCA	sedative	0.73	0.78	0.45	0.05	0.83	es.max
41	TCA	Unique	sedative	0.73	0.75	0.45	0.03	1.00	es.max
42	TeCA	Unique	sedative	0.78	0.75	0.45	0.02	1.00	es.max

	treatment1	treatment2	var	mean1	mean2	pop.sd	ks	ks.pval	stop.method
1	SSRI	NDRI	fluox_dose	216.95	41.22	1358.38	0.35	0.00	unw
2	SSRI	Phenylpiperazine	fluox_dose	216.95	17.74	1358.38	0.85	0.00	unw
3	SSRI	SNRI	fluox_dose	216.95	113.72	1358.38	0.27	1.00	unw
4	SSRI	TCA	fluox_dose	216.95	376.23	1358.38	0.39	-0.00	unw
5	SSRI	TeCA	fluox_dose	216.95	140.57	1358.38	0.35	1.00	unw
6	SSRI	Unique	fluox_dose	216.95	383.55	1358.38	0.49	0.00	unw
7	NDRI	Phenylpiperazine	fluox_dose	41.22	17.74	1358.38	0.83	-0.00	unw
8	NDRI	SNRI	fluox_dose	41.22	113.72	1358.38	0.52	0.00	unw
9	NDRI	TCA	fluox_dose	41.22	376.23	1358.38	0.42	-0.00	unw
10	NDRI	TeCA	fluox_dose	41.22	140.57	1358.38	0.30	0.00	unw
11	NDRI	Unique	fluox_dose	41.22	383.55	1358.38	0.47	-0.00	unw
12	Phenylpiperazine	SNRI	fluox_dose	17.74	113.72	1358.38	0.89	0.00	unw
13	Phenylpiperazine	TCA	fluox_dose	17.74	376.23	1358.38	0.70	-0.00	unw
14	Phenylpiperazine	TeCA	fluox_dose	17.74	140.57	1358.38	0.66	0.00	unw
15	Phenylpiperazine	Unique	fluox_dose	17.74	383.55	1358.38	0.93	0.00	unw
16	SNRI	TCA	fluox_dose	113.72	376.23	1358.38	0.26	0.00	unw
17	SNRI	TeCA	fluox_dose	113.72	140.57	1358.38	0.59	1.00	unw
18	SNRI	Unique	fluox_dose	113.72	383.55	1358.38	0.31	0.00	unw
19	TCA	TeCA	fluox_dose	376.23	140.57	1358.38	0.50	0.00	unw
20	TCA	Unique	fluox_dose	376.23	383.55	1358.38	0.28	0.00	unw
21	TeCA	Unique	fluox_dose	140.57	383.55	1358.38	0.69	-0.00	unw
22	SSRI	NDRI	fluox_dose	223.99	81.38	1358.38	0.25	-0.00	es.max
23	SSRI	Phenylpiperazine	fluox_dose	223.99	20.87	1358.38	0.81	-0.00	es.max
24	SSRI	SNRI	fluox_dose	223.99	121.36	1358.38	0.21	1.00	es.max
25	SSRI	TCA	fluox_dose	223.99	233.89	1358.38	0.22	0.00	es.max
26	SSRI	TeCA	fluox_dose	223.99	541.33	1358.38	0.20	-0.00	es.max
27	SSRI	Unique	fluox_dose	223.99	243.62	1358.38	0.43	-0.00	es.max
28	NDRI	Phenylpiperazine	fluox_dose	81.38	20.87	1358.38	0.62	-0.00	es.max
29	NDRI	SNRI	fluox_dose	81.38	121.36	1358.38	0.20	0.00	es.max
30	NDRI	TCA	fluox_dose	81.38	233.89	1358.38	0.25	0.00	es.max
31	NDRI	TeCA	fluox_dose	81.38	541.33	1358.38	0.25	-0.00	es.max
32	NDRI	Unique	fluox_dose	81.38	243.62	1358.38	0.39	0.00	es.max
33	Phenylpiperazine	SNRI	fluox_dose	20.87	121.36	1358.38	0.79	-0.00	es.max
34	Phenylpiperazine	TCA	fluox_dose	20.87	233.89	1358.38	0.68	-0.00	es.max
35	Phenylpiperazine	TeCA	fluox_dose	20.87	541.33	1358.38	0.60	-0.00	es.max
36	Phenylpiperazine	Unique	fluox_dose	20.87	243.62	1358.38	0.91	-0.00	es.max
37	SNRI	TCA	fluox_dose	121.36	233.89	1358.38	0.24	0.00	es.max
38	SNRI	TeCA	fluox_dose	121.36	541.33	1358.38	0.24	-0.00	es.max
39	SNRI	Unique	fluox_dose	121.36	243.62	1358.38	0.34	-0.00	es.max
40	TCA	TeCA	fluox_dose	233.89	541.33	1358.38	0.21	0.00	es.max
41	TCA	Unique	fluox_dose	233.89	243.62	1358.38	0.38	0.00	es.max
42	TeCA	Unique	fluox_dose	541.33	243.62	1358.38	0.58	0.00	es.max

Table 28: Propensity Scoring Models - SNRI Comparator

	treatment1	treatment2	var	mean1	mean2	pop.sd	ks	ks.pval	stop.method
1	SNRI	NDRI	age	50.27	44.45	12.48	0.24	0.00	unw
2	SNRI	Phenylpiperazine	age	50.27	48.65	12.48	0.09	0.67	unw
3	SNRI	SSRI	age	50.27	50.16	12.48	0.02	0.98	unw
4	SNRI	TCA	age	50.27	54.26	12.48	0.19	0.00	unw
5	SNRI	TeCA	age	50.27	50.98	12.48	0.05	0.63	unw
6	SNRI	Unique	age	50.27	46.40	12.48	0.20	0.02	unw
7	NDRI	Phenylpiperazine	age	44.45	48.65	12.48	0.23	0.01	unw
8	NDRI	SSRI	age	44.45	50.16	12.48	0.23	0.00	unw
9	NDRI	TCA	age	44.45	54.26	12.48	0.41	0.00	unw
10	NDRI	TeCA	age	44.45	50.98	12.48	0.26	0.00	unw
11	NDRI	Unique	age	44.45	46.40	12.48	0.11	0.51	unw
12	Phenylpiperazine	SSRI	age	48.65	50.16	12.48	0.09	0.62	unw
13	Phenylpiperazine	TCA	age	48.65	54.26	12.48	0.24	0.00	unw
14	Phenylpiperazine	TeCA	age	48.65	50.98	12.48	0.11	0.41	unw
15	Phenylpiperazine	Unique	age	48.65	46.40	12.48	0.17	0.24	unw
16	SSRI	TCA	age	50.16	54.26	12.48	0.18	0.00	unw
17	SSRI	TeCA	age	50.16	50.98	12.48	0.05	0.43	unw
18	SSRI	Unique	age	50.16	46.40	12.48	0.19	0.02	unw
19	TCA	TeCA	age	54.26	50.98	12.48	0.20	0.00	unw
20	TCA	Unique	age	54.26	46.40	12.48	0.36	0.00	unw
21	TeCA	Unique	age	50.98	46.40	12.48	0.19	0.03	unw
22	SNRI	NDRI	age	50.93	44.64	12.48	0.34	0.00	es.max
23	SNRI	Phenylpiperazine	age	50.93	50.14	12.48	0.09	0.73	es.max
24	SNRI	SSRI	age	50.93	50.19	12.48	0.05	0.28	es.max
25	SNRI	TCA	age	50.93	52.99	12.48	0.17	0.46	es.max
26	SNRI	TeCA	age	50.93	50.39	12.48	0.07	0.47	es.max
27	SNRI	Unique	age	50.93	45.41	12.48	0.24	0.10	es.max
28	NDRI	Phenylpiperazine	age	44.64	50.14	12.48	0.34	0.00	es.max
29	NDRI	SSRI	age	44.64	50.19	12.48	0.30	0.00	es.max
30	NDRI	TCA	age	44.64	52.99	12.48	0.46	0.00	es.max
31	NDRI	TeCA	age	44.64	50.39	12.48	0.32	0.00	es.max
32	NDRI	Unique	age	44.64	45.41	12.48	0.14	0.77	es.max
33	Phenylpiperazine	SSRI	age	50.14	50.19	12.48	0.08	0.81	es.max
34	Phenylpiperazine	TCA	age	50.14	52.99	12.48	0.20	0.45	es.max
35	Phenylpiperazine	TeCA	age	50.14	50.39	12.48	0.08	0.87	es.max
36	Phenylpiperazine	Unique	age	50.14	45.41	12.48	0.25	0.16	es.max
37	SSRI	TCA	age	50.19	52.99	12.48	0.21	0.22	es.max
38	SSRI	TeCA	age	50.19	50.39	12.48	0.04	0.91	es.max
39	SSRI	Unique	age	50.19	45.41	12.48	0.21	0.18	es.max
40	TCA	TeCA	age	52.99	50.39	12.48	0.22	0.22	es.max
41	TCA	Unique	age	52.99	45.41	12.48	0.37	0.04	es.max
42	TeCA	Unique	age	50.39	45.41	12.48	0.22	0.20	es.max

	treatment1	treatment2	var	mean1	mean2	pop.sd	ks	ks.pval	stop.method
1	SNRI	NDRI	sex:m	0.35	0.31	0.49	0.05	0.00	unw
2	SNRI	Phenylpiperazine	sex:m	0.35	0.39	0.49	0.04	0.22	unw
3	SNRI	SSRI	sex:m	0.35	0.39	0.49	0.04	0.00	unw
4	SNRI	TCA	sex:m	0.35	0.43	0.49	0.08	0.00	unw
5	SNRI	TeCA	sex:m	0.35	0.46	0.49	0.10	0.00	unw
6	SNRI	Unique	sex:m	0.35	0.49	0.49	0.14	0.00	unw
7	NDRI	Phenylpiperazine	sex:m	0.31	0.39	0.49	0.08	0.00	unw
8	NDRI	SSRI	sex:m	0.31	0.39	0.49	0.08	0.00	unw
9	NDRI	TCA	sex:m	0.31	0.43	0.49	0.12	0.00	unw
10	NDRI	TeCA	sex:m	0.31	0.46	0.49	0.15	0.00	unw
11	NDRI	Unique	sex:m	0.31	0.49	0.49	0.18	0.00	unw
12	Phenylpiperazine	SSRI	sex:m	0.39	0.39	0.49	0.00	0.88	unw
13	Phenylpiperazine	TCA	sex:m	0.39	0.43	0.49	0.04	0.00	unw
14	Phenylpiperazine	TeCA	sex:m	0.39	0.46	0.49	0.07	0.00	unw
15	Phenylpiperazine	Unique	sex:m	0.39	0.49	0.49	0.10	0.01	unw
16	SSRI	TCA	sex:m	0.39	0.43	0.49	0.04	0.00	unw
17	SSRI	TeCA	sex:m	0.39	0.46	0.49	0.07	0.00	unw
18	SSRI	Unique	sex:m	0.39	0.49	0.49	0.10	0.00	unw
19	TCA	TeCA	sex:m	0.43	0.46	0.49	0.02	0.20	unw
20	TCA	Unique	sex:m	0.43	0.49	0.49	0.06	0.00	unw
21	TeCA	Unique	sex:m	0.46	0.49	0.49	0.04	0.00	unw
22	SNRI	NDRI	sex:m	0.34	0.37	0.49	0.03	0.00	es.max
23	SNRI	Phenylpiperazine	sex:m	0.34	0.41	0.49	0.07	0.01	es.max
24	SNRI	SSRI	sex:m	0.34	0.38	0.49	0.04	0.00	es.max
25	SNRI	TCA	sex:m	0.34	0.34	0.49	0.00	0.45	es.max
26	SNRI	TeCA	sex:m	0.34	0.45	0.49	0.11	0.00	es.max
27	SNRI	Unique	sex:m	0.34	0.43	0.49	0.09	0.00	es.max
28	NDRI	Phenylpiperazine	sex:m	0.37	0.41	0.49	0.04	0.00	es.max
29	NDRI	SSRI	sex:m	0.37	0.38	0.49	0.02	0.00	es.max
30	NDRI	TCA	sex:m	0.37	0.34	0.49	0.03	0.02	es.max
31	NDRI	TeCA	sex:m	0.37	0.45	0.49	0.08	0.00	es.max
32	NDRI	Unique	sex:m	0.37	0.43	0.49	0.07	0.00	es.max
33	Phenylpiperazine	SSRI	sex:m	0.41	0.38	0.49	0.02	0.51	es.max
34	Phenylpiperazine	TCA	sex:m	0.41	0.34	0.49	0.07	0.31	es.max
35	Phenylpiperazine	TeCA	sex:m	0.41	0.45	0.49	0.04	0.00	es.max
36	Phenylpiperazine	Unique	sex:m	0.41	0.43	0.49	0.03	0.10	es.max
37	SSRI	TCA	sex:m	0.38	0.34	0.49	0.04	0.54	es.max
38	SSRI	TeCA	sex:m	0.38	0.45	0.49	0.07	0.00	es.max
39	SSRI	Unique	sex:m	0.38	0.43	0.49	0.05	0.05	es.max
40	TCA	TeCA	sex:m	0.34	0.45	0.49	0.11	0.00	es.max
41	TCA	Unique	sex:m	0.34	0.43	0.49	0.09	0.06	es.max
42	TeCA	Unique	sex:m	0.45	0.43	0.49	0.01	0.91	es.max

	treatment1	treatment2	var	mean1	mean2	pop.sd	ks	ks.pval	stop.method
1	SNRI	NDRI	psych_comorbid	0.98	0.95	0.16	0.02	0.93	unw
2	SNRI	Phenylpiperazine	psych_comorbid	0.98	0.96	0.16	0.01	1.00	unw
3	SNRI	SSRI	psych_comorbid	0.98	0.97	0.16	0.01	1.00	unw
4	SNRI	TCA	psych_comorbid	0.98	0.98	0.16	0.00	1.00	unw
5	SNRI	TeCA	psych_comorbid	0.98	0.98	0.16	0.01	1.00	unw
6	SNRI	Unique	psych_comorbid	0.98	0.96	0.16	0.02	1.00	unw
7	NDRI	Phenylpiperazine	psych_comorbid	0.95	0.96	0.16	0.01	1.00	unw
8	NDRI	SSRI	psych_comorbid	0.95	0.97	0.16	0.02	1.00	unw
9	NDRI	TCA	psych_comorbid	0.95	0.98	0.16	0.02	0.98	unw
10	NDRI	TeCA	psych_comorbid	0.95	0.98	0.16	0.03	0.75	unw
11	NDRI	Unique	psych_comorbid	0.95	0.96	0.16	0.00	1.00	unw
12	Phenylpiperazine	SSRI	psych_comorbid	0.96	0.97	0.16	0.01	1.00	unw
13	Phenylpiperazine	TCA	psych_comorbid	0.96	0.98	0.16	0.01	1.00	unw
14	Phenylpiperazine	TeCA	psych_comorbid	0.96	0.98	0.16	0.02	1.00	unw
15	Phenylpiperazine	Unique	psych_comorbid	0.96	0.96	0.16	0.01	1.00	unw
16	SSRI	TCA	psych_comorbid	0.97	0.98	0.16	0.01	1.00	unw
17	SSRI	TeCA	psych_comorbid	0.97	0.98	0.16	0.01	0.91	unw
18	SSRI	Unique	psych_comorbid	0.97	0.96	0.16	0.01	1.00	unw
19	TCA	TeCA	psych_comorbid	0.98	0.98	0.16	0.01	1.00	unw
20	TCA	Unique	psych_comorbid	0.98	0.96	0.16	0.02	1.00	unw
21	TeCA	Unique	psych_comorbid	0.98	0.96	0.16	0.03	1.00	unw
22	SNRI	NDRI	psych_comorbid	0.98	0.97	0.16	0.01	1.00	es.max
23	SNRI	Phenylpiperazine	psych_comorbid	0.98	0.96	0.16	0.01	1.00	es.max
24	SNRI	SSRI	psych_comorbid	0.98	0.97	0.16	0.01	1.00	es.max
25	SNRI	TCA	psych_comorbid	0.98	0.97	0.16	0.01	1.00	es.max
26	SNRI	TeCA	psych_comorbid	0.98	0.98	0.16	0.01	1.00	es.max
27	SNRI	Unique	psych_comorbid	0.98	0.94	0.16	0.04	0.99	es.max
28	NDRI	Phenylpiperazine	psych_comorbid	0.97	0.96	0.16	0.01	1.00	es.max
29	NDRI	SSRI	psych_comorbid	0.97	0.97	0.16	0.00	1.00	es.max
30	NDRI	TCA	psych_comorbid	0.97	0.97	0.16	0.00	1.00	es.max
31	NDRI	TeCA	psych_comorbid	0.97	0.98	0.16	0.01	1.00	es.max
32	NDRI	Unique	psych_comorbid	0.97	0.94	0.16	0.04	1.00	es.max
33	Phenylpiperazine	SSRI	psych_comorbid	0.96	0.97	0.16	0.01	1.00	es.max
34	Phenylpiperazine	TCA	psych_comorbid	0.96	0.97	0.16	0.01	1.00	es.max
35	Phenylpiperazine	TeCA	psych_comorbid	0.96	0.98	0.16	0.02	1.00	es.max
36	Phenylpiperazine	Unique	psych_comorbid	0.96	0.94	0.16	0.03	1.00	es.max
37	SSRI	TCA	psych_comorbid	0.97	0.97	0.16	0.00	1.00	es.max
38	SSRI	TeCA	psych_comorbid	0.97	0.98	0.16	0.01	1.00	es.max
39	SSRI	Unique	psych_comorbid	0.97	0.94	0.16	0.04	1.00	es.max
40	TCA	TeCA	psych_comorbid	0.97	0.98	0.16	0.01	1.00	es.max
41	TCA	Unique	psych_comorbid	0.97	0.94	0.16	0.04	1.00	es.max
42	TeCA	Unique	psych_comorbid	0.98	0.94	0.16	0.05	0.97	es.max

	treatment1	treatment2	var	mean1	mean2	pop.sd	ks	ks.pval	stop.method
1	SNRI	NDRI	SSRI_risk	0.22	0.26	0.39	0.04	0.08	unw
2	SNRI	Phenylpiperazine	SSRI_risk	0.22	0.23	0.39	0.01	1.00	unw
3	SNRI	SSRI	SSRI_risk	0.22	0.17	0.39	0.04	1.00	unw
4	SNRI	TCA	SSRI_risk	0.22	0.20	0.39	0.02	0.77	unw
5	SNRI	TeCA	SSRI_risk	0.22	0.18	0.39	0.04	1.00	unw
6	SNRI	Unique	SSRI_risk	0.22	0.17	0.39	0.04	0.51	unw
7	NDRI	Phenylpiperazine	SSRI_risk	0.26	0.23	0.39	0.03	0.85	unw
8	NDRI	SSRI	SSRI_risk	0.26	0.17	0.39	0.09	0.00	unw
9	NDRI	TCA	SSRI_risk	0.26	0.20	0.39	0.06	0.01	unw
10	NDRI	TeCA	SSRI_risk	0.26	0.18	0.39	0.08	0.00	unw
11	NDRI	Unique	SSRI_risk	0.26	0.17	0.39	0.09	0.03	unw
12	Phenylpiperazine	SSRI	SSRI_risk	0.23	0.17	0.39	0.05	0.17	unw
13	Phenylpiperazine	TCA	SSRI_risk	0.23	0.20	0.39	0.03	0.93	unw
14	Phenylpiperazine	TeCA	SSRI_risk	0.23	0.18	0.39	0.05	0.25	unw
15	Phenylpiperazine	Unique	SSRI_risk	0.23	0.17	0.39	0.05	0.61	unw
16	SSRI	TCA	SSRI_risk	0.17	0.20	0.39	0.02	0.55	unw
17	SSRI	TeCA	SSRI_risk	0.17	0.18	0.39	0.00	1.00	unw
18	SSRI	Unique	SSRI_risk	0.17	0.17	0.39	0.00	1.00	unw
19	TCA	TeCA	SSRI_risk	0.20	0.18	0.39	0.02	0.79	unw
20	TCA	Unique	SSRI_risk	0.20	0.17	0.39	0.02	1.00	unw
21	TeCA	Unique	SSRI_risk	0.18	0.17	0.39	0.00	1.00	unw
22	SNRI	NDRI	SSRI_risk	0.22	0.31	0.39	0.09	0.01	es.max
23	SNRI	Phenylpiperazine	SSRI_risk	0.22	0.23	0.39	0.00	1.00	es.max
24	SNRI	SSRI	SSRI_risk	0.22	0.17	0.39	0.05	1.00	es.max
25	SNRI	TCA	SSRI_risk	0.22	0.13	0.39	0.09	0.09	es.max
26	SNRI	TeCA	SSRI_risk	0.22	0.19	0.39	0.03	0.29	es.max
27	SNRI	Unique	SSRI_risk	0.22	0.12	0.39	0.10	0.05	es.max
28	NDRI	Phenylpiperazine	SSRI_risk	0.31	0.23	0.39	0.08	0.11	es.max
29	NDRI	SSRI	SSRI_risk	0.31	0.17	0.39	0.14	0.00	es.max
30	NDRI	TCA	SSRI_risk	0.31	0.13	0.39	0.18	0.00	es.max
31	NDRI	TeCA	SSRI_risk	0.31	0.19	0.39	0.12	0.00	es.max
32	NDRI	Unique	SSRI_risk	0.31	0.12	0.39	0.18	0.00	es.max
33	Phenylpiperazine	SSRI	SSRI_risk	0.23	0.17	0.39	0.06	0.16	es.max
34	Phenylpiperazine	TCA	SSRI_risk	0.23	0.13	0.39	0.10	0.18	es.max
35	Phenylpiperazine	TeCA	SSRI_risk	0.23	0.19	0.39	0.03	0.86	es.max
36	Phenylpiperazine	Unique	SSRI_risk	0.23	0.12	0.39	0.10	0.12	es.max
37	SSRI	TCA	SSRI_risk	0.17	0.13	0.39	0.04	0.92	es.max
38	SSRI	TeCA	SSRI_risk	0.17	0.19	0.39	0.02	0.43	es.max
39	SSRI	Unique	SSRI_risk	0.17	0.12	0.39	0.05	0.81	es.max
40	TCA	TeCA	SSRI_risk	0.13	0.19	0.39	0.06	0.51	es.max
41	TCA	Unique	SSRI_risk	0.13	0.12	0.39	0.01	1.00	es.max
42	TeCA	Unique	SSRI_risk	0.19	0.12	0.39	0.07	0.37	es.max

	treatment1	treatment2	var	mean1	mean2	pop.sd	ks	ks.pval	stop.method
1	SNRI	NDRI	high_comorbid	0.45	0.53	0.50	0.08	0.00	unw
2	SNRI	Phenylpiperazine	high_comorbid	0.45	0.43	0.50	0.02	0.98	unw
3	SNRI	SSRI	high_comorbid	0.45	0.43	0.50	0.02	1.00	unw
4	SNRI	TCA	high_comorbid	0.45	0.50	0.50	0.05	0.01	unw
5	SNRI	TeCA	high_comorbid	0.45	0.43	0.50	0.02	1.00	unw
6	SNRI	Unique	high_comorbid	0.45	0.38	0.50	0.07	0.04	unw
7	NDRI	Phenylpiperazine	high_comorbid	0.53	0.43	0.50	0.10	0.00	unw
8	NDRI	SSRI	high_comorbid	0.53	0.43	0.50	0.10	0.00	unw
9	NDRI	TCA	high_comorbid	0.53	0.50	0.50	0.03	0.49	unw
10	NDRI	TeCA	high_comorbid	0.53	0.43	0.50	0.10	0.00	unw
11	NDRI	Unique	high_comorbid	0.53	0.38	0.50	0.15	0.00	unw
12	Phenylpiperazine	SSRI	high_comorbid	0.43	0.43	0.50	0.00	1.00	unw
13	Phenylpiperazine	TCA	high_comorbid	0.43	0.50	0.50	0.07	0.06	unw
14	Phenylpiperazine	TeCA	high_comorbid	0.43	0.43	0.50	0.00	1.00	unw
15	Phenylpiperazine	Unique	high_comorbid	0.43	0.38	0.50	0.05	0.60	unw
16	SSRI	TCA	high_comorbid	0.43	0.50	0.50	0.07	0.00	unw
17	SSRI	TeCA	high_comorbid	0.43	0.43	0.50	0.00	1.00	unw
18	SSRI	Unique	high_comorbid	0.43	0.38	0.50	0.05	0.23	unw
19	TCA	TeCA	high_comorbid	0.50	0.43	0.50	0.07	0.00	unw
20	TCA	Unique	high_comorbid	0.50	0.38	0.50	0.12	0.00	unw
21	TeCA	Unique	high_comorbid	0.43	0.38	0.50	0.05	0.33	unw
22	SNRI	NDRI	high_comorbid	0.46	0.49	0.50	0.03	0.70	es.max
23	SNRI	Phenylpiperazine	high_comorbid	0.46	0.42	0.50	0.04	0.58	es.max
24	SNRI	SSRI	high_comorbid	0.46	0.43	0.50	0.03	1.00	es.max
25	SNRI	TCA	high_comorbid	0.46	0.32	0.50	0.13	0.00	es.max
26	SNRI	TeCA	high_comorbid	0.46	0.44	0.50	0.02	0.77	es.max
27	SNRI	Unique	high_comorbid	0.46	0.46	0.50	0.00	1.00	es.max
28	NDRI	Phenylpiperazine	high_comorbid	0.49	0.42	0.50	0.07	0.16	es.max
29	NDRI	SSRI	high_comorbid	0.49	0.43	0.50	0.06	0.06	es.max
30	NDRI	TCA	high_comorbid	0.49	0.32	0.50	0.17	0.00	es.max
31	NDRI	TeCA	high_comorbid	0.49	0.44	0.50	0.05	0.22	es.max
32	NDRI	Unique	high_comorbid	0.49	0.46	0.50	0.03	0.99	es.max
33	Phenylpiperazine	SSRI	high_comorbid	0.42	0.43	0.50	0.01	1.00	es.max
34	Phenylpiperazine	TCA	high_comorbid	0.42	0.32	0.50	0.09	0.18	es.max
35	Phenylpiperazine	TeCA	high_comorbid	0.42	0.44	0.50	0.02	1.00	es.max
36	Phenylpiperazine	Unique	high_comorbid	0.42	0.46	0.50	0.04	0.98	es.max
37	SSRI	TCA	high_comorbid	0.43	0.32	0.50	0.11	0.03	es.max
38	SSRI	TeCA	high_comorbid	0.43	0.44	0.50	0.01	1.00	es.max
39	SSRI	Unique	high_comorbid	0.43	0.46	0.50	0.03	1.00	es.max
40	TCA	TeCA	high_comorbid	0.32	0.44	0.50	0.11	0.03	es.max
41	TCA	Unique	high_comorbid	0.32	0.46	0.50	0.13	0.06	es.max
42	TeCA	Unique	high_comorbid	0.44	0.46	0.50	0.02	1.00	es.max

	treatment1	treatment2	var	mean1	mean2	pop.sd	ks	ks.pval	stop.method
1	SNRI	NDRI	composite_t1	10.09	12.95	5.54	0.29	0.00	unw
2	SNRI	Phenylpiperazine	composite_t1	10.09	11.10	5.54	0.23	0.00	unw
3	SNRI	SSRI	composite_t1	10.09	9.66	5.54	0.07	0.05	unw
4	SNRI	TCA	composite_t1	10.09	6.31	5.54	0.31	0.00	unw
5	SNRI	TeCA	composite_t1	10.09	8.17	5.54	0.17	0.00	unw
6	SNRI	Unique	composite_t1	10.09	11.07	5.54	0.21	0.02	unw
7	NDRI	Phenylpiperazine	composite_t1	12.95	11.10	5.54	0.26	0.00	unw
8	NDRI	SSRI	composite_t1	12.95	9.66	5.54	0.30	0.00	unw
9	NDRI	TCA	composite_t1	12.95	6.31	5.54	0.55	-0.00	unw
10	NDRI	TeCA	composite_t1	12.95	8.17	5.54	0.40	0.00	unw
11	NDRI	Unique	composite_t1	12.95	11.07	5.54	0.25	0.01	unw
12	Phenylpiperazine	SSRI	composite_t1	11.10	9.66	5.54	0.24	0.00	unw
13	Phenylpiperazine	TCA	composite_t1	11.10	6.31	5.54	0.53	0.00	unw
14	Phenylpiperazine	TeCA	composite_t1	11.10	8.17	5.54	0.34	0.00	unw
15	Phenylpiperazine	Unique	composite_t1	11.10	11.07	5.54	0.23	0.07	unw
16	SSRI	TCA	composite_t1	9.66	6.31	5.54	0.30	0.00	unw
17	SSRI	TeCA	composite_t1	9.66	8.17	5.54	0.12	0.00	unw
18	SSRI	Unique	composite_t1	9.66	11.07	5.54	0.20	0.02	unw
19	TCA	TeCA	composite_t1	6.31	8.17	5.54	0.21	0.00	unw
20	TCA	Unique	composite_t1	6.31	11.07	5.54	0.36	0.00	unw
21	TeCA	Unique	composite_t1	8.17	11.07	5.54	0.30	0.00	unw
22	SNRI	NDRI	composite_t1	10.20	12.19	5.54	0.25	0.00	es.max
23	SNRI	Phenylpiperazine	composite_t1	10.20	11.03	5.54	0.22	0.01	es.max
24	SNRI	SSRI	composite_t1	10.20	9.79	5.54	0.07	0.11	es.max
25	SNRI	TCA	composite_t1	10.20	8.45	5.54	0.24	0.23	es.max
26	SNRI	TeCA	composite_t1	10.20	8.31	5.54	0.18	0.00	es.max
27	SNRI	Unique	composite_t1	10.20	11.90	5.54	0.25	0.09	es.max
28	NDRI	Phenylpiperazine	composite_t1	12.19	11.03	5.54	0.24	0.03	es.max
29	NDRI	SSRI	composite_t1	12.19	9.79	5.54	0.25	0.00	es.max
30	NDRI	TCA	composite_t1	12.19	8.45	5.54	0.43	0.01	es.max
31	NDRI	TeCA	composite_t1	12.19	8.31	5.54	0.38	0.00	es.max
32	NDRI	Unique	composite_t1	12.19	11.90	5.54	0.19	0.45	es.max
33	Phenylpiperazine	SSRI	composite_t1	11.03	9.79	5.54	0.24	0.00	es.max
34	Phenylpiperazine	TCA	composite_t1	11.03	8.45	5.54	0.41	0.01	es.max
35	Phenylpiperazine	TeCA	composite_t1	11.03	8.31	5.54	0.34	0.00	es.max
36	Phenylpiperazine	Unique	composite_t1	11.03	11.90	5.54	0.28	0.10	es.max
37	SSRI	TCA	composite_t1	9.79	8.45	5.54	0.22	0.32	es.max
38	SSRI	TeCA	composite_t1	9.79	8.31	5.54	0.14	0.02	es.max
39	SSRI	Unique	composite_t1	9.79	11.90	5.54	0.25	0.08	es.max
40	TCA	TeCA	composite_t1	8.45	8.31	5.54	0.18	0.60	es.max
41	TCA	Unique	composite_t1	8.45	11.90	5.54	0.33	0.16	es.max
42	TeCA	Unique	composite_t1	8.31	11.90	5.54	0.35	0.01	es.max

	treatment1	treatment2	var	mean1	mean2	pop.sd	ks	ks.pval	stop.method
1	SNRI	NDRI	sedative	0.74	0.66	0.45	0.09	0.00	unw
2	SNRI	Phenylpiperazine	sedative	0.74	0.82	0.45	0.07	0.01	unw
3	SNRI	SSRI	sedative	0.74	0.70	0.45	0.05	1.00	unw
4	SNRI	TCA	sedative	0.74	0.82	0.45	0.08	0.00	unw
5	SNRI	TeCA	sedative	0.74	0.77	0.45	0.03	1.00	unw
6	SNRI	Unique	sedative	0.74	0.72	0.45	0.02	0.99	unw
7	NDRI	Phenylpiperazine	sedative	0.66	0.82	0.45	0.16	0.00	unw
8	NDRI	SSRI	sedative	0.66	0.70	0.45	0.04	0.01	unw
9	NDRI	TCA	sedative	0.66	0.82	0.45	0.17	-0.00	unw
10	NDRI	TeCA	sedative	0.66	0.77	0.45	0.12	0.00	unw
11	NDRI	Unique	sedative	0.66	0.72	0.45	0.07	0.12	unw
12	Phenylpiperazine	SSRI	sedative	0.82	0.70	0.45	0.12	0.00	unw
13	Phenylpiperazine	TCA	sedative	0.82	0.82	0.45	0.00	1.00	unw
14	Phenylpiperazine	TeCA	sedative	0.82	0.77	0.45	0.05	0.27	unw
15	Phenylpiperazine	Unique	sedative	0.82	0.72	0.45	0.10	0.03	unw
16	SSRI	TCA	sedative	0.70	0.82	0.45	0.12	-0.00	unw
17	SSRI	TeCA	sedative	0.70	0.77	0.45	0.07	1.00	unw
18	SSRI	Unique	sedative	0.70	0.72	0.45	0.02	0.97	unw
19	TCA	TeCA	sedative	0.82	0.77	0.45	0.05	0.01	unw
20	TCA	Unique	sedative	0.82	0.72	0.45	0.10	0.00	unw
21	TeCA	Unique	sedative	0.77	0.72	0.45	0.05	0.35	unw
22	SNRI	NDRI	sedative	0.73	0.65	0.45	0.09	0.00	es.max
23	SNRI	Phenylpiperazine	sedative	0.73	0.81	0.45	0.08	0.01	es.max
24	SNRI	SSRI	sedative	0.73	0.70	0.45	0.03	1.00	es.max
25	SNRI	TCA	sedative	0.73	0.73	0.45	0.00	1.00	es.max
26	SNRI	TeCA	sedative	0.73	0.78	0.45	0.04	0.03	es.max
27	SNRI	Unique	sedative	0.73	0.75	0.45	0.02	1.00	es.max
28	NDRI	Phenylpiperazine	sedative	0.65	0.81	0.45	0.17	0.00	es.max
29	NDRI	SSRI	sedative	0.65	0.70	0.45	0.06	0.08	es.max
30	NDRI	TCA	sedative	0.65	0.73	0.45	0.08	0.30	es.max
31	NDRI	TeCA	sedative	0.65	0.78	0.45	0.13	0.00	es.max
32	NDRI	Unique	sedative	0.65	0.75	0.45	0.11	0.07	es.max
33	Phenylpiperazine	SSRI	sedative	0.81	0.70	0.45	0.11	0.00	es.max
34	Phenylpiperazine	TCA	sedative	0.81	0.73	0.45	0.09	0.25	es.max
35	Phenylpiperazine	TeCA	sedative	0.81	0.78	0.45	0.04	0.62	es.max
36	Phenylpiperazine	Unique	sedative	0.81	0.75	0.45	0.06	0.67	es.max
37	SSRI	TCA	sedative	0.70	0.73	0.45	0.02	1.00	es.max
38	SSRI	TeCA	sedative	0.70	0.78	0.45	0.07	0.00	es.max
39	SSRI	Unique	sedative	0.70	0.75	0.45	0.05	0.71	es.max
40	TCA	TeCA	sedative	0.73	0.78	0.45	0.05	0.83	es.max
41	TCA	Unique	sedative	0.73	0.75	0.45	0.03	1.00	es.max
42	TeCA	Unique	sedative	0.78	0.75	0.45	0.02	1.00	es.max

	treatment1	treatment2	var	mean1	mean2	pop.sd	ks	ks.pval	stop.method
1	SNRI	NDRI	fluox_dose	113.72	41.22	1358.38	0.52	0.00	unw
2	SNRI	Phenylpiperazine	fluox_dose	113.72	17.74	1358.38	0.89	0.00	unw
3	SNRI	SSRI	fluox_dose	113.72	216.95	1358.38	0.27	1.00	unw
4	SNRI	TCA	fluox_dose	113.72	376.23	1358.38	0.26	0.00	unw
5	SNRI	TeCA	fluox_dose	113.72	140.57	1358.38	0.59	1.00	unw
6	SNRI	Unique	fluox_dose	113.72	383.55	1358.38	0.31	0.00	unw
7	NDRI	Phenylpiperazine	fluox_dose	41.22	17.74	1358.38	0.83	-0.00	unw
8	NDRI	SSRI	fluox_dose	41.22	216.95	1358.38	0.35	0.00	unw
9	NDRI	TCA	fluox_dose	41.22	376.23	1358.38	0.42	-0.00	unw
10	NDRI	TeCA	fluox_dose	41.22	140.57	1358.38	0.30	0.00	unw
11	NDRI	Unique	fluox_dose	41.22	383.55	1358.38	0.47	-0.00	unw
12	Phenylpiperazine	SSRI	fluox_dose	17.74	216.95	1358.38	0.85	0.00	unw
13	Phenylpiperazine	TCA	fluox_dose	17.74	376.23	1358.38	0.70	-0.00	unw
14	Phenylpiperazine	TeCA	fluox_dose	17.74	140.57	1358.38	0.66	0.00	unw
15	Phenylpiperazine	Unique	fluox_dose	17.74	383.55	1358.38	0.93	0.00	unw
16	SSRI	TCA	fluox_dose	216.95	376.23	1358.38	0.39	-0.00	unw
17	SSRI	TeCA	fluox_dose	216.95	140.57	1358.38	0.35	1.00	unw
18	SSRI	Unique	fluox_dose	216.95	383.55	1358.38	0.49	0.00	unw
19	TCA	TeCA	fluox_dose	376.23	140.57	1358.38	0.50	0.00	unw
20	TCA	Unique	fluox_dose	376.23	383.55	1358.38	0.28	0.00	unw
21	TeCA	Unique	fluox_dose	140.57	383.55	1358.38	0.69	-0.00	unw
22	SNRI	NDRI	fluox_dose	121.36	81.38	1358.38	0.20	0.00	es.max
23	SNRI	Phenylpiperazine	fluox_dose	121.36	20.87	1358.38	0.79	-0.00	es.max
24	SNRI	SSRI	fluox_dose	121.36	223.99	1358.38	0.21	1.00	es.max
25	SNRI	TCA	fluox_dose	121.36	233.89	1358.38	0.24	0.00	es.max
26	SNRI	TeCA	fluox_dose	121.36	541.33	1358.38	0.24	-0.00	es.max
27	SNRI	Unique	fluox_dose	121.36	243.62	1358.38	0.34	-0.00	es.max
28	NDRI	Phenylpiperazine	fluox_dose	81.38	20.87	1358.38	0.62	-0.00	es.max
29	NDRI	SSRI	fluox_dose	81.38	223.99	1358.38	0.25	-0.00	es.max
30	NDRI	TCA	fluox_dose	81.38	233.89	1358.38	0.25	0.00	es.max
31	NDRI	TeCA	fluox_dose	81.38	541.33	1358.38	0.25	-0.00	es.max
32	NDRI	Unique	fluox_dose	81.38	243.62	1358.38	0.39	0.00	es.max
33	Phenylpiperazine	SSRI	fluox_dose	20.87	223.99	1358.38	0.81	-0.00	es.max
34	Phenylpiperazine	TCA	fluox_dose	20.87	233.89	1358.38	0.68	-0.00	es.max
35	Phenylpiperazine	TeCA	fluox_dose	20.87	541.33	1358.38	0.60	-0.00	es.max
36	Phenylpiperazine	Unique	fluox_dose	20.87	243.62	1358.38	0.91	-0.00	es.max
37	SSRI	TCA	fluox_dose	223.99	233.89	1358.38	0.22	0.00	es.max
38	SSRI	TeCA	fluox_dose	223.99	541.33	1358.38	0.20	-0.00	es.max
39	SSRI	Unique	fluox_dose	223.99	243.62	1358.38	0.43	-0.00	es.max
40	TCA	TeCA	fluox_dose	233.89	541.33	1358.38	0.21	0.00	es.max
41	TCA	Unique	fluox_dose	233.89	243.62	1358.38	0.38	0.00	es.max
42	TeCA	Unique	fluox_dose	541.33	243.62	1358.38	0.58	0.00	es.max

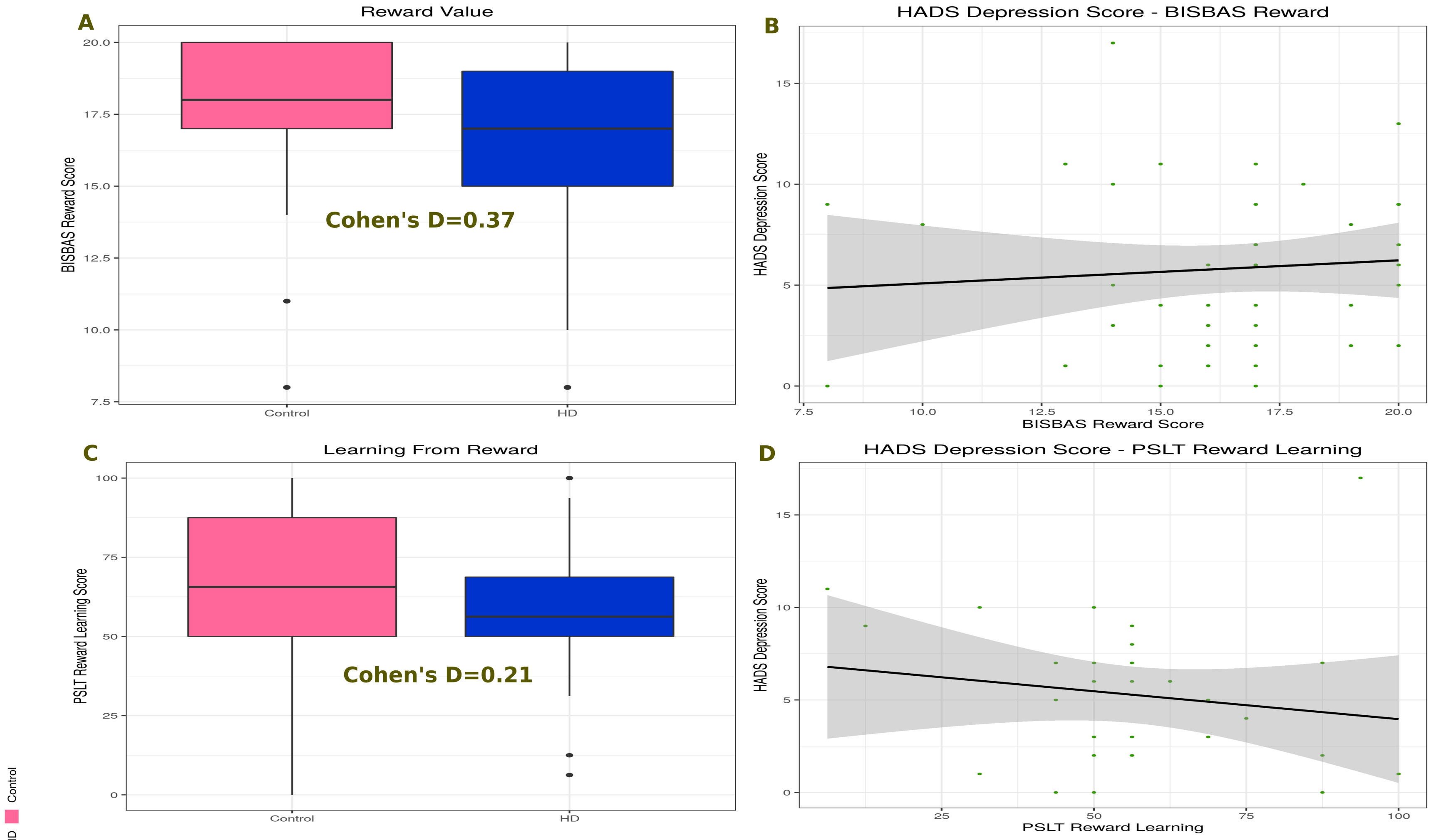

Supplementary Figure 1 - Reward Value and Reward Learning: Group Comparisons and Association with Depression Score

Group Comparison of reward value 3A)(χ2=1.16,p=0.28, N=77) and learning from reward 3C)(χ2=0.34,p=0.56, N=61), and prediction of HADS depression score from reward value 3B)(χ2=0,p=1,N=51) and learning from reward 3D)(χ2=0.57,p=.45,N=35) measures. Y-axis PSLT score 0-100, BAS Reward Score 0-20 (no relevant units).

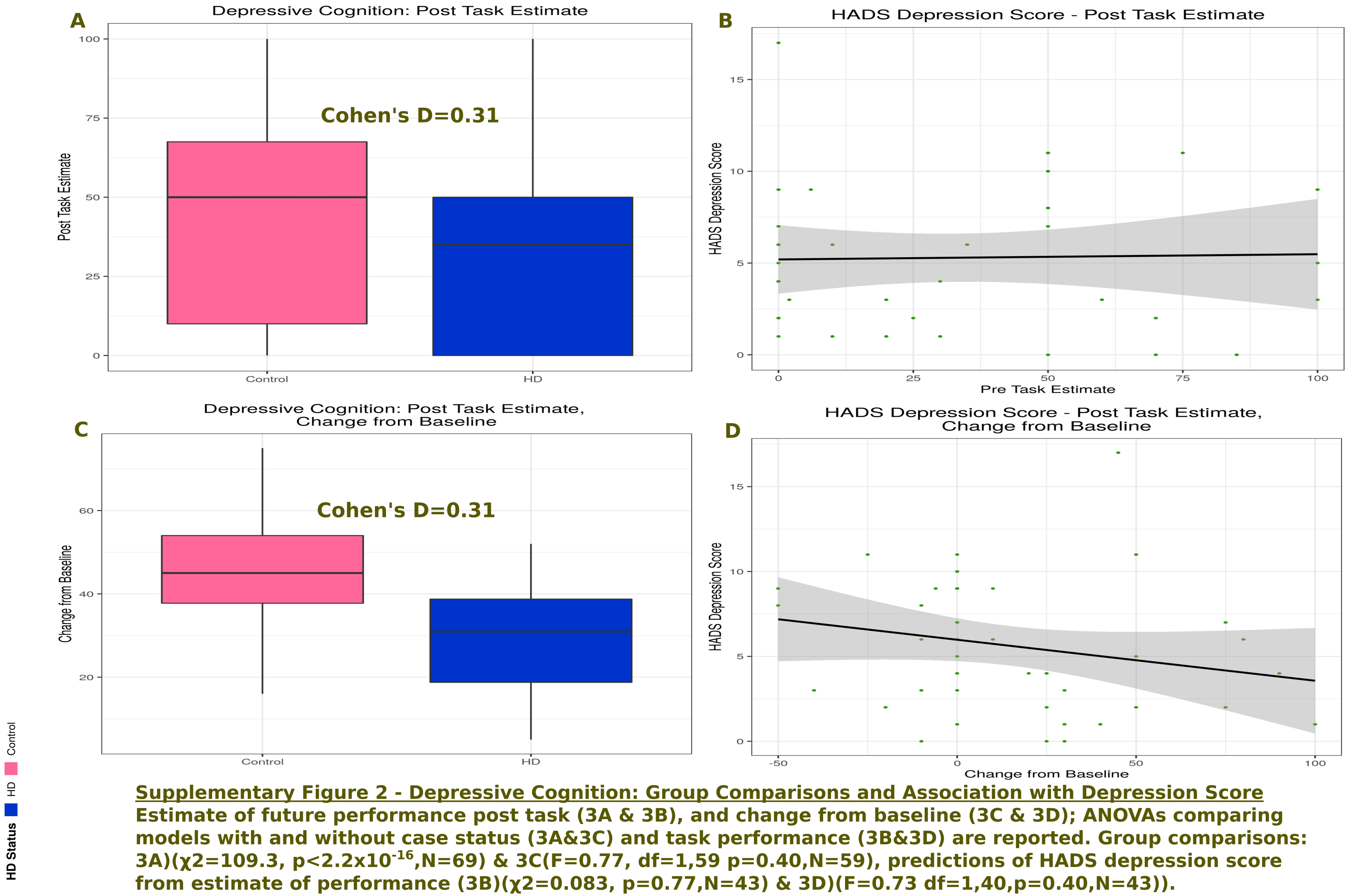

Supplementary Figure 2 - Depressive Cognition: Group Comparisons and Association with Depression Score
Estimate of future performance post task (3A & 3B), and change from baseline (3C & 3D); ANOVAs comparing models with and without case status (3A&3C) and task performance (3B&3D) are reported. Group comparisons: 3A)($\chi 2=109.3$, $p<2.2x10^{-16}$,N=69) & 3C(F=0.77, df=1,59 p=0.40,N=59), predictions of HADS depression score from estimate of performance (3B)($\chi 2=0.083$, p=0.77,N=43) & 3D)(F=0.73 df=1,40,p=0.40,N=43)).
Y-axis Change from Baseline -100 to 100, Post Task Estimate 0-100 (no relevant units).
